# Redistributive effects of fiscal policies in Mexico: Corrections for top income measurement problems

**DOI:** 10.1111/lamp.12206

**Published:** 2021-06-10

**Authors:** Vladimir Hlasny

**Affiliations:** ^1^ Economics Department Ewha Womans University Seoul South Korea

**Keywords:** ENIGH, fiscal incidence, Mexico, redistribution, top‐income measurement problems

## Abstract

This article assesses the redistributive effects of fiscal instruments in Mexico in 2010–2014, correcting for top‐income measurement problems. Two correction methods are applied—survey‐sample reweighting for households' nonresponse probability and replacing of top incomes using smooth Pareto distributions—to reestimate the effects of pensions, transfers, taxes, and subsidies. These corrections yield higher inequality measures, consistent between the reweighting and replacing methods. Taxable income shows the highest inequality and undergoes the highest upward correction for top‐income problems, whereas nontaxable income is strongly equalizing. Contributory pensions are inequality‐neutral, while transfers, taxes, and subsidies are equalizing. In‐kind transfers, cash‐like transfers, and direct taxes have the strongest equalizing effects. Top‐income measurement challenges retain their magnitude across years 2010, 2012, and 2014, but household nonresponse becomes more positively selected, causing greater biases in later years.

## Abstract (es)

Este artículo evalúa los efectos redistributivos de los instrumentos fiscales en México entre los años 2010 y 2014, corrigiendo los problemas de medición de los ingresos más altos. Se aplican dos métodos de corrección: la reponderación de la encuesta para los hogares con probabilidad de no respuesta y el reemplazo de los ingresos más altos mediante distribuciones de Pareto, para re‐estimar los efectos de las pensiones, transferencias, impuestos y subsidios. Estas correcciones producen medidas de desigualdad más altas, coherentes entre los métodos de reponderación y sustitución. El ingreso gravable muestra la mayor desigualdad y se somete a la mayor corrección al alza para los problemas de ingresos superiores, mientras que la renta no gravable está fuertemente igualada. Las pensiones son neutrales en materia de desigualdad, mientras que las transferencias, los impuestos y los subsidios tienden a igualar. Las transferencias en especie, las transferencias de ingreso y los impuestos directos tienen los efectos más fuertes en materia de igualdad. Los desafíos de medición de los ingresos superiores conservan su magnitud a lo largo de los años 2010, 2012 y 2014, pero la falta de respuesta de los hogares se selecciona de manera más positiva, lo que provoca mayores sesgos en años posteriores.

## Abstract (zh)

本文评估了2010‐2014年间墨西哥财政工具的再分配效果, 纠正了最高收入衡量问题。应用了两种纠正法—针对家庭无应答可能性的调查‐样本重新加权法、和针对最高收入的平滑帕累托分布代替法, 以期重新估计养老金、转移支付、税和补助金的效果。这些纠正法一致得出了更高的不平等标准。应税收入显示了最高的不平等, 并在最高收入问题方面得到了最高的向上纠正, 而非应税收入产生了强烈的均衡作用。缴费型养老金在不平等方面是持平的, 而转移支付、税和补助金发挥了均衡作用。实物形式的转移支付、类似现金的转移支付、以及直接税的均衡效果最强。最高收入衡量问题在2010年、2012年和2014年保持了其影响, 但家庭无应答 (household nonresponse) 变得更经过正面挑选, 导致之后几年的偏差更大。

## INTRODUCTION

Mexico has a notoriously unequal society in several dimensions and according to a variety of indicators. At the same time, fiscal instruments of the Mexican state are very weak in correcting the inequality, due to a low redistributive effort—low shares of tax revenue and of social spending to gross domestic product (GDP)—and ineffective targeting of spending across income groups. The precise redistributive effect is best evaluated using survey data with household‐level information on income components. Yet, these data are known to suffer from various measurement problems, particularly in surveys from developing countries such as Mexico and particularly among observations at the bottom and top of the income scale. Recent methodological literature has proposed advanced methods to correct for some types of measurement problems, using information from within or outside of the surveys. These methods have led to nontrivial corrections to the distribution of incomes and have typically increased the estimated inequality.

This study contributes by applying the correction methods to reassess the redistributive effects of fiscal policy in Mexico. We rely primarily on the 2014 version of the Mexican household budget survey (*Encuesta Nacional de Ingresos y Gastos de los Hogares*, ENIGH), but we cross‐validate our results using also the 2010 and 2012 versions and comment on trends going forward to years 2016 and 2018. We implement two types of correction methods, reweighting of the survey sample to correct for households' estimated nonresponse probability and replacing of potentially contaminated top incomes using synthetic estimates from smooth statistical distributions. Both of these methods rely on information within the survey—survey response rates at the level of Mexican states or the actual versus theoretically expected dispersion of top incomes, respectively.

The first approach (Korinek et al., 2006, 2007; Mistiaen & Ravallion, 2003) aims to inflate the weights of surveyed households to make them encompass even the mass of similar same‐region households that have not responded to the survey, according to an estimated behavioral response‐probability function. The second approach (Cowell & Flachaire, 2007; Cowell & Victoria‐Feser, 1996a, 1996b; Davidson & Flachaire, 2007) mitigates the influence of individual income observations to inequality measurement by replacing them with synthetic values derived from estimated parametric distributions. This method aims to avoid the problems of data contamination by misreporting and distortion of the income distribution by unit or item nonresponse. The method draws on a long‐established literature confirming that the top tail of income distributions worldwide is well approximated by a general family of statistical distributions. In this study, the replacing of top incomes is implemented either on all core income concepts, or on net market income alone, from which it is passed on to other income concepts through fiscal rules encompassed by the Commitment to Equity (CEQ) (Lustig, 2018) methodology.

After applying the corrections to eight core income concepts in Mexico—market, market + pensions, gross, taxable, net market, disposable, consumable, and final incomes per capita—we estimate the corrected income distributions and inequality measures. With the corrected income distributions, we estimate the redistributive effects of six different fiscal instruments on Mexican households—contributory pensions, cash‐like transfers, direct taxes, indirect taxes, subsidies, and in‐kind transfers. As a byproduct, we can comment on the relative redistributive role of taxable and nontaxable income to infer something about the redistributive effort by the Mexican state, and on the informality, tax evasion, and access to fiscal resources in the Mexican economy. Finally, we cross‐validate our results using the 2010 and 2012 versions of the ENIGH (CEQ Data Center, n.d.; LIS Data Center n.d.) and comment on the persistence and trends in top‐income measurement problems and implications for the measurement of redistributive effects.

We find that the corrections for potentially contaminated top incomes (replacing) and for household nonresponse (reweighting) yield higher inequality measures consistently, in both the replacing and reweighting method. Taxable income exhibits the highest inequality, furthering the highest upward correction for top‐income problems, whereas nontaxable income is strongly equalizing. Contributory pensions are inequality‐neutral, while transfers, taxes, and subsidies are equalizing. In‐kind transfers, cash‐like transfers, and direct taxes have the strongest equalizing impacts, at 4.7–5.7, 1.6–1.9, and 1.2–2.2 points of the Gini, respectively. Top‐income contamination and nonresponse retain their magnitude across the years 2010, 2012, and 2014, but household nonresponse becomes more positively selected, causing greater biases in later years. This finding is confirmed using aggregate statistics in the documentation for year 2016 and 2018 surveys.

The rest of our study is organized as follows. Section 2 reviews the existing evidence of income inequality in Mexico, inequality measurement under income reporting problems, and redistributive effects of Mexican fiscal policy instruments. Section 3 introduces two recently proposed techniques correcting for income mismeasurement, and Section 4 introduces briefly the Mexican household budget survey data. Section 5 presents our main findings, and Section 6 discusses their policy implications.

## LITERATURE REVIEW

Mexico, like Latin America at large, faces notoriously high economic inequality, and a growing body of literature has attempted to measure its degree, nature, and sources. Distinct branches of this literature have tackled the sources of inequality in labor and nonlabor market earnings, the redistributive effects of various fiscal instruments, and the measurement challenges faced by tax authorities and survey administrators. This section reviews briefly these branches of literature, their broad findings, and our contributions to the current state of knowledge.

### The degree and pattern of income inequality

Income inequality in Mexico rose sharply between the 1980s and 1990s, amid the austerity triggered by the 1980s debt crisis and structural reforms (Lustig, 1998). Inequality then fell in the 2000s, mirroring the trends across Latin America (Burdín et al., 2020). From 2003 to 2008, the Mexican economy grew rapidly in a way that benefited the poor as well as the middle class. The returns to higher education as seen through labor earnings fell. During the 2008–2010 recession, demand for low‐skill workers fell, and their purchasing power was held down by a stagnating real minimum wage, fewer progressive public transfers, and rising indirect taxes (Campos‐Vázquez et al., 2018).

The Mexican public sector suffered from a dramatic fall and slow recovery of oil prices, and a weak fiscal position including high public debt, declining tax revenues as share of GDP, and growing deficits (Martorano, 2014). These factors limited the space for monetary and fiscal responses to the crisis, even with the support of the International Monetary Fund and the World Bank (Ros, 2011). The Mexican government implemented various programs, including public works and retraining programs, but these initiatives had insufficient coverage and funding, due to restricted oil revenues (Freije et al., 2014). Recovery from the crisis was thus slow and uneven. Unemployment and the poverty rate soared, even though overall inequality fell slightly between 2006 and 2012. Since then, inequality has remained stagnant or continues to rise (Campos‐Vázquez et al., 2012; Cornia, 2014; Esquivel, 2011).

Despite the progress in the 2000s, inequality indicators, including the Gini coefficient, continue to make Mexico an unequal country. A thick right tail of the income distribution, and income shares of the top 0.5%–20% of households further attest to the high dispersion of incomes in Mexico. They place Mexico at the high end globally, nearly on par with Brazil, Colombia, Guatemala, Peru, South Africa, Uruguay, and the United States, according to some measures (Hlasny, 2020b). The inverted Pareto coefficient among the richest percentile of households, indicating the degree of top‐income dispersion, has been estimated at 2.6–2.7, and as high as 3.9 among the top 0.01% of households (Olascoaga, 2015).

Our study confirms these findings and offers additional evidence of inequality and top‐income dispersion during the years 2010, 2012, and 2014. We estimate inequality for multiple core income concepts, and correct them for two specific top‐income measurement problems—unit nonresponse, and income misreporting among top‐income households—using advanced but careful and tractable methods. Given the careful assumptions and parsimonious supplementary information used in estimation, the presented corrections should be viewed as conservative for the actual measurement biases.

### Income measurement problems in tax records and budget surveys

One challenge with assessing fiscal redistribution or measuring inequality in Mexico is that income data are widely suspected to suffer from statistical problems, including misreporting. A number of studies have acknowledged this fact, and some have attempted to address it in connection to measuring inequality. Among early investigations, Navarrete (1973) distinguished three strata in the household survey, according to the relationship between household incomes and expenditures and national accounts data. She applied an adjustment to reconcile incomes and expenditures in the strata where incomes under‐shot expenditures (ostensibly due to the omission of income in kind) and scaled up higher incomes proportionally to match national accounts data. Félix (1982) substituted consumption for income of the bottom 40% of households and scaled up the upper 60% of incomes to account for the survey's aggregate shortfall vis‐à‐vis the System of National Accounts (SNA).

Olascoaga (2015) estimated top income shares in Mexico between 2009 and 2012, using the 2010 Mexican National Household Income and Expenditure Survey (*Encuesta Nacional de Ingresos y Gastos de los Hogares*, ENIGH) as well as microdata with personal tax returns and employer‐wage returns. He found that high incomes in Mexico are not as unequally distributed as in other Latin American countries, but the topmost incomes exhibit greater dispersion. The estimated inverted Pareto coefficients among Mexican topmost incomes, from 2.6 to as high as 3.9, are large by international standards.

Bustos (2015) used the 2012 ENIGH (CEQ Data Center, n.d.), in combination with the Mexican SNA, to correct for biases due to income underreporting and underrepresentation of high‐income households. To that aim, he fitted the generalized beta (type II), gamma, generalized gamma, and log‐normal distributions. He concluded that the generalized beta distribution fit the data well and outperformed the alternative parametric distributions, even though he cautioned that the statistical significance of these results is unclear. He found that the official poverty estimate was robust to the choice of the correction method, but the Gini coefficient was highly sensitive to the parametric choices, rising from 0.44 to 0.52 under the gamma distribution, or to as high as 0.80 under the GB2.

Bustos and Leyva‐Parra (2017) adjusted 2012 ENIGH (CEQ Data Center, n.d.) survey incomes to make them compatible with the SNA and the Mexican Tax Administration Service records. They fitted generalized gamma and generalized beta (type II) distributions and found that these alternative specifications did not affect the measurement of inequality significantly. As a result of the correction, the Gini rose from 0.45 to 0.63, but the measure of poverty fell. Reyes et al. (2017) proposed a method for adjusting incomes to deal with income truncation in the top tail and underreporting of various income components in the rest of the distribution. This adjustment increased the Mexican Gini from 0.52 to 0.74, or even as high as 0.97, apparently making Mexico the most unequal country globally.

Campos‐Vázquez et al. (2018) used the 2012 version of the household survey, jointly with the national accounts, to reestimate inequality, accounting for the gap in aggregate income between the two sources. They estimated the residual income excluded from the household survey, assigning a share of it to the top decile, and the rest to the ninth decile. Using these new income shares of the top two deciles, they calculated the shape coefficient of the corresponding top‐income Pareto distribution and imputed the income shares of other fractiles of the top income distribution.

Alvaredo et al. (2017) corrected the ENIGH incomes for misreporting at the top by using a combination of taxpayer data, employer tax filings, national accounts statistics, and an employer survey (*Encuesta Nacional de Ocupación y Empleo*). They concluded that the top‐income shares in Mexico are among the highest in the world, with the top 10% of wage earners accounting for 45% of total wages, and top 10% of total‐income earners accounting for a staggering 59%–66% of aggregate income. Bourguignon (2018) also applies several alternative adjustment methods to the entire income distribution to correct it for missing income or missing respondents. Del Castillo Negrete Rovira (2017) reestimated the trend in inequality from 2004–2014 by scaling up survey incomes to meet national accounts statistics. Contrary to prior studies, he concluded that inequality was stagnant during the decade, or even rising from much lower levels in prior decades. Campos‐Vázquez and Lustig (2017) reached a similar conclusion, correcting income distributions from 2006 to 2017 for item nonresponse with hot‐deck imputation methods.

These existing studies give rise to vastly different estimates of the degree of inequality in Mexico. Leyva‐Parra (2004) sounded an early warning about the performance of alternative correction methods. He compared the existing approaches for realigning income distributions between household surveys and national accounts and critically examined the assumptions behind the methods. He cautioned that these assumptions were likely violated, giving rise to biases of potentially large magnitudes.

Our study contributes to this growing literature by performing two alternative tractable corrections to the top tail of incomes in the Mexican household survey, using benchmarks from within the survey. Our aim is not to incorporate heterogeneous external information or produce inequality estimates correcting for all suspected biases. Instead, by using methods based on limited assumptions and a controlled information set, our aim is to produce estimates corrected for the specific top‐income biases and linked clearly to specific modeling choices and comparison benchmarks. Alternative specifications and corrections performed on prior survey versions are presented for reference, to comment on the trend of inequality from 2010–2014. Finally, our study goes beyond correcting inequality indexes for various income concepts, also reassessing the redistributive effects of the core Mexican fiscal instruments.

### Effectiveness of fiscal redistribution

A growing number of studies have evaluated the fiscal redistribution system in Mexico and the prospect for using it to mitigate inequality. Existing fiscal policies have been found to correct inequality at most weakly, due to the low redistributive effort (low shares of tax revenue and of social spending to GDP) and ineffective targeting across income groups. Lindert et al. (2006) review the universality and composition of transfers in Mexico and report that 73% of Mexicans did not receive any social transfers, while some programs had benefits going all the way to the top income quintile.

Scott (2008, 2010, 2014) finds that a large share of transfers have limited redistributive effectiveness, including subsidies for social security pensions, exemptions to direct and indirect taxes, energy subsidies, and access to public higher education. Aranda and Scott (2016) report on the implications of public transfers for the poor and for ethnic minorities. Goñi et al. (2011) confirm that Mexico's fiscal system has a weak redistributive effect, in terms of both transfers and taxes. Mexico's fiscal revenues come largely from nontax rents (e.g., from the state‐owned oil company), and only limited revenues come from a narrow base of taxable units (e.g., tax evasion, and tax concessions and loopholes) (Goñi et al., 2011, p. 12). Indeed, government revenues account for a mere fifth of the Mexican GDP. Indirect taxes in Mexico are near proportional according to the share of market income they take up (Goñi et al., 2011, p. 16). Mexican public revenues are made up of nontaxed oil‐sector revenues and direct taxes, and only a small share comes from indirect and other taxes and social security contributions (Lustig, 2018, pp. 481–482).

The weak redistributive effect of transfers means that market income and gross income (i.e., adding taxable transfers) exhibit small differences in inequality, even by Latin American standards. Differences in inequality for gross income and disposable income (accounting for direct personal income taxes, and cash‐like transfers) are larger in absolute value, as well as by Latin American standards, but still much lower than is typical in Western Europe (Goñi et al., 2011, p. 7).

Fiscal redistribution affects measures of inequality in Mexico, due to high initial inequality in market incomes, but contributory social‐security pensions are somewhat unequalizing (Lustig, 2016). Disposable and post‐fiscal incomes appear to be distributed as widely as market incomes, confirming the poor effectiveness of direct transfers and taxes, and the neutral effect of indirect taxes (Lustig et al., 2019). At the same time, final incomes exhibit lower inequality, due to the effect of in‐kind education and health transfers.

When tax receipts and transfers are modestly increased, the distributional effect has been simulated to lower the Gini. Yet, were the tax structure to become more progressive or transfers more targeted, the Gini would fall only marginally, even compared to regional neighbors (Goñi et al., 2011). These results suggest that tax structure and transfer targeting have limited use as redistributive mechanisms under the current regime of a low tax base, low income tax productivity, and a mixed package of fiscal transfers, some of which are unequalizing.

One limitation of the studies reviewed in this section is that they rely on survey data that suffer from misreporting problems, especially at the top, or on tax authority data that do not capture all households or income sources, especially at the bottom. In one notable exception, in Ecuador, Jara and Oliva (2018) have explicitly addressed top‐income undercoverage in surveys, corrected incomes for it using information in administrative tax records, and used a microsimulation model to compare income inequality and total tax revenue. Our study takes a different approach, contributing by reestimating the distributional effect of multiple core fiscal instruments using household survey data, while correcting for distinct known types of top‐income biases. The following section explains the correction methods and their application to assessing the redistributive effects of fiscal instruments.

## METHODOLOGY

### Correcting inequality measures for household nonresponse

Household surveys are known to suffer from substantial and systematic nonresponse, which affects the observed distribution of household characteristics and outcomes. To correct the sampling weights and the income distribution in the Mexican survey for unit nonresponse, we apply a method proposed by Mistiaen and Ravallion (2003) and operationalized by Korinek et al. (2006, 2007) for the United States Current Population Survey and validated by Hlasny (2020b) and Hlasny and Verme (2018a, 2018b, 2018c) for other surveys worldwide. This method estimates households' response probabilities as a function of their observable characteristics, based on comparing the full distributions of these characteristics across regions with different mean response rates. The method then corrects households' sampling weights proportionally to their estimated inverse probability to respond, thus accounting for the density of nonresponding households expected to have similar characteristics as responding households. With these augmented weights, we can obtain a corrected distribution of household characteristics or outcomes, such as incomes.

At the heart of the method is a behavioral equation linking households' observable characteristics $x_{i}$ and their survey response probability $P_{i}$. Following existing studies, we model this probabilistic relationship as a logistic function of household characteristics $x_{i}$ multiplied by corresponding linear parameters θ:

(1) $$P_{i}\left(x_{i},\theta \right)=\frac{e^{g\left(x_{i},\theta \right)}}{1+e^{g\left(x_{i},\theta \right)}},$$

where $g\left(x_{i},\theta \right)$ is an arbitrary twice‐continuously differentiable function of $x_{i}$, such as the commonly used $g=\theta_{0}+\theta_{1}\text{log}\left(\mathrm{income}\right)$. The best‐fitting functional form will be selected from among various univariate and multivariate specifications. Note that estimating Equation (1) does not require any information on nonresponding households; rather, estimating $P_{i}$ allows us to reweight the responding units in such a way as to cover even the mass of nonresponders.

Estimating parameters θ allows us to infer each household's probability to respond to the survey ${\hat{P}}_{i}$ and thus also the number of households from which survey respondents are drawn according to sampling design. The predicted number of households in a sampling frame for a region, ${\hat{m}}_{j}$ can be derived as the sum of densities—or the inverted response probabilities ${\hat{P}}_{i}$ multiplied by households' sampling weights $s_{\mathrm{ij}}$—of all actually responding households in the region (written offhandedly as i∈j below to save on notation).

(2) $${\hat{m}}_{j}=\sum_{i\in j}{\hat{P}}_{\mathrm{ij}}^{‐1}s_{\mathrm{ij}}^{‐1}.$$

Comparing these model‐estimated populations ${\hat{m}}_{j}$ to known regional populations $m_{j}$, according to the ENIGH survey sampling design, and weighting their deviations by a weight proportional to regional sum of sampling weights and inversely proportional to regional populations, $w_{j}$ provides a measure of model fit. The best‐fitting coefficients $\hat{\theta }$ are those attaining the lowest weighted sum of squared population deviations:

(3) $$\hat{\theta }=\text{arg}\underset{\theta }{\text{min}}\sum_{j}{\left({\hat{m}}_{j}‐m_{j}\right)}^{2}/w_{j}.$$

Following Korinek et al.'s (2006, 2007) lead, we consider a number of specifications of the behavioral function of households' response. All models estimate households' survey‐response probability as a nonlinear function of their characteristics, among the characteristics available in the household budget survey.

### Correcting income distribution for potential misreporting among top incomes

Inequality measurement can be sensitive to the presence of even a few observations with misreported incomes (for example, due to data‐entry errors, underreporting by select individuals, or accounting rules that prescribe reporting of incomes when these are received rather than earned), or incomes distributed systematically differently than the true underlying values (perhaps due to top‐coding, or omission of a nonmonetary income category). One method to deal with these suspected problems is to replace the observed incomes with values obtained under an expected counterfactual distribution, where the counterfactual distribution can be identified from within the survey itself (including Cowell & Flachaire, 2007; Davidson & Flachaire, 2007).^1^

Because income misreporting is thought to be a problem particularly among top incomes, we can refer to an established literature on parametric approximations of the tails of empirical distributions. After replacing top‐income observations with parametric estimates, we can compute a corrected measure of inequality among them, using either known parametric properties of the fitted distribution or quasi‐nonparametrically or income values drawn randomly and repeatedly from the fitted distribution. The inequality measure for these incomes can readily be combined with a nonparametric inequality measure for lower incomes, to arrive at an index of overall inequality corrected for possible top incomes biases. Its standard error can be computed by bootstrapping the estimation routine.

One candidate for the parametric form is the Pareto distribution. More than a century ago, Vilfredo Pareto (1896) noted that top incomes tend to be distributed subject to systematic polynomial decay adequately described by a few parameters. This empirical tendency has since been confirmed across many countries and years. The probability density function of the Pareto (type I) distribution is

(4) $$f\left(x\right)=\frac{\alpha }{x^{\alpha +1}},1\leq x\leq \infty .$$

The parameter α can be estimated by maximum likelihood as

(5) $$\alpha =\frac{1}{k^{‐1}\sum_{i=0}^{k‐1}\log X_{\left(n‐i\right)}‐\log X_{\left(n‐k+1\right)}},$$

where *X_(j)_* is the *j*th order statistic in the sample of size n, and k is the count of observations classified as top incomes (Hill, 1975). The estimation can be modified to allow for lower and upper truncation in situations where only some range of incomes is considered uncontaminated by measurement problems, and distributed Pareto‐like. The estimation can also account for sampling weights.

An inverted equivalent of the Pareto parameter, often used as a measure of dispersion of top incomes, is $\beta =\alpha /\left(\alpha ‐1\right)$. The values of the inverted Pareto coefficients can be compared to those found worldwide (Atkinson et al., 2011). The Gini coefficient under the estimated Pareto distribution for the k top income observations can be derived parametrically as $Gini_{k}={\left(2\alpha ‐1\right)}^{‐1}$, and its standard error can be obtained by bootstrapping. This parametric Gini coefficient can then be combined with the nonparametric Gini for the *n*‐*k* lower income observations, $Gini_{n‐k}$, using geometric properties of the Lorenz curves as

(6) $$Gini=\left(1+Gini_{k}\right)\frac{k}{n}s_{k}‐\left(1‐Gini_{n‐k}\right)\left(1‐\frac{k}{n}\right)\left(1‐s_{k}\right)+\left(1‐\frac{2k}{n}\right),$$

where *s_k_* refers to the estimated share of aggregate income represented by the parametrically obtained *k* top incomes. *s_k_* is estimated using the approximation of the inverted Pareto coefficient as the ratio of the mean top income $\bar{X}$ to the cutoff point for replacement L ($\hat{\beta }=\bar{X}/L$), also known as van der Wijk's law (Atkinson et al., 2011). The mass of top incomes is thus estimated as $\hat{\beta }\times L\times k$.

The one‐parameter Pareto (type I) functional form has been evaluated positively, relative to more complex parametric choices, particularly at the topmost end of the income distribution.^2^ An empirical question concerns the appropriate lower (and upper) cutoff points for the estimation of the Pareto distribution, and the cutoff points for the replacement of observed incomes with parametric estimates (Hlasny & Verme, 2018a). Using data for the United Kingdom, Jenkins (2017) concludes that the preferred lower cutoff point for the estimation of the generalized Pareto (type II) distribution is between the 95th and the 99th percentile of incomes. For the one‐parameter Pareto (type I) distribution, the optimal cutoff may be at least as high or higher.

### Estimating the redistributive effect of fiscal policy: Top income measurement corrections

Household income components from various sources and fiscal adjustments reported in the household budget survey must be consistently combined to be comparable with income concepts used worldwide and to be informative regarding the marginal redistributive effect of various fiscal instruments. Following the standardized methodology of the Commitment to Equity Institute studies (CEQ),^3^ the following income concepts are adopted.

The broadest income concept representing households' *primary distribution* is *market income*, including all factor income sources, own production, imputed rent, and private transfers. Two alternative scenarios are distinguished regarding the character of contributory pensions: pensions are counted as pure transfers (excluded from *market income*) or as pure deferred market income (as *market income* +* pensions*).

To this primary distribution, *secondary redistribution* is applied by the state. Adding direct cash and near‐cash transfers (contributory pensions, conditional and unconditional cash transfers, school feeding programs, free food transfers, and more), we obtain *gross income*. As an alternative measure of total income from tax authorities' perspective, we can disregard nontaxable earnings and report *taxable income*.

Subtracting direct personal income taxes from *gross income*, we obtain *disposable income*. To see the effects of income taxation without the effects of public transfers, we can disregard cash transfers and report *net market income* after direct taxes. Using *disposable income*, subtracting indirect taxes (value added, excise, and others), and adding indirect subsidies (energy, food, and other price subsidies) we get post‐fiscal *consumable income*. Finally, adding monetized value of in‐kind transfers at average government cost (in Mexico, notably education and health), and subtracting co‐payments and user fees, we obtain *final income* (Lustig, 2018, pp. 17, 234).

Using this classification, we can identify the redistributive effects of various sets of fiscal interventions. Comparing the distribution of *market income* against *market income* + *pensions* shows the effect of contributory pensions. Comparing the distribution of *market income* + *pensions* against *gross income* shows the effect of cash‐like transfers. Comparing the distribution of *gross income* against *disposable income* shows the effect of income taxes (including on taxable transfers). Comparing the distribution of *disposable income* against *consumable income* shows the effect of indirect taxes and subsidies. Finally, comparing the distribution of *consumable income* against *final income* shows the net effects of participation in in‐kind programs.

We correct the redistributive effects for income measurement issues as follows. The reweighting approach is applied to each income concept of interest, to correct its distribution for unit nonresponse. In the behavioral equation of household response probability, market or net market income appears to perform the best as an explanatory variable, since it is easily observable by households and can influence their survey compliance behavior. The correction weights estimated in the model are then applied to all income concepts, to correct their respective distributions and thus to observe the redistributive effects of the fiscal adjustments linking them.

Under the replacing approach, two alternative modalities are applied. One, replacing is performed on each income concept in turn, and the respective corrected distributions are juxtaposed, to observe the redistributive effects of the fiscal interventions linking them. This approach corresponds to an assumption that all income concepts decay approximately according to the power law and that mismeasurements or contaminations can occur at any step in the transition from market income to final income. Indeed, prior literature has validated the Pareto approximation in various income concepts.

As an alternative, the replacing approach is applied to the distribution of net market incomes per capita, and fiscal adjustments are recalculated from the corrected distribution using the fiscal rules encompassed in the CEQ methodology. One reason for relying on net market income is that it is the starting point for the construction of all CEQ Core Income Concepts in the Mexican ENIGH, because it is this income that is directly lifted from household questionnaires. Two, net market incomes per capita are thought to satisfy the power law intrinsic in the Pareto distributions because they are strongly driven by market forces compared to post‐transfer and post‐subsidy incomes. Three, mismeasurement problems are thought to come primarily from misreporting of factor incomes, rather than from neglected legal loopholes, errors in eligibility determination, or households' selective participation in fiscal programs. Correcting for mismeasurements or contaminations in net‐market incomes then allows us to track accurately the redistributive effects of the consecutive fiscal interventions.

To implement the correction, the top tail of the net market income distribution is replaced with random draws from the estimated Pareto distribution and recombined with the bottom values of observed incomes. This pieced‐together corrected distribution is then passed on to the CEQ algorithms to recompute (in this order) taxable income, disposable income, consumable income, final income, gross income, and market income with or without pensions. The exercise is repeated 100 times to ensure that the results will not be contingent on a particular draw from the Pareto distribution.

### Quantifying the redistributive effects and the effect of measurement corrections

To quantify the redistributive effects of fiscal policies, two alternative inequality indicators are reported consistently for all analyses—the aggregate‐income shares of the top 1%, 5% and 10% of households, sensitive to how heavy the topmost tail is; and the Gini index, more sensitive to the dispersion of incomes near the middle of income distributions than in their tails. Generalized entropy indexes (0,1,2), and standard deviation, skewness, and kurtosis of incomes are calculated but not discussed. Percentage point differences in the Ginis and in the top‐income shares between pairs of income concepts are used as the central measures of the redistributive effects of the fiscal instruments linking them. It corresponds with the practice in existing fiscal incidence studies. In fact, the difference between pre‐fiscal and post‐fiscal Ginis is interpretated as the Reynolds–Smolensky index of vertical equity of tax and transfer systems (Reynolds & Smolensky, 2013).

The rest of this study uses household income per capita as the welfare aggregate, following he practices in existing academic and policy literature (Deaton, 1997, p. 150). Households are weighted by their post‐stratification sampling weights, accounting for their size.

## DATA

This study relies on the 100% sample of the 2014 version of the ENIGH administered by the National Institute of Statistics and Geography (*Instituto Nacional de Estadística y Geografía*, INEGI). Our version of survey microdata was obtained from the CEQ Institute (2020), which standardized survey variables with other national surveys and with the CEQ methodology and generated all core income concepts.

ENIGH is a high‐quality nationally representative survey of household wage and nonwage incomes, expenditures, and consumption. Table 1 provides descriptive statistics of per‐capita incomes in the ENIGH sample. Their visual inspection does not reveal the presence of any clear outliers or misreported values. Both the pre‐fiscal market income and the post‐fiscal disposable income exhibit a heavy rightmost tail relative to a reference lognormal distribution, but it is not on account of a few outliers. Instead, the entire upper tail is widely dispersed.^4^

**TABLE 1:** Income summary statistics, various income concepts, and uncorrected data

	(1)	(2)	(3)	(4)	(5)	(6)	(7)	(8)
	Market income per cap.	Market income + pensions per cap.	Gross income per cap.	Taxable income per cap.	Net market income per cap.	Disposable income per cap.	Consumable income per cap.	Final income per cap.
99.9th %ile	1,052,184	1,051,861	1,051,861	776,087	918,303	918,303	885,516	887,620
99th %ile	304,486	311,864	311,864	221,505	273,162	273,162	262,714	265,543
95th %ile	125,755	133,943	135,000	90,492	120,148	120,867	115,837	120,053
90th %ile	83,714	88,416	89,443	60,492	80,362	81,033	78,427	83,243
75th %ile	46,477	48,042	48,594	32,983	44,590	45,144	43,769	48,405
Mean	43,178	44,912	45,985	30,242	41,045	42,117	40,767	45,374
Median	25,983	26,941	27,747	18,010	25,443	26,286	25,659	30,340
25th %ile	14,685	15,298	16,642	9192	14,754	16,044	15,782	20,377
10th %ile	8115	8454	10,353	2779	8354	10,226	10,112	14,357
5th %ile	4822	5006	7323	492	4950	7255	7207	11,480
1st %ile	1751	1796	3944	0	1796	3903	3917	7098
Std. dev.	81,471	82,794	82,602	64,453	73,396	73,222	69,851	69,697
Skewness	18.29	17.53	17.63	27.29	20.75	20.88	20.57	20.63
Kurtosis	685.48	641.69	646.90	1456.07	925.56	933.40	909.34	914.54
Sample	73,508	73,508	73,508	73,508	73,508	73,508	73,508	73,508
Top 0.1% inc. share	3.65	3.54	3.46	3.93	3.26	3.18	3.14	2.82
0.1%–1% inc. share	9.94	9.67	9.45	10.41	9.27	9.04	8.93	8.08
1%–5% inc. share	16.72	16.98	16.64	17.16	16.55	16.20	16.05	14.75
5%–10% inc. share	11.79	11.95	11.74	12.07	11.80	11.58	11.53	10.87
Gini (HH‐size and sampling weighted data)	52.75	52.79	50.99	54.31	51.33	49.43	49.00	44.17
	(0.82)	(0.79)	(0.80)	(0.86)	(0.76)	(0.77)	(0.77)	(0.76)
Mean log dev. (GE0)	0.515 (0.009)	0.516 (0.009)	0.456 (0.008)	0.594 (0.010)	0.484 (0.008)	0.426 (0.008)	0.417 (0.008)	0.328 (0.007)
Theil index (GE1)	0.598 (0.020)	0.593 (0.019)	0.559 (0.019)	0.637 (0.024)	0.557 (0.018)	0.522 (0.018)	0.513 (0.018)	0.424 (0.016)
Half coef. of var. squared (GE2)	1.780 (0.198)	1.699 (0.184)	1.613 (0.176)	2.271 (0.365)	1.599 (0.207)	1.511 (0.197)	1.468 (0.190)	1.180 (0.154)

Note

Sampling‐weighted sample used. 0MXN incomes (19 household observations for market income, 7 for market + pensions and net market income, 2 for gross and disposable income, and 1465 for taxable income) are omitted in computations of the Gini. Gini standard errors are jackknife estimates on household‐level data (recognizing that household‐member incomes are copies of one another), accounting for household size, except in last row. Ginis and standard errors are multiplied by 100 for clarity of presentation.

Abbreviation: Per cap., per capita.

Unit nonresponse does appear to be a problem in the ENIGH, because 3.8% of households nationwide choose not to complete the survey (in addition to the other 6% of households listed falsely as residing in unoccupied dwellings, and 0.7% listed falsely as residing in what turn out to be invalid dwellings). The nonresponse rate varies by state, rising to 8%–9% in some (Coahuila, Guerrero, Jalisco, and San Luis Potosi). Nonresponding households are likely to be systematically selected from among units with rare characteristics and rare income levels relative to the responding units (for an explanation, see Mistiaen & Ravallion, 2003), and their omission may affect our measurement of the income distribution, inequality, and the redistributive effect of fiscal interventions. To the extent that the observed incomes do not capture the nonresponding rich households, the correlation between *all* incomes and nonresponse rates would be more positive.^5^

While ENIGH sampling weights provide some correction for unit nonresponse, INEGI does not provide raw components of the weights (beyond enumerating them), so we cannot evaluate the extent of this correction. Moreover, the existing correction is likely to be inadequate, because the weights are at the level of primary sampling units (PSUs), while each PSU contains heterogeneous households with vastly different income and demographic profiles, and different response probabilities. Applying the same weights to all households in a PSU means that each household's density is inflated slightly (by 3.8% on average across all PSUs) to incorporate the density of nonresponding units in the same PSU. Still, since nonresponding units are thought to come from the extremes of the income distribution, inflating all households' density uniformly and by such a small margin will not correct the income distribution sufficiently for the systematic omission of the extreme‐income households.

### Additional survey versions

Besides the 2014 dataset, we have two additional versions of the ENIGH at our disposal, ENIGH 2012 (CEQ Institute, 2020), and ENIGH 2010 (LIS Data Center, n.d.). Microdata from these surveys can be used for cross‐validation.^6^ Finally, documentation for the 2016 and 2018 ENIGH can be used to comment on the evolution of top‐income issues and measurement of fiscal redistributive effects over time. Unfortunately, microdata for the needed multiple income concepts are presently unavailable for these surveys. Across versions of the ENIGH, we find clearly that the household nonresponse problem increased in magnitude over time, particularly in the 2016 and 2018 versions. Total nonresponse rate was 10.1% in 2010; 11.8% in 2012; 10.6% in 2014; 15.0% in 2016; and 16.3% in 2018. Type A nonresponse was 2.9% in 2010; 2.5% in 2012; 3.8% in 2014; 7.8% in 2016; and 8.6% in 2018. Moreover, nonresponse appears to have become more consistently associated with households' incomes across survey versions. Correlation of statewide mean disposable income per capita and type A nonresponse rate was only 0.04 in 2010, whereas it was 0.24 in 2012, and 0.17 in 2014, suggesting that biases to the measurement of inequality may be significantly increasing over time.

Comparing the dispersion of top disposable incomes across survey versions, we find that 2012 incomes are distributed less smoothly, subject to a significant discontinuity of almost 500,000 Mexican pesos (or the 99.5th percentile), where the density suddenly drops off. The 2012 distribution covers an outlying individual whose per‐capita income exceeds 3 million Mexican pesos and is more than twice the following value. On the contrary, top disposable incomes in the 2010 sample have a spike in density above the 500,000 Mexican peso level.

The differences in nonresponse rates and their association with regional incomes suggest that the reweighting approach may produce more significant correction weights in recent surveys than in 2010. Yet, when applied to nonsmooth distributions of incomes with outliers, the question of how the corrections of inequality and of fiscal effects will compare across surveys is empirical. The replacing approach may be sensitive to the bunching of top incomes for the estimation of parameters and the correction of inequality measures.

## RESULTS

### The observed degree of inequality and redistributive effects of fiscal programs

According to the 2014 ENIGH (CEQ Data Center, n.d.), Mexican incomes per capita are distributed widely, subject to a high right skew, particularly among the richest few households. See Table 1. In the income distribution weighted using post‐stratification weights and household size, the top decile of Mexican households account for 42.1% of aggregate market income, or 36.5% of aggregate final income. The top ventile accounts for 30.3% of market income, or 25.7% of final income, and the top percentile accounts for 13.6% of market income, or 12.1% of final income. The Gini coefficient is 52.8 for market income, and falls to 49.4 and to 44.2, as one moves from market income to disposable income and to final income, respectively.^7^

Comparing columns in Table 1 confirms that contributory pensions have a neutral or slightly unequalizing effect on general inequality measured by the Gini (columns 1 and 2), while cash‐like transfers (columns 2 and 3), direct taxes (columns 3 and 6), indirect taxes and subsidies (columns 6 and 7), and in‐kind programs (columns 7 and 8) have equalizing effects. The Gini falls by 1.8 percentage points due to cash‐like transfers, by another 1.6 percentage points due to progressive income taxation, and by a significant 4.8 percentage points due to in‐kind programs. The effects on top‐income shares are analogous, and large in magnitude. Cash‐like transfers decrease the top 10% income share by 0.9 percentage points (0.3 percentage points for top 1% income share), direct taxes decrease the top 10% income share by 1.3 percentage points (0.7 percentage points for top 1% income share), and in‐kind transfers decrease the top 10% income share by 3.1 percentage points (1.2 percentage points for top 1% income share).

To evaluate how sensitive these findings are to top‐income measurement issues, the following sections correct the income distributions for two distinct types of expected measurement problems—unit nonresponse, and income misreporting—and reestimate the redistributive effects of fiscal interventions.

### Correcting for unit nonresponse by reweighting

First, we attempt to correct the income distribution for the tendency of wealthy households not to complete surveys. In this analysis, we disregard unoccupied or invalid dwellings and restrict our attention to households that were successfully contacted, because only their probability of survey response is amenable to behavioral analysis. Even among these households, we ignore instances when an interview was impossible due to climatological, political, social or security—that is, nonbehavioral—problems (13 out of 838 nonrespondents nationwide).

Table 2 reports the results of univariate model specifications, using as an explanatory variable household market, gross or disposable income, either at the household level or per capita. The estimated models are enumerated in the first column. The table shows the estimated values of the model intercept $\hat{\theta_{0}}$, slope coefficient on the income measure $\hat{\theta_{1}}$, and selected measures of model fit—sum of weighted squared deviations of regional populations, factor of proportionality $\sigma^{2}$ related to the typical squared deviation between predicted and actual regional populations, and the Akaike and Schwarz (Bayesian) information criteria. The Akaike information criterion is used to guide model selection because of its good consistency properties.

**TABLE 2:** Estimation results for various univariate logistic models of response probability

Specification of $g\left(x_{i},\theta \right)$	$\hat{\theta_{0}}$(*SE*)	$\hat{\theta_{1}}$(*SE*)	Sum of squared weighted errors	Factor of proportionality (σ^2^)	AIC SIC	Gini (*SE*): Market income per capita, weighted data
Uncorrected						52.75 (0.82)
1: *θ*_0_ + *θ*_1_log(market inc.)	14.464 (0.078)	−0.960 (0.006)	35,242	0.448	228.14	56.41 (1.92)
					225.52	
2: *θ*_0_ + *θ*_1_log(gross inc.)	14.685 (0.082)	−0.972 (0.007)	36,116	0.460	228.92	56.30 (1.90)
					226.31	
3: *θ*_0_ + *θ*_1_log(dispos. inc.)	16.087 (0.074)	−1.091 (0.006)	36,080	0.454	228.89	57.64 (2.71)
					226.27	
4: *θ*_0_ + *θ*_1_log(mkt. inc.pc)	8.667 (0.062)	−0.537 (0.006)	38,450	0.486	230.92	53.78 (0.98)
					228.31	
5: *θ*_0_ + *θ*_1_log(gross inc.pc)	8.594 (0.065)	−0.524 (0.006)	39,263	0.496	231.59	53.68 (0.96)
					228.98	
6: *θ*_0_ + *θ*_1_log(disp. inc.pc)	8.645 (0.067)	−0.532 (0.006)	39,412	0.499	231.72	53.65 (0.96)
					229.10	
7: *θ*_0_ + *θ*_1_log(market inc.)^2^	9.261 (0.024)	−0.043 (0.000)	34,425	0.420	227.39	60.17 (4.12)
					224.77	
8: *θ*_0_ + *θ*_1_10^−6^ market inc.	3.087 (0.002)	−0.373 (0.001)	40,078	0.476	232.25	59.76 (7.08)
					229.64	
9: *θ*_0_ + *θ*_1_10^−15^market inc.^2^	3.029 (0.002)	−22.056 (0.056)	41,092	0.483	233.05	59.36 (6.97)
					230.44	
10: *θ*_0_ + *θ*_1_(10^−3^ mkt. inc.)^½^	3.675 (0.003)	−0.053 (0.000)	36,301	0.451	229.08	60.94 (7.20)
					226.47	

Note

Standard errors on Gini coefficients are jackknife estimates.

Abbreviations: dispos. inc., disposable income; inc. pc, income per capita.

To illustrate the implications of each model for inequality, the last three columns in Table 2 show the Gini indexes for market income per capita in the survey sample reweighted using the nonresponse‐correction weights ${\hat{P}}_{\mathrm{ij}}^{‐1}$ (refer to Equation 1). These Ginis can be compared to those in Table 1, repeated for convenience in the first row in Table 2, with the “weighted data” Gini viewed as the benchmark.^8^

Individual rows in Table 4 show alternative specifications of $g\left(x_{i},\theta \right)$—logarithmic, linear, polynomial, or square root. Across all models, we find that income has a consistent significant negative effect on the probability of response ($\hat{\theta_{1}}<0$). The corrected Ginis are always higher than the uncorrected ones (by 0.9–8.2 percentage points, and 4.4 on average in the ENIGH‐weighted data). The difference is statistically insignificant at the 5% level but borderline significant at the 10% level in a number of models. Logarithmic functional form appears to outperform linear, square root, or polynomial forms in terms of various model statistics, and market income outperforms gross and disposable incomes, as well as incomes per capita.

The logarithmic and quadratic logarithmic models of market income, models 1 and 7, provide the best fit among the considered models.^9^ They show response probability declining gradually with household income level, slightly more dramatically so in model 7. The wealthiest households in the sample have a predicted response probability as low as 0.182 in model 1, and 0.065 in model 7.

In what follows, Model 1 is used as the model of choice, for its simplicity and conformance with prior studies. Table 3 shows the central implications of this model for the estimated redistributive effects of fiscal policies. Compared to the results in Table 1, the Gini rises by 3.5–4.0 percentage points for all income concepts. The largest correction occurs with taxable income. Whether this large correction could be attributed to the clustering of top taxable incomes or to the absence of nontaxable incomes in the top of the gross income distribution is unclear and should be explored further. Across all income concepts, the upward correction in the Gini tends to increase slightly as one moves from market income to final income, lowering the equalizing effect of cash‐like transfers, direct taxes, and in‐kind transfers found in Table 1.

**TABLE 3:** Income summary statistics for various income concepts, nonresponse corrected weights

	(1)	(2)	(3)	(4)	(5)	(6)	(7)	(8)
	Market income per cap.	Market inc + pensions per cap.	Gross income per cap.	Taxable income per cap.	Net market income per cap.	Disposable income per cap.	Consumable income per cap.	Final income per cap.
99.9th %ile	1,265,377	1,310,623	1,310,623	802,174	1,028,962	1,028,962	977,821	979,925
99th %ile	361,257	370,574	370,574	269,022	324,344	324,344	312,031	315,450
95th %ile	138,716	148,594	148,885	98,607	132,807	133,243	128,139	131,867
90th %ile	90,263	94,975	95,498	64,206	85,414	86,008	82,928	88,227
75th %ile	48,361	49,977	50,557	34,843	46,127	46,623	45,272	49,935
Mean	48,630	50,329	51,365	34,568	45,822	46,858	45,287	49,888
Median	26,802	27,653	28,562	18,689	26,023	26,879	26,209	31,017
25th %ile	15,090	15,739	17,025	9443	15,164	16,424	16,110	20,676
10th %ile	8364	8698	10,590	3005	8626	10,412	10,330	14,603
5th %ile	5015	5179	7467	600	5157	7420	7398	11,686
1st %ile	1823	1866	4033	0	1866	3960	4026	7162
Std. dev.	123,595	124,475	124,313	106,891	114,799	114,655	109,068	108,893
Skewness	20.01	19.59	19.65	26.58	22.84	22.91	22.70	22.75
Kurtosis	588.16	569.91	572.26	946.92	748.75	751.75	740.70	743.40
Sample	73,467	73,467	73,467	73,467	73,467	73,467	73,467	73,467
Top 0.1% inc. share	5.70	5.51	5.40	7.24	5.68	5.56	5.45	4.95
0.1%–1% inc. share	12.31	12.01	11.77	12.43	11.18	10.94	10.83	9.88
1%–5% inc. share	17.09	17.22	16.91	17.33	16.78	16.46	16.32	15.11
5%–10% inc. share	11.38	11.57	11.39	11.47	11.41	11.22	11.18	10.60
Gini (HH‐size and sampling weighted data)	56.41 (1.92)	56.29 (1.86)	54.62 (1.90)	58.31 (2.39)	54.86 (2.01)	53.10 (2.04)	52.65 (2.03)	47.89 (2.02)
Mean log dev.(GE0)	0.591 (0.021)	0.587 (0.021)	0.528 (0.020)	0.680 (0.027)	0.554 (0.021)	0.495 (0.021)	0.486 (0.020)	0.390 (0.018)
Theil index (GE1)	0.761 (0.055)	0.748 (0.053)	0.713 (0.052)	0.840 (0.078)	0.715 (0.058)	0.679 (0.057)	0.667 (0.056)	0.566 (0.051)
Coef. of var. (GE2)	3.230 (0.667)	3.058 (0.624)	2.929 (0.602)	4.781 (1.262)	3.138 (0.755)	2.993 (0.724)	2.900 (0.698)	2.382 (0.583)

Note

Statistics are based on non‐response correction weights, estimated in the logarithmic model of market income (model 1). These statistics exclude 19 household (41 individual) observations with market income of 0. The statistics are still comparable to those in Table 1, which are extremely robust to this exclusion (changing by 0.01 at most). Another 1446 household (2889 individual) 0‐income observations are omitted in computations of the Gini for taxable income. Gini standard errors are jackknife estimates on household‐level data (recognizing that household‐member incomes are copies of one another), accounting for household size except in last row. Ginis and standard errors are multiplied by 100 for clarity of presentation.

In regard to top income shares, the aggregate‐income share of the top 0.1% of households rises by 1.9–2.4 percentage points, representing a 56%–76% gain on the uncorrected share (3.3 percentage points for taxable income, representing an 84% gain). Analogously, the share of the top percentile of households rises by 3.9–5.3 percentage points (33%–37% gain), the share of the top ventile rises by 4.3–5.5 percentage points (15%–17% gain), and the share of the top decile rises by 4.0–4.9 percentage points (10%–11% gain). These increases are similar in magnitude across all income concepts.^10^ Combined with our findings for the Gini, we conclude that the redistributive effects of fiscal instruments are robust to the correction for unit nonresponse, but become slightly less equalizing.

Regarding the effect of individual fiscal instruments, our conclusions from Table 1 remain valid, with three notable differences. One, after correcting for wealthy household nonresponse, contributory pensions are found to have a weak equalizing effect as gauged by the Gini. Two, we now find that households' nontaxable income—the difference between taxable and gross income—has an even more equalizing effect than in Table 1. Such is the case with all top‐income shares and the Gini and particularly with the income share of the top 0.1% and 1% of households. Three, we also find that direct taxes—the difference between gross and disposable income—have an unequalizing effect as gauged by a higher top 0.1% share (and GE(2) index), even though they preserve their equalizing effect on other inequality indexes from Table 1.^11^

**FIGURE 1:**
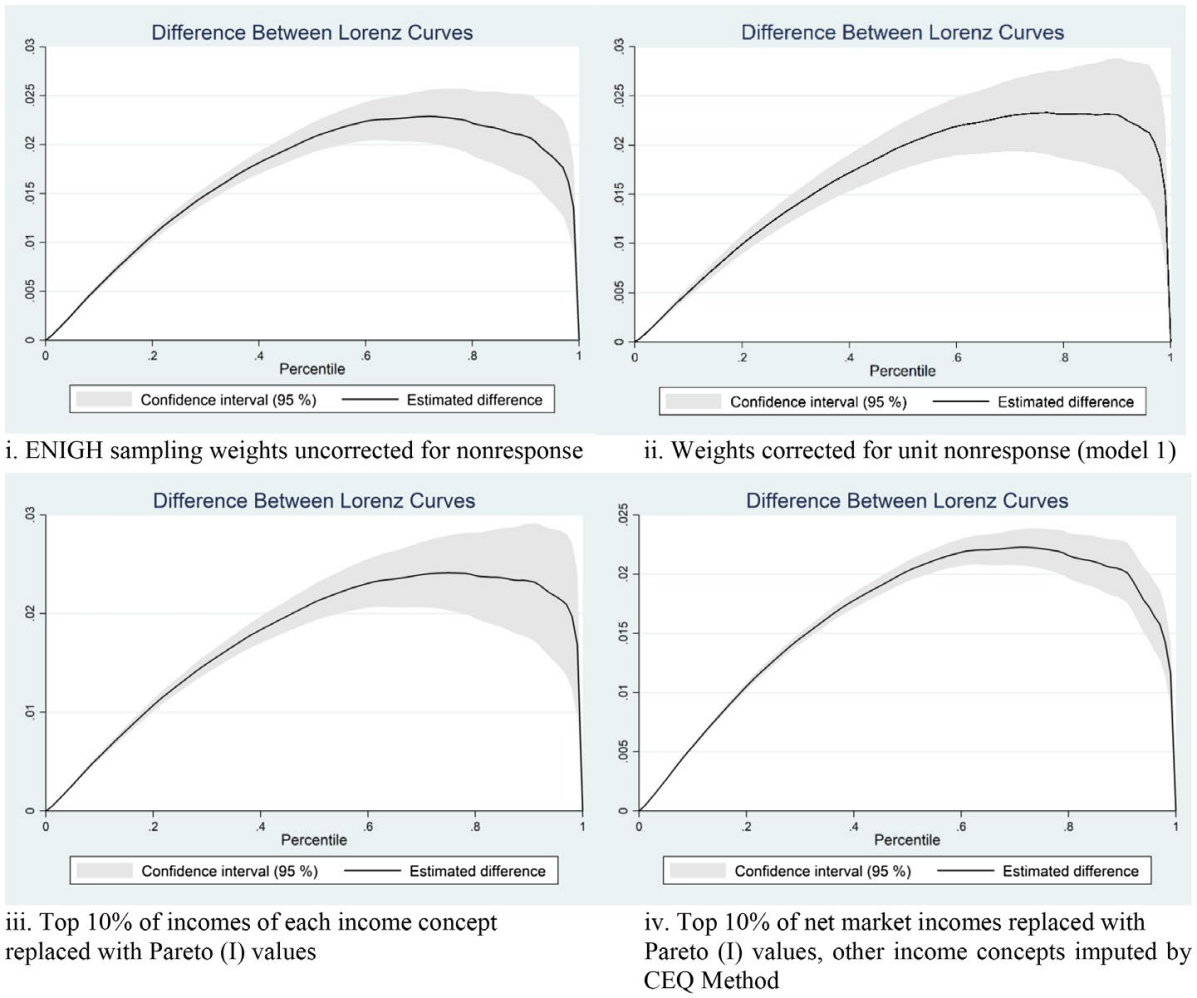
Lorenz curve: market versus disposable income per capita, uncorrected versus corrected income distributions. *Source:* Author's elaboration, based on INEGI (2014), CEQ Data Center (n.d.). Positive values indicate that disposable income Lorenz curve dominates, and shows less inequality than market income Lorenz curve. Distributions account for sampling weights and household size

Figure 1, panels *i* and *ii*, illustrate the cumulative redistributive effects of fiscal instruments between pre‐fiscal (market) income and post‐fiscal (disposable) income, both before and after the correction for wealthy household nonresponse. Disposable income Lorenz curves dominate those for market income, confirming the equalizing effect of fiscal policies taken together. Comparing the nonresponse‐corrected and uncorrected Lorenz curves shows that the estimated equalizing effect increases—the difference between market and disposable Lorenz curves increases—among the top 25% of households.

### Correcting top incomes by replacing with Pareto (type I) estimates

In this section, we embark on correcting the distributions of various income concepts for impurities in their top tails due to misreporting or mis‐recording of some income components. The correction is done by replacing a suspected range of top incomes with smooth estimates from a known statistical distribution, here the Pareto distribution of type I.

Table 4 reports the summary results of this procedure. The first two blocks of rows in Table 4 show the observed distribution of bottom and top incomes in the 2014 ENIGH sample—the Gini coefficients among incomes classified as “bottom” and “top,” under alternative cutoff points. “Top” incomes are those used to fit the Pareto distribution and to be replaced with synthetic values from that distribution.

The third block of rows thus reports the estimated Pareto (type I) coefficients α for individual income concepts and various lower cutoff points. Coefficients α estimated with a lower cutoff point at the 75th, or even the 90th percentile (1.59–1.85 across all but one income concept) are substantially lower than the α estimated with a cutoff at the 95th or 99th percentile (1.84–2.09 across all income concepts). Among the richest 0.1% of households, the α is estimated high, at 2.73–3.61. It indicates that the dispersion of incomes is wide among incomes in the 75th to 95th percentile, narrower among 95–99.9th percentile incomes, and narrower still in the uppermost tail, relative to what would be predicted under a single underlying Pareto distribution.

Correspondingly, inequality measures estimated in the rest of Table 4 show low degrees of inequality among the topmost 0.1% of households, fair inequality among the top 1%–5%, and higher inequality among the top 10, or especially, all top 25%. The inverted Pareto coefficient β is estimated at 1.92–2.19 among the top 1%–5% of households, but rises to 2.18–2.70 among the top 10%–25% (with one exception), and falls to 1.38–1.58 among the top 0.1%. These differences are statistically highly significant, as likelihood ratio tests confirm. The top‐income shares, the parametric Gini, and half the coefficient of variation squared show the analogous patterns of a low degree of income dispersion among the top 1%–5% of households, but high inequality among the top 10%–25% or among the topmost 0.1%.

**TABLE 4:** Replacing top incomes of each income concept with Pareto I distribution: corrected inequality indexes

Cutoff percentile	(1)	(2)	(3)	(4)	(5)	(6)	(7)	(8)
	Market income per cap.	Market inc + pensions per cap.	Gross income per cap.	Taxable income per cap.	Net market income per cap.	Disposable income per cap.	Consumable income per cap.	Final income per cap.
Nonparametric Gini among incomes classified as “bottom”								
Top 25%	30.97 (0.24)	30.86 (0.25)	28.10 (0.22)	33.31 (0.28)	29.92 (0.24)	27.13 (0.21)	26.88 (0.21)	22.68 (0.17)
Top 10%	36.80 (0.23)	36.89 (0.23)	34.61 (0.21)	38.63 (0.25)	35.86 (0.23)	33.52 (0.21)	33.22 (0.21)	28.84 (0.18)
Top 5%	40.67 (0.24)	40.89 (0.24)	38.79 (0.23)	42.27 (0.26)	39.76 (0.24)	37.59 (0.22)	37.25 (0.22)	32.73 (0.20)
Top 1%	46.90 (0.31)	47.24 (0.32)	45.29 (0.31)	48.33 (0.32)	45.93 (0.31)	43.94 (0.30)	43.55 (0.30)	38.81 (0.29)
Top 0.1%	51.22 (0.60)	51.33 (0.57)	49.50 (0.58)	52.74 (0.61)	50.01 (0.55)	48.10 (0.56)	47.68 (0.56)	42.88 (0.55)
Nonparametric Gini among incomes classified as “top”								
Top 25%	37.89 (1.48)	37.67 (1.43)	37.47 (1.42)	38.22 (1.59)	36.59 (1.38)	36.39 (1.37)	36.11 (1.37)	34.24 (1.35)
Top 10%	35.10 (2.03)	34.23 (1.98)	34.09 (1.98)	35.75 (2.21)	33.37 (1.94)	33.23 (1.94)	33.04 (1.94)	31.98 (1.92)
Top 5%	33.70 (2.52)	32.43 (2.49)	32.34 (2.49)	34.54 (2.81)	31.64 (2.49)	31.52 (2.48)	31.40 (2.48)	30.82 (2.47)
Top 1%	30.55 (3.70)	30.14 (3.73)	30.15 (3.72)	31.16 (5.02)	29.10 (4.33)	29.09 (4.32)	29.08 (4.28)	28.93 (4.26)
Top 0.1%	21.66 (11.82)	20.93 (11.91)	20.93 (11.89)	30.74 (16.65)	26.85 (15.20)	26.86 (15.17)	26.46 (15.26)	26.43 (15.25)
Pareto (type I) coefficient among “top” incomes								
Top 25%	1.62 (0.03)	1.59 (0.03)	1.60 (0.03)	1.59 (0.03)	1.65 (0.03)	1.66 (0.03)	1.67 (0.03)	1.78 (0.03)
Top 10%	1.76 (0.05)	1.77 (0.05)	1.79 (0.06)	1.77 (0.06)	1.82 (0.05)	1.83 (0.06)	1.85 (0.06)	1.94 (0.06)
Top 5%	1.84 (0.09)	1.89 (0.09)	1.91 (0.09)	1.84 (0.09)	1.93 (0.09)	1.94 (0.09)	1.94 (0.09)	2.00 (0.09)
Top 1%	1.99 (0.22)	2.03 (0.22)	2.06 (0.22)	1.97 (0.20)	2.04 (0.20)	2.04 (0.20)	2.06 (0.21)	2.09 (0.21)
Top 0.1%	3.61 (1.22)	2.73 (1.05)	2.73 (1.05)	3.42 (2.23)	3.33 (2.04)	3.32 (2.04)	3.37 (2.02)	3.38 (2.07)
Parametric Gini among “top” incomes								
Top 25%	44.48	45.78	45.37	45.92	43.53	43.14	42.70	39.17
Top 10%	39.76	39.37	38.70	39.28	37.94	37.65	37.02	34.73
Top 5%	37.22	35.85	35.35	37.40	34.95	34.74	34.86	33.44
Top 1%	33.65	33.44	32.95	33.97	32.72	32.80	32.20	31.56
Top 0.1%	19.21	20.80	20.84	16.53	16.60	16.63	16.73	16.47
Income share (%) among incomes classified as “top”								
Top 25%	65.57	66.24	65.03	67.96	64.59	63.31	62.90	58.64
Top 10%	43.72	43.90	42.85	44.84	42.40	41.45	40.93	37.36
Top 5%	31.37	31.13	30.40	32.40	30.00	29.27	29.03	26.29
Top 1%	14.09	13.60	13.32	14.77	12.97	12.67	12.46	11.22
Top 0.1%	3.33	3.25	3.17	3.16	2.64	2.57	2.52	2.26
Semiparametric Gini (combining nonparametric “bottom” and parametric “top” Gini)								
Top 25%	55.86 (1.37)	56.64 (1.46)	54.77 (1.51)	58.77 (2.18)	54.56 (1.96)	52.62 (2.06)	52.10 (2.10)	46.42 (1.37)
Top 10%	54.10 (1.22)	54.25 (1.22)	52.32 (1.14)	55.78 (1.30)	52.60 (1.27)	50.67 (1.33)	50.11 (1.31)	44.92 (1.30)
Top 5%	53.47 (1.03)	53.45 (1.00)	51.58 (1.00)	55.15 (1.28)	51.95 (1.17)	50.04 (1.08)	49.65 (1.17)	44.65 (1.17)
Top 1%	53.03 (1.00)	53.08 (0.90)	51.24 (0.94)	54.60 (1.27)	51.60 (1.02)	49.71 (1.14)	49.24 (1.00)	44.37 (1.01)
Top 0.1%	52.71 (0.76)	52.78 (0.72)	50.97 (0.74)	54.11 (0.77)	51.21 (0.71)	49.31 (0.71)	48.88 (0.70)	44.05 (0.69)
Uncorrected	52.75 (0.82)	52.79 (0.79)	50.99 (0.80)	54.31 (0.86)	51.33 (0.76)	49.43 (0.77)	49.00 (0.77)	44.17 (0.76)
Gini correction								
Top 25%	+3.11	+3.85	+3.78	+4.46	+3.23	+3.19	+3.10	+2.25
Top 10%	+1.35	+1.46	+1.33	+1.47	+1.27	+1.24	+1.11	+0.75
Top 5%	+0.72	+0.66	+0.59	+0.84	+0.62	+0.61	+0.65	+0.48
Top 1%	+0.28	+0.29	+0.25	+0.29	+0.27	+0.28	+0.24	+0.20
Top 0.1%	−0.04	−0.01	−0.02	−0.20	−0.12	−0.12	−0.12	−0.12

Note

Nonparametric (semiparametric) Gini standard errors are jackknife (bootstrap) estimates. Ginis and standard errors are multiplied by 100 for clarity of presentation.

Using the above parametric estimates among the top tail of incomes, we reestimate inequality under the entire income distribution. The bottom rows of Table 4 report the results. Compared to the original uncorrected Ginis in Table 1, the Ginis corrected for suspected top‐income mismeasurements are systematically higher, by 0.2–4.5 percentage points across all income concepts and all choices regarding cutoff points (except when only the top 0.1% of observations are replaced). These are systematic and sizeable corrections.^12^ ^,^ ^13^

The upward corrections to the Gini are highest for taxable income, just as we saw with the correction for unit nonresponse. In tandem, these findings suggest that the distribution of taxable income may be less smooth, and is affected when the nonresponse weights or the parametric replacing are applied to its top values. Unlike the corrections for unit nonresponse, the corrections in Table 4 appear to decline slightly as we move from market income to final income. The effect of fiscal policies is thus estimated to be more equalizing. Cash‐like transfers are estimated to reduce the Gini by 1.8–1.9 points, and direct taxes by 1.5–2.2 (see Figure 1, panel *iii*). In‐kind transfers reduce the Gini by 4.9–5.7 points, mean 5.6, across the different cutoff points. Contributory pensions are found to be unequalizing, as gauged by the rising Gini as well as the top 10 and top 25% income share, but equalizing at the complete top, as gauged by the falling top 5%, 1% or 0.1% shares.

As in Tables 1 and 3, nontaxable income has an equalizing effect in terms of most inequality indicators. Yet here, the effect becomes neutral or even slightly unequalizing at the top tail, gauged by the income share of the top 0.1% of households, and the equalizing effect becomes stronger lower down in the income distribution, for a 4 percentage point decrease in the Gini (compared to 3.3 in Tables 1 and 3).

### Correcting top net‐market incomes through Pareto (type I) replacing and using CEQ method

One modality of the Pareto replacing method is to replace the top tail for the income concept thought to be most susceptible to mismeasurement and then recalculate the rest of the derived income concepts using the CEQ Method. Net market income per capita, taken directly from survey questionnaires and being the starting concept in the CEQ methodology from which other income concepts are imputed, is the natural choice for this exercise.

The model used here is the same as in the previous section for net‐market income per capita (Table 4, column 5). For the main model specification, we choose the lower cutoff at the 90th percentile. Hence, reported incomes above 80,362 Mexican pesos are viewed as potentially misreported and are replaced with random draws from the estimated Pareto distribution. This model specification is expected to produce intermediate estimates of the Pareto coefficient and intermediate corrections of inequality compared to the ranges presented in Table 4.

Table 5 reports the corrected distributions of all income concepts. By design, the Gini coefficient and the top 10%‐income share obtained for net‐market income per capita is nearly identical to that in Table 4 (52.68 vs. 52.60 for the Gini, 42.49 vs. 42.40 for the top 10% share), the minute differences being due to random drawing from the Pareto distribution. Across income concepts and the intervening fiscal instruments, our findings are similar to those in Table 4. Pensions have a neutral effect, slightly equalizing among the topmost one percent of incomes, and slightly unequalizing among lower incomes, leading to no change in the Gini. The adding of nontaxable incomes has an equalizing effect of a similar magnitude as in Table 4, of 3.8 points of the Gini. The equalizing effect of direct taxes is estimated to be approximately 1.4 points of the Gini, and that of in‐kind programs, approximately 4.9 points, slightly lower than in Tables 3 and 4. Figure 1, panel *iv* illustrates the cumulative redistributive effects of fiscal instruments, using the entire Lorenz curve.

**TABLE 5:** Income summary statistics: replacing top net‐market incomes with Pareto I estimates, and imputing other income concepts by CEQ method

	(1)	(2)	(3)	(4)	(5)	(6)	(7)	(8)
	Market income per cap.	Market inc + pensions per cap.	Gross income per cap.	Taxable income per cap.	Net market income per cap.	Disposable income per cap.	Consumable income per cap.	Final income per cap.
99.9th %ile	1,586,003	1,585,680	1,585,680	1,261,436	1,377,565	1,377,565	1,320,081	1,322,184
99th %ile	317,481	322,046	322,046	232,907	286,984	286,984	277,402	281,135
95th %ile	123,626	130,824	131,988	87,958	117,226	118,115	113,030	117,428
90th %ile	83,668	87,845	88,601	60,407	80,362	80,680	78,196	82,721
75th %ile	46,473	48,042	48,594	32,975	44,590	45,144	43,769	48,405
Mean	44,321	46,055	47,127	31,397	42,187	43,260	41,848	46,454
Median	25,983	26,941	27,747	18,031	25,443	26,286	25,659	30,340
25th %ile	14,675	15,298	16,642	9237	14,754	16,045	15,782	20,377
10th %ile	8113	8454	10,353	2833	8354	10,226	10,112	14,357
5th %ile	4820	5006	7323	525	4950	7255	7207	11,480
1st %ile	1751	1796	3944	0	1796	3903	3917	7098
Std. dev.	98,330	99,735	99,571	80,198	89,632	89,485	85,228	85,071
Skewness	17.76	17.27	17.34	22.34	18.72	18.80	18.60	18.63
Kurtosis	505.22	482.50	485.23	817.68	591.49	594.94	582.52	584.64
Sample	73,508	73,508	73,508	73,508	73,508	73,508	73,508	73,508
Top 0.1% inc. share	5.00	4.85	4.74	5.85	4.72	4.61	4.53	4.09
0.1%–1% inc. share	11.10	10.86	10.62	11.87	10.58	10.33	10.19	9.23
1%–5% inc. share	16.19	16.45	16.12	16.36	15.96	15.63	15.50	14.29
5%–10% inc. share	11.34	11.42	11.24	11.50	11.23	11.02	10.99	10.40
Gini (HH‐size and sampling weighted data)	54.00	54.00	52.21	55.94	52.68	50.80	50.35	45.50
	(1.04)	(1.00)	(1.02)	(1.13)	(0.99)	(1.01)	(1.00)	(0.99)
Mean log dev.(GE0)	0.541	0.541	0.481	0.630	0.511	0.452	0.443	0.351
	(0.012)	(0.012)	(0.011)	(0.014)	(0.011)	(0.011)	(0.011)	(0.009)
Theil index (GE1)	0.672	0.664	0.629	0.737	0.634	0.598	0.586	0.490
	(0.028)	(0.027)	(0.027)	(0.033)	(0.026)	(0.026)	(0.025)	(0.023)
Coef. of var. (GE2)	2.461	2.345	2.232	3.262	2.257	2.139	2.074	1.677
	(0.253)	(0.240)	(0.230)	(0.396)	(0.247)	(0.236)	(0.227)	(0.187)

Note

Statistics are based on non‐response correction weights estimated in the logarithmic model of market income (model 1). These statistics exclude 19 household (41 individual) observations with market income of 0. The statistics are still comparable to those in Table 1, which are extremely robust to this exclusion (changing by 0.01 at most). Another 1446 household (2889 individual) 0‐income observations are omitted in computations of the Gini for taxable income. Gini standard errors are jackknife estimates on household‐level data (recognizing that household‐member incomes are copies of one another), accounting for household size except in last row. Ginis and standard errors are multiplied by 100 for clarity of presentation.

### The main results for the redistributive effects of fiscal instruments

Tables 6 and 7 summarize the main results of the corrections implemented in this study, including the ranges of inequality estimates and of the estimated redistributive effects of fiscal programs. Table 6 shows the full ranges (mean, median, and the extreme) of the Ginis for each income concept estimated under various behavioral specifications of the reweighting model in Table 2, and various cutoffs for the Pareto replacing in Table 4. For convenience, the table reports the estimated changes in the uncorrected Ginis (in bold). The reweighting method is shown to correct the Gini upward by 4.3–5.2 percentage points on average (median 4.1–4.8), and by as much as 10.1. The replacing method corrects the Ginis by 0.7–1.4 percentage points on average (median 0.5–0.9), and by as much as 4.5. Reassuringly, the two modalities of the replacing method lead to similar magnitudes of corrections for all income concepts.

**TABLE 6:** Summary results of correction methods: corrected Ginis for all income concepts

	(1)	(2)	(3)	(4)	(5)	(6)	(7)	(8)
	Market income per cap.	Market inc + pensions per cap.	Gross income per cap.	Taxable income per cap.	Net market income per cap.	Disposable income per cap.	Consumable income per cap.	Final income per cap.
Uncorrected	52.8	52.8	51.0	54.3	51.3	49.4	49.0	44.2
Correction by reweighting (models in Table 2)								
Minimum	53.7 + **0.9**	53.7 + **0.9**	51.9 + **0.9**	55.2 + **0.9**	52.2 + **0.9**	50.4 + **1.0**	49.9 + **0.9**	45.2 + **1.0**
Mean	57.2 + **4.4**	57.1 + **4.3**	55.4 + **4.4**	59.6 + **5.2**	55.9 + **4.5**	54.1 + **4.7**	53.6 + **4.6**	48.9 + **4.7**
Median	57.0 + **4.3**	56.9 + **4.1**	55.3 + **4.3**	59.1 + **4.8**	55.6 + **4.2**	53.8 + **4.4**	53.4 + **4.4**	48.6 + **4.4**
Maximum	60.9 + **8.2**	60.7 + **7.9**	59.1 + **8.1**	64.4 + **10.1**	60.0 + **8.6**	58.2 + **8.8**	57.7 + **8.7**	53.0 + **8.8**
Correction by Pareto (type I) replacing of own income concept								
Minimum	52.7 + **0.0**	52.8 + **0.0**	51.0 + **0.0**	54.1 **− 0.2**	51.2 **− 0.1**	49.3 **− 0.1**	48.9 **− 0.1**	44.1 **− 0.1**
Mean	53.8 + **1.1**	54.0 + **1.3**	52.2 + **1.2**	55.7 + **1.4**	52.4 + **1.1**	50.5 + **1.0**	50.0 + **1.0**	44.9 + **0.7**
Median	53.5 + **0.7**	53.5 + **0.7**	51.6 + **0.6**	55.2 + **0.8**	52.0 + **0.6**	50.0 + **0.6**	49.7 + **0.6**	44.7 + **0.5**
Maximum	55.9 + **3.1**	56.6 + **3.9**	54.8 + **3.8**	58.8 + **4.5**	54.6 + **3.2**	52.6 + **3.2**	52.1 + **3.1**	46.4 + **2.3**
Correction by Pareto (type I) replacing of market income + CEQ Method								
Minimum	52.6 **− 0.1**	52.7 **− 0.1**	50.9 + **0.0**	54.1 **− 0.2**	51.2 **− 0.1**	49.3 **− 0.1**	48.9 **− 0.1**	44.0 **− 0.1**
Mean	53.8 + **1.1**	53.8 + **1.0**	52.0 + **1.0**	55.7 + **1.4**	52.5 + **1.1**	50.6 + **1.2**	50.1 + **1.1**	45.3 + **1.1**
Median	53.4 + **0.7**	53.5 + **0.7**	51.7 + **0.7**	55.2 + **0.9**	52.1 + **0.8**	50.2 + **0.8**	49.8 + **0.8**	44.9 + **0.7**
Maximum	55.9 + **3.1**	55.7 + **2.9**	53.9 + **3.0**	58.4 + **4.1**	54.6 + **3.3**	52.7 + **3.3**	52.3 + **3.3**	47.4 + **3.2**

Note

Pc.pt. differences from uncorrected Ginis in bold. These Ginis and differences in them arise from “Gini (HH‐size and sampling weighted data)” in Tables 1 and 3, and “Semiparametric Gini” in Table 4. Ginis and percentage point changes are multiplied by 100 for clarity of presentation.

**TABLE 7:** Redistributive effects of fiscal tools: high/center/low estimates of effects on inequality

	Market income inequality	+ Net contributory pensions	+ Cash‐like transfers	+ Nontaxable income	− Direct taxes	− Indirect taxes and subsidies	+ Net in‐kind programs	Final income inequality
		Market → market + pensions	Market + pensions → gross	Taxable → gross	Gross → disposable^a^	Disposable → consumable	Consumable → final	
***Gini coefficient: pc.pt. change***								
Uncorrected	52.75	+0.04	−1.80	−3.32	−1.56	−0.43	−4.83	44.17
Income distrib. corrected for nonresponse by reweighting								
High	60.17	+0.00	−1.74	−3.32	−1.58	−0.44	−4.78	51.89
Center	56.41	−0.12	−1.67	−3.69	−1.52	−0.45	−4.76	47.89
Low	53.78	−0.24	−1.59	−4.42	−1.27	−0.47	−4.70	45.24
Each income concept corrected for top income mismeasurement by replacing								
High	55.86	+0.05	−1.84	−3.36	−1.53	−0.47	−4.87	46.42
Center	54.10	+0.15	−1.93	−3.46	−1.65	−0.56	−5.19	44.92
Low	53.03	+0.78	−1.87	−4.00	−2.15	−0.52	−5.68	44.37
Market income corrected for top income biases by replacing + CEQ Method								
High	55.85	+0.00	−1.79	−3.73	−1.41	−0.45	−4.85	47.36
Center	54.00	+0.04	−1.80	−3.42	−1.51	−0.44	−4.84	45.50
Low	53.15	−0.14	−1.77	−4.50	−1.21	−0.48	−4.89	44.61
***Top 10 percent income share: pc.pt. change***								
Uncorrected	42.10	+0.04	−0.85	−2.28	−1.29	−0.35	−3.13	36.52
Income distrib. corrected for nonresponse by reweighting								
High	51.12	−0.03	−0.83	−2.39	−1.33	−0.37	−3.12	45.08
Center	46.48	−0.17	−0.84	−3.00	−1.29	−0.40	−3.24	40.54
Low	43.30	−0.33	−0.84	−4.08	−1.05	−0.42	−3.40	37.62
Each income concept corrected for top income mismeasurement by replacing								
High	46.57	+0.28	−0.92	−2.19	−0.85	−0.48	−2.61	39.80
Center	43.72	+0.18	−1.05	−1.99	−1.40	−0.52	−3.57	37.36
Low	40.28	+1.22	−0.94	−2.03	−2.18	−0.43	−4.44	35.70
Market income corrected for top income biases by replacing + CEQ Method								
High	46.17	+0.04	−0.86	−2.41	−1.23	−0.37	−3.15	40.48
Center	43.63	−0.05	−0.86	−2.86	−1.13	−0.38	−3.20	38.01
Low	42.60	−0.13	−0.88	−3.80	−0.89	−0.43	−3.36	37.03
***Top 1 percent income share: pc.pt. change***								
Uncorrected	13.59	−0.38	−0.30	−1.43	−0.69	−0.15	−1.17	10.90
Income distrib. corrected for nonresponse by reweighting								
High	23.70	−0.39	−0.30	−1.56	−0.73	−0.18	−1.20	20.19
Center	18.01	−0.49	−0.35	−2.50	−0.67	−0.22	−1.45	14.83
Low	14.56	−0.65	−0.42	−4.27	−0.34	−0.28	−1.82	11.76
Each income concept corrected for top income mismeasurement by replacing								
High	17.48	−0.49	−0.28	−1.45	−0.65	−0.21	−1.24	13.08
Center	15.89	−0.60	−0.37	−1.46	−0.98	−0.25	−1.75	11.95
Low	14.09	−0.05	−0.33	−1.64	−1.50	−0.22	−2.30	11.22
Market income corrected for top income biases by replacing + CEQ Method								
High	20.42	−0.39	−0.35	−2.36	−0.42	−0.22	−1.40	17.37
Center	16.10	−0.36	−0.32	−1.64	−0.61	−0.17	−1.23	13.32
Low	14.31	−0.47	−0.43	−4.04	−0.05	−0.31	−1.79	11.62

Note

These Ginis are comparable to “Gini (HH‐size and sampling weighted data)” in Tables 1 and 3, and “Semiparametric Gini” in Table 4. Ginis and percentage point changes are multiplied by 100 for clarity of presentation.

^a^ Alternatively, it can be obtained as “market + pensions → net market” for estimates within 0.1 pc.pt. of those above.

Table 7 offers a slightly different perspective on the estimated redistributive effects of fiscal policies. Instead of showing the estimates of inequality, percentage point differences across income concepts are shown. Moreover, instead of showing the full range of estimates including outliers, three parametric forms of the correction methods are chosen as representing the low, central, and high points of reasonable specifications. Under the reweighting approach, the per‐capita model 4, the household market income model 1, and the quadratic model 7—those that show good theoretical justification, are consistent with one another, and have empirical fit—are used as the low, central, and high specifications. Of course, the redistributive effects estimated under these low‐to‐high specifications may not be related monotonically to one another and may not be ranked from low to high. Under the replacing approach, lower cutoffs at the 75th, 90th, and 99th percentiles—again, models showcasing some theoretical justification and adequate empirical fit—are used as the low, central, and high specifications.

For all three specifications under each correction approach, Table 7 reports the percentage point changes to the Ginis and in the top‐income shares attributable to specific fiscal instruments, that is, the differences in inequality indices between the pairs of adjacent income concepts. Table 7 confirms that pensions have a negligible effect on inequality, and that in fact the effect varies across quantiles of the income distribution (as seen by the changes in the Gini vs. the top income shares). Cash‐like transfers have a strong equalizing effect of 1.6–1.9 percentage points of the Gini. The effect of nontaxable income is stronger still, at 3.3–4.5 percentage points, where the corrected estimates are universally larger than or as large as the uncorrected figure (3.3 percentage points). Progressive direct taxes account for another 1.2–2.2 percentage point drop in the Gini. Indirect taxes and subsidies have a weak equalizing effect of 0.4–0.6 points of the Gini, but again this is universally larger than the uncorrected figure (0.4 percentage points). In‐kind transfers have a strong equalizing effect of 4.7–5.7 points of the Gini, again typically larger than the uncorrected effects (4.8 percentage points).^14^

## SUMMARY AND DISCUSSION

This study has evaluated the redistributive effects of various fiscal policy instruments in Mexico, using the 2010–2014 ENIGH (CEQ Data Center, n.d.) surveys, and applying two specific corrections for potential top‐income measurement problems. We have first reweighted the survey sample to correct the income distribution for selective nonresponse by wealthy households, and then we have replaced potentially mismeasured top incomes with synthetic values from a smooth parametric distribution. By comparing the uncorrected measures of inequality and degrees of redistributive fiscal effects with the two alternative sets of corrected figures, we have evaluated the robustness of the uncorrected figures and provided improved estimates.

The key result of the study is that pensions in Mexico are confirmed to be inequality‐neutral, whereas in‐kind transfers, cash‐like transfers and direct taxes have strong equalizing effects, of 4.7–5.7, 1.6–1.9, and 1.2–2.2 points of the Gini, respectively. Indirect taxes and subsidies are equalizing only weakly, by 0.4–0.6 points of the Gini.

The new estimates should not be considered as accurate or unbiased, since they correct for a single source of imprecision at a time. Yet, because the corrected estimates were obtained using established and transparent methods, and using rather conservative modeling specifications, they can be viewed as improved baseline estimates that can be evaluated for other biases. Both the uncorrected and corrected estimates have large standard errors, suggesting that sampling error tends to dominate estimation error, but the differences in estimates are quite consistent, and significant in a number of cases.

Tables 6 and 7 summarize the main results of this article for the estimates of inequality and fiscal redistributive effects. Across the board, corrections to the Gini coefficients and top‐income shares are positive, suggesting that the uncorrected statistics suffer from a downward bias, and the corrected estimates of the redistributive effects are qualitatively similar to the uncorrected effects, which helps to validate our methods. The corrected estimates of the redistributive effects differ quantitatively from the uncorrected ones in a number of cases, and the differences are systematic.

As we move from pre‐fiscal toward post‐fiscal incomes, the corrections to inequality estimates under the reweighting method increase somewhat, while the corrections under replacing tend to fall or stagnate. This suggests that measurement problems differ under the different income concepts, or that households' pre‐fiscal and post‐fiscal incomes are associated in nonobvious ways. Correcting for unit nonresponse through monotonic reweighting of top observations reduces the equalizing redistributive effect of fiscal programs. It may be due to limited progressivity of taxes and transfers, or to fiscal loopholes among households with top taxable incomes, whose weight is corrected the most.

Income measurement issues as evidenced by comparisons to smooth Pareto distributions appear to affect most seriously the distribution of pre‐fiscal incomes, for which the estimated biases are larger. Taxable income is the income concept most heavily affected by both unit nonresponse and mismeasurement. Whether the large estimated biases are due to unreported taxable earnings or to some clustering of top taxable incomes is unclear and should be explored further. Nontaxable income is shown to be even more equalizing after correcting gross income than in the uncorrected distribution, suggesting that nontaxable incomes are not very prevalent in the top tail of the gross income distribution, where the bulk of the upward corrections—by reweighting or replacing—takes place.

The corrections for possible misreporting of top incomes, by Pareto replacing, can be compared to the corrections for unit nonresponse by reweighting, to judge the relative gravity of these two distinct problems (as Tables 6 and 7 show). Interestingly, the mean, median, and maximum corrections to the Gini are substantially higher under the reweighting method.

Unit nonresponse leads to substantial underestimation of mean incomes and measures of inequality. The Gini coefficient of market income per capita is found to be biased downward by up to 8.2 percentage points, and typically by 4.3 points across all estimations, and across all three survey waves. The Gini for final income is biased downward by up to 8.8 points, and typically by 4.4 points. By contrast, the suspected tainting of the distribution of top incomes by income mismeasurement biases the Gini of market income per capita by up to an estimated 3.1 points, and typically by only 0.7 points, and these biases fall to 2.3 and 0.5 points for final income per capita.

The study confirms that unit nonresponse is a systematic and nonnegligible problem in the Mexican ENIGH survey. Along with other top‐income measurement challenges, unit nonresponse rate retains its magnitude across the 2010–2014 versions of the ENIGH, and further grows in 2016 and 2018. Moreover, household nonresponse becomes more positively selected over time, causing more serious measurement biases. The corrected estimates of the Gini coefficient are found to be stagnant during 2010–2014, corroborating the findings by Campos‐Vázquez and Lustig (2017), and Del Castillo Negrete Rovira (2017), and contradicting the widely cited narrative of a falling inequality. Analysts and policymakers relying on ENIGH would be wise to take note.

## Supporting information

Supplementary Material

## ENDNOTES

^1^
Another branch of literature has proposed deriving the counterfactual distribution from outside the survey. This branch used parametric smoothing to combine within‐survey incomes (micro data or group data) with out‐of‐survey information such as top income shares or national accounts data (Anand & Segal, 2017; Lakner & Milanovic, 2013; Novokmet et al., 2017; Piketty, Yang & Zucman, 2016). Our study uses only data from within the survey.

^2^
It includes the two‐parameter generalized Pareto (type II) distribution and the four‐parameter generalized beta distribution (Hlasny & Verme, 2018a; 2018c; Jenkins, 2017). The empirical fit of other candidate distributions—including the lognormal, Fisk, Singh‐Maddala and Dagum distributions (they limiting cases of the generalized beta‐II) has been questioned (Brachmann, Stich & Trede, 1996; Brzezinski, 2013; Butler & McDonald, 1989; Jenkins, 2007, 2009). On the other hand, Jenkins (2007, 2009) and Jenkins et al., (2011) make the case for using the generalized beta distribution (type II) as providing more flexibility and superior fit at the top end. A general review of evidence is provided by Hlasny (2020a). In this study, we assess only the Pareto (type I) distribution, because of its lower requirements on data size.

^3^
Led by Nora Lustig since 2008, the Commitment to Equity (CEQ) project is an initiative of the Center for Inter‐American Policy and Research (CIPR) and the Department of Economics, Tulane University, the Center for Global Development and the Inter‐American Dialogue. The CEQ project is housed in the Commitment to Equity Institute at Tulane. The Bill & Melinda Gates Foundation has provided major funding for the preparation of the CEQ Handbook 2016 and updates. For more details, see CEQ Institute (2020), Lustig et al. (2006, 2007), and Lustig (2018, 2020).

^4^
Faulkner (1996a, 1996b) has recently provided a positive evaluation of survey incomes in the ENIGH but has found that incomes fall short of consumption estimates, and has proposed that the income distribution's high skew calls for the use of modified inequality indexes, such as G2 (Gini × mean/median).

^5^
Figures with analogous findings were obtained for the 50th–99th percentiles of incomes and for maximum incomes in each state, available on request. These statistics are potentially less sensitive to top‐income nonresponse than mean incomes. Additional information on the ENIGH, including nonresponse rates, are available on request in an Appendix.

^6^
The difference in data sources presents some compatibility challenges. In the 2010 survey, the only income concepts available are disposable household income and disposable household income net of after‐tax private and social security transfers (in LIS nomenclature, which differs from CEQ), because all incomes are surveyed net of taxes, social contributions, and other deductions such as union fees. See Lis Data Center (n.d.).

^7^
Besides the benchmark Gini on the fully weighted income distribution, we have assessed the Ginis on an unweighted income distribution using post‐stratification weights, and on an entirely unweighted (=equal household weights) distribution. They are available on request. The results in Table 1 can be contrasted with those using prior surveys. In 2012, the top decile of households accounted for 40.9% of market income, and 35.8% of final income; the top ventile accounted for 28.4% and 24.3%; and the top percentile accounted for 11.3% and 9.1%, respectively. The Gini was 52.1 for market income, 44.0 for final income. In 2010, for disposable income net of private and social security transfers, the top decile accounted for 38.8%, the top ventile, for 26.7%; and the top percentile, for 10.8%. The Gini was 49.7. For disposable income inclusive of transfers, the top decile accounted for 36.5%, the top ventile, for 24.9%; the top percentile, for 9.8%, and the Gini was 47.1. The degree of dispersion among top disposable incomes, as well as general income inequality, thus appears to have risen in Mexico during both periods 2010–2012 and 2012–2014.

^8^
Because survey weights in ENIGH already provide limited correction for nonresponse, it is a standard practice to report Ginis derived under composite weights (${\hat{P}}_{\mathrm{ij}}^{‐1}\times s_{j}^{‐1}\times HHsize$, “weighted data”); Ginis using only the nonresponse‐correction weights (${\hat{P}}_{\mathrm{ij}}^{‐1}\times HHsize$); or Ginis on an unweighted sample where each household is given equal weight and only nonresponse correction is applied (${\hat{P}}_{\mathrm{ij}}^{‐1}$). These alternative Ginis are available on request.

^9^
This analysis was also extended by considering multivariate functions of income and additional characteristics of household heads—age, age squared, gender, household size, and binary indicators of urban/rural residence, current attendance of post‐secondary schools, and formal employment status. Simple univariate functions of income in Table 4 have better efficiency properties with respect to various model statistics. They yield greater significance of coefficients, lower value of the minimization objective function, lower values of the information criteria, and lower standard errors on the Ginis. These findings are supported in the ENIGH 2012 (CEQ Data Center, n.d.) data. On the other hand, in the ENIGH 2010 (LIS Data Center, n.d.), income is consistently estimated to have a positive effect on response probability ($\hat{\theta_{1}}<0$), and the corrected Ginis are typically lower than the uncorrected ones.

^10^
The corrected Lorenz curves are shown to be strictly dominated and show more inequality than the uncorrected curves across different parts of the income distribution. In ENIGH 2012 (CEQ Data Center, n.d.), we find much lower differences in Lorenz curves. In ENIGH 2010 (LIS Data Center, n.d.), interestingly, the nonresponse‐corrected Lorenz curve dominates the uncorrected curve in the top third of the income distribution, implying that the correction lowers the estimated inequality at the top.

^11^
The same analysis performed on the 2012 version of the ENIGH reveals essentially the same qualitative patterns, but of much smaller magnitudes, due to a weaker link between incomes and response probability. Across all considered models, the Ginis increase by 0.3–6.7 pc.pt. Using the preferred model (same as in 2014), the Ginis increase by 0.4 pc.pt. across all income concepts, and the top income shares increase by 0.2–0.3 pc.pt. for the top 1% share, and by 0.4–0.5 for the top 10% share. Because of the weak corrections for nonresponse, pensions are found to have an unequalizing effect, as measured by the top 5% and 10% income share as well as the Gini, both before and after the correction. In the 2010 round, the only two available income concepts allow us to comment only on the redistributive effects of cash‐like after‐tax transfers. The correction for nonresponse leads to non‐positive changes in the Gini and the top income shares in most estimated models. The Gini increases by 0.03–0.15 pc.pt., the top percentile share increases by 0.4pc.pt., and the top decile share increases by 0.6–0.7pc.pt., all very small effects. The redistributive effect of cash‐like after‐tax transfers is equalizing, at 2.6 points of the Gini, which increases to 2.7 points after the correction for unit nonresponse.

^12^
In the 2012 version of the ENIGH, a different pattern emerges. Among the richest 0.1% of households, the coefficient α becomes low again (1.52–1.76), due in part to the outlier in the sample. The inverted Pareto coefficient β is estimated at 1.57–1.90 among the top 1%–5% of households, but rises to 2.12–2.92 among the top 10%–25% or among the top 0.1%. The corrected semiparametric Ginis for the entire income distribution are systematically higher than the uncorrected Ginis (with a single exception), by 0.0–5.1 pc.pt. In the 2010 version, the estimated Pareto coefficient rises gradually as the lower cutoff is increased, and the corresponding measures of inequality fall. The corrected Gini for disposable income net of transfers is 4.0 pc.pt. higher than the uncorrected Gini when all of the top 25% of incomes are replaced, but by as little as 0.2 pc.pt. when only the top 1% are replaced (2.9 and 0.1 pc.pt., respectively, for disposable income). In light of the sensitivity of the α parameter to the cutoff points, across all survey rounds and various income concepts, the Pareto (I) approximation may not be adequate when we include lower incomes such as those in the 75–90th percentile.

^13^
As a robustness test, we replace top incomes with estimates from the generalized Pareto (type II) distribution. Its cumulative density function is $F\left(x\right)=1‐{\left[1+x/\sigma \right]}^{‐\xi }$ where *ξ* is a shape parameter and *σ* is a scale parameter (e.g., Arnold, 2008). The Gini under the parametric distribution can be computed as: $Gini=1‐\left[L+2\sigma \xi^{‐2}G\left(2\xi^{‐1}‐1\right)G\left(2\right)G\left(2\xi^{‐1}+1\right)\right]/\left[L+\sigma \xi^{‐2}G\left(\xi^{‐1}‐1\right)G\left(2\right)G\left(\xi^{‐1}+1\right)\right]$ where L is the lower cutoff income, and G is the gamma function. Here, the shape coefficient does not change much across different income concepts and different delineations of top incomes, except for the extreme cutoff at the 99.9th percentile. Using the 99.9th percentile cutoff, the shape parameter is estimated much lower for market income + pensions and gross income, suggesting that a simple exponential distribution may describe the upper‐most dispersion for these income concepts adequately. For the rest of income concepts, the shape parameter among the top 0.1% of incomes is estimated higher. The estimated generalized Pareto (type II) distribution yields higher shape coefficients, lower inverted Pareto coefficients, and lower Ginis than the Pareto I specification. Because the Pareto I specification is viewed as less sensitive to individual income values, we use Pareto I as the main model.

^14^
Qualitative results for 2012 are very similar to those in Tables 6 and 7, but the magnitudes are smaller. These results suggest that the top‐income biases are at least as worrying in 2014 as in 2012. In 2010, after‐tax cash‐like transfers have an equalizing effect of 3 pc.pt. of the Gini, and 2.3 pc.pt. of the top‐10% income share, but these figures are not comparable to the effects of before‐tax transfers assessed in versions 2012–2014.
