# Supplementary material for: Redistributive effects of fiscal policies in Mexico: Corrections for top income measurement problems
Source: Lat Am Policy. 2021 Jun 10;12(1):148–80. doi: 10.1111/lamp.12206 (PMC8362083; doi:10.1111/lamp.12206)
Supplement: Supplementary file 1 — Supplementary Material [file LAMP-12-148-s001.docx]

**Appendix 1. Additional Summary Statistics and Estimation Results for Year 2014**

ENIGH 2014 covers 73,508 members of 19,479 households (domestic servants and guests excluded). The 19,479 fully interviewed households represent 88.2 percent of the 21,786 households (21,427 distinct dwellings) selected for participation under the sampling design. Another 838 households could not be fully interviewed even though they were contacted (type A nonresponse), 1,312 households could not be contacted because they were listed in unoccupied dwellings (type B), and 157 households were listed in what turned out to be invalid dwellings (type C).^[[1]](#footnote-1)^ INEGI does not conduct proxy interviews in place of units failing to respond to ENIGH, and does not perform imputation or top-coding of incomes.

The dataset available from the CEQ Institute includes information on household demographics and residence; level of current school attendance; employment and contract status; all core income concepts; various income sources, transfers and contributions; national poverty lines; indicators for participation in various transfer programs; sampling stratum and primary sampling unit (PSU) identifiers; selected household durable assets and utilities, financial asset indicators; and sampling weights.

Sampling weights correct for sampling bias and unit non-response bias at the level of PSUs, and inflate to the national population of 119.9 million. Item-nonresponse in the ENIGH was mitigated by telephone re-interviewing of households during the editing process. Incomes were checked for potential data entry errors.

ENIGH 2014 survey sample was obtained from a stratified multi-phase sample with 2,626 PSUs, that is, blocks of dwellings selected from state geostatistical areas stratified according to four geographic and socio-economic criteria (200 strata). Sampling frame was based on demographic and geographic information from the 2010 National Census (Censo de Población y Vivienda). Households were interviewed between August and November of the survey year.

Table A1-1. Population and sample sizes, non-response rates, and mean incomes by state

| State code | State | Represented population | Strata | PSUs | Fully interviewed households (individuals) | Type A non-responding HHs (%) | Mean market income  per cap. | Mean gross income  per cap. | Mean disposable income  per cap. | Mean final income  per cap. |
| --- | --- | --- | --- | --- | --- | --- | --- | --- | --- | --- |
| 01 | Aguascalientes | 1,273,449 | 5 | 90 | 553 (2,169) | 30 (5.1%) | 46,163 | 49,773 | 45,660 | 48,793 |
| 02 | Baja California | 3,445,408 | 5 | 96 | 547 (1,880) | 4 (0.7%) | 57,134 | 59,291 | 53,989 | 56,879 |
| 03 | Baja California Sur | 747,857 | 7 | 63 | 543 (1,916) | 5 (0.9%) | 54,577 | 57,688 | 52,122 | 56,378 |
| 04 | Campeche | 897,279 | 7 | 75 | 539 (2,100) | 34 (5.9%) | 46,698 | 49,368 | 45,192 | 48,956 |
| 05 | Coahuila de Zarag. | 2,940,002 | 5 | 96 | 515 (1,938) | 47 (8.4%) | 58,962 | 61,824 | 56,936 | 59,651 |
| 06 | Colima | 712,419 | 5 | 81 | 558 (1,987) | 14 (2.4%) | 50,086 | 53,440 | 49,476 | 52,661 |
| 07 | Chiapas | 5,182,656 | 6 | 48 | 565 (2,480) | 12 (2.1%) | 18,293 | 20,817 | 19,641 | 23,550 |
| 08 | Chihuahua | 3,681,549 | 7 | 93 | 548 (1,955) | 18 (3.2%) | 44,455 | 46,791 | 42,959 | 46,061 |
| 09 | Distrito Federal | 8,799,243 | 4 | 154 | 685 (2,423) | 53 (7.2%) | 81,471 | 86,106 | 77,703 | 79,085 |
| 10 | Durango | 1,752,061 | 6 | 78 | 549 (2,085) | 24 (4.2%) | 34,153 | 37,135 | 34,518 | 38,551 |
| 11 | Guanajuato | 5,783,856 | 6 | 71 | 548 (2,192) | 30 (5.2%) | 35,197 | 37,270 | 34,118 | 37,603 |
| 12 | Guerrero | 3,553,259 | 6 | 60 | 496 (2,042) | 49 (9.0%) | 25,258 | 28,055 | 26,554 | 30,612 |
| 13 | Hidalgo | 2,850,418 | 6 | 48 | 544 (2,004) | 17 (3.0%) | 32,455 | 35,382 | 33,268 | 37,190 |
| 14 | Jalisco | 7,855,551 | 6 | 92 | 601 (2,342) | 60 (9.1%) | 49,736 | 51,559 | 47,083 | 49,407 |
| 15 | México | 16,680,605 | 7 | 130 | 713 (2,878) | 38 (5.1%) | 42,718 | 44,711 | 39,859 | 43,012 |
| 16 | Mich. de Ocampo | 4,573,656 | 7 | 51 | 602 (2,361) | 4 (0.7%) | 29,104 | 31,866 | 29,983 | 33,632 |
| 17 | Morelos | 1,901,086 | 7 | 75 | 528 (1,873) | 28 (5.0%) | 39,324 | 43,812 | 41,834 | 45,010 |
| 18 | Nayarit | 1,207,885 | 7 | 63 | 550 (1,978) | 14 (2.5%) | 41,608 | 44,730 | 41,580 | 45,409 |
| 19 | Nuevo León | 5,024,096 | 6 | 116 | 616 (2,239) | 45 (6.8%) | 66,530 | 69,825 | 62,289 | 64,970 |
| 20 | Oaxaca | 3,988,647 | 6 | 45 | 569 (2,306) | 13 (2.2%) | 20,430 | 23,733 | 22,662 | 26,656 |
| 21 | Puebla | 6,137,672 | 8 | 77 | 641 (2,592) | 34 (5.0%) | 30,072 | 31,955 | 30,129 | 33,717 |
| 22 | Querétaro | 1,980,758 | 6 | 75 | 573 (2,208) | 5 (0.9%) | 50,522 | 52,967 | 48,065 | 50,479 |
| 23 | Quintana Roo | 1,540,017 | 6 | 92 | 517 (1,922) | 25 (4.6%) | 51,554 | 52,882 | 47,841 | 51,202 |
| 24 | San Luis Potosí | 2,728,705 | 6 | 69 | 517 (2,025) | 53 (9.3%) | 34,697 | 37,769 | 35,244 | 39,613 |
| 25 | Sinaloa | 2,960,736 | 6 | 78 | 585 (2,114) | 1 (0.2%) | 48,545 | 52,576 | 48,319 | 52,940 |
| 26 | Sonora | 2,902,867 | 8 | 84 | 547 (1,930) | 14 (2.5%) | 54,927 | 58,384 | 53,468 | 57,705 |
| 27 | Tabasco | 2,359,988 | 8 | 152 | 1,885 (7,069) | 47 (2.4%) | 37,257 | 39,919 | 37,282 | 41,197 |
| 28 | Tamaulipas | 3,511,188 | 5 | 93 | 536 (1,885) | 31 (5.5%) | 49,401 | 53,077 | 48,380 | 51,934 |
| 29 | Tlaxcala | 1,266,058 | 5 | 78 | 533 (2,228) | 26 (4.7%) | 27,577 | 29,600 | 28,030 | 32,308 |
| 30 | Veracruz de Ignacio | 8,004,171 | 7 | 74 | 679 (2,417) | 8 (1.2%) | 29,721 | 32,790 | 30,841 | 34,110 |
| 31 | Yucatán | 2,096,010 | 7 | 78 | 539 (2,067) | 36 (6.3%) | 40,605 | 44,357 | 41,072 | 44,234 |
| 32 | Zacatecas | 1,567,160 | 7 | 51 | 558 (1,903) | 19 (3.3%) | 32,298 | 35,994 | 34,220 | 37,823 |
|  | Nationwide | 119,906,312 | 200 | 2,626 | 19,479 (73,508) | 838 (4.1%) | 43,178 | 45,985 | 42,117 | 45,374 |

Note: Calculation of nonresponse rates omits type B and C nonresponses. Mean incomes account for sampling weights but are computed only among responding households, and may not be representative of underlying population.

Source: Instituto Nacional de Estadística y Geografía (2016), Encuesta Nacional de Ingresos y Gastos de los Hogares ENIGH 2014: Operative de Campo, México: INEGI; own analysis of ENIGH 2014, CEQ database.

Table A1-2. Estimation results for selected multivariate models of response probability

|  |  |  |  |  |  |  |  |  | Gini (s.e.): Market inc. pc. | | |
| --- | --- | --- | --- | --- | --- | --- | --- | --- | --- | --- | --- |
| Specification of *g(x)* | $\hat{\theta_{0}}$ (s.e.) | $\hat{\theta_{1}}$ (s.e.) | $\hat{\theta_{2}}$ (s.e.) | $\hat{\theta_{3}}$ (s.e.) | $\hat{\theta_{4}}$ (s.e.) | Sum of squared wghted. errors | Factor of propor-tionality (σ^2^) | AIC  SIC | Weighted data | HH-size wted, no sampling wghts | Equal HH weights=  unwghtd |
| Uncorrected |  |  |  |  |  |  |  |  | 52.75  (0.82) | 50.63 (0.45) | 52.84  (0.49) |
| θ_0_+θ_1_10^-6^mkt. inc.  +θ_2_10^-15^mkt. inc.^2^ | 3.274 (0.003) | -1.196 (0.006) | 48.914 (0.370) |  |  | 37,501 | 0.449 | 232.12  229.42 | 61.68 (5.91) | 57.94 (4.80) | 59.07 (3.62) |
| θ_0_+θ_1_10^-6^mkt. inc.pc  +θ_2_10^-12^mkt. inc.pc^2^ | 3.220 (0.003 | -2.888 (0.027) | 0.341 (0.006) |  |  | 39,284 | 0.463 | 233.61  230.91 | 60.16 (5.33) | 57.13 (4.16) | 60.50 (3.87) |
| θ_0_+θ_1_log(mkt. inc.) +θ_2_urban/10+θ_3_age+θ_4_age^2^ | 133.821 (38∙10^3^) | -0.147 (0.007) | -150.00 (38∙10^4^) | -370.00 (13.78) | 290.24 (11.09) | 27,883 | 0.300 | 226.64  224.69 | 52.58 (0.81) | 50.52 (0.44) | 52.69 (0.49) |
| θ_0_+θ_1_log(mkt. inc.) +θ_2_sch.attend+θ_3_age+θ_4_age^2^ | 11.896 (0.141) | -0.651 (0.008) | -0.295 (0.013) | -1.842 (0.640) | -2.262 (0.686) | 34,261 | 0.431 | 233.23  231.28 | 54.05 (1.07) | 51.56 (0.65) | 53.76 (0.63) |
| θ_0_+θ_1_log(mkt. inc.) +θ_2_urban+θ_3_HH size | 24.037 (14∙10^3^) | -0.535 (0.010) | -13.963 (14∙10^3^) | -28.471 (0.428) |  | 31,849 | 0.371 | 228.90  226.44 | 53.17 (0.99) | 50.83 (0.58) | 53.07 (0.56) |
| θ_0_+θ_1_log(mkt. inc.) +θ_2_urb+θ_3_hsize+θ_4_10hsize^2^ | 207.895(19∙10^3^) | -1.088 (0.006) | -3.229 (0.307) | -7∙10^3^ (.7∙10^6^) | 6.3∙10^3^ (.7∙10^6^) | 24,833 | 0.321 | 222.93  220.98 | 51.88 (0.80) | 49.88 (0.45) | 52.31 (0.48) |
| θ_0_+θ_1_log(mkt. inc.) +θ_2_male+θ_3_hsize | 16.995 (0.102) | -1.102 (0.007) | 0.050 (0.042) | -23.743 (0.456) |  | 33,022 | 0.426 | 230.05  227.60 | 57.09 (2.60) | 53.64 (1.94) | 55.46 (1.52) |
| θ_0_+θ_1_log(mkt. inc.) +θ_2_male+θ_3_urban | 24.206 (12∙10^4^) | -0.427 (0.009) | 0.316 (0.041) | -16.641 (12∙10^4^) |  | 34,241 | 0.400 | 231.21  228.76 | 53.36 (0.94) | 51.03 (0.53) | 53.22 (0.54) |
| θ_0_+θ_1_log(mkt. inc.) +θ_2_postsecondary.attend | 10.741 (0.105) | -0.641 (0.009) | -2.490 (0.033) |  |  | 34,702 | 0.445 | 229.64  226.94 | 54.13 (1.03) | 51.67 (0.62) | 53.87 (0.62) |
| θ_0_+θ_1_log(mkt. inc.) +θ_2_postsec.attend+θ_3_formal | 3.264 (0.055) | -0.149 (0.005) | -1.218 (0.038) | -0.795 (0.010) |  | 84,202 | 1.347 | 260.01  257.55 | 54.20 (1.06) | 51.64 (0.61) | 53.92 (0.62) |
| θ_0_+θ_1_log(mkt. inc.)  +θ_2_/100log(mkt. inc.)^2^ | -5.741 (0.253) | 2.223 (0.037) | -12.428 (0.135) |  |  | 33,405 | 0.408 | 228.42 225.72 | 61.90 (6.06) | 57.62 (4.95) | 58.78 (3.69) |
| θ_0_+θ_1_log(mkt. inc.) +θ_2_sch.attend+θ_3_age+θ_4_age^2^+θ_5_log(inc)^2^+θ_6_log(inc)^3^ |  |  |  |  |  |  |  |  |  |  |  |

Note: Standard errors on Gini coefficients are jackknife estimates. Variables are normalized: age=(years-12)/100; HH size=(#-1)/100. Measures of fit are not entirely comparable across models with different controls, because of different sample sizes.

Source: Own analysis of ENIGH 2014, CEQ database.

Table A1-3. Distribution of top incomes, various income concepts: Pareto (type I) estimates

|  | (1) | (2) | (3) | (4) | (5) | (6) | (7) | (8) |
| --- | --- | --- | --- | --- | --- | --- | --- | --- |
|  | Market income per cap. | Market inc + pensions  per cap. | Gross income per cap. | Taxable income per cap. | Net market income per cap. | Disposable income per cap. | Consumable income per cap. | Final income per cap. |
| Pareto (type I) coefficient $\alpha$ | | |  |  |  |  |  |  |
| Top 25% | 1.62 (.03) | 1.59 (.03) | 1.60 (.03) | 1.59 (.03) | 1.65 (.03) | 1.66 (.03) | 1.67 (.03) | 1.78 (.03) |
| Top 10% | 1.76 (.05) | 1.77 (.05) | 1.79 (.06) | 1.77 (.06) | 1.82 (.05) | 1.83 (.06) | 1.85 (.06) | 1.94 (.06) |
| Top 5% | 1.84 (.09) | 1.89 (.09) | 1.91 (.09) | 1.84 (.09) | 1.93 (.09) | 1.94 (.09) | 1.94 (.09) | 2.00 (.09) |
| Top 1% | 1.99 (.22) | 2.03 (.22) | 2.06 (.22) | 1.97 (.20) | 2.04 (.20) | 2.04 (.20) | 2.06 (.21) | 2.09 (.21) |
| Top 0.1% | 3.61 (1.22) | 2.73 (1.05) | 2.73 (1.05) | 3.42 (2.23) | 3.33 (2.04) | 3.32 (2.04) | 3.37 (2.02) | 3.38 (2.07) |
|  |  |  |  |  |  |  |  |  |
| Inverted Pareto coefficient $\beta=\alpha/\left[ \alpha-1 \right]$ | | | |  |  |  |  |  |
| Top 25% | 2.60 | 2.69 | 2.66 | 2.70 | 2.54 | 2.51 | 2.49 | 2.29 |
| Top 10% | 2.31 | 2.30 | 2.27 | 2.29 | 2.22 | 2.21 | 2.18 | 2.06 |
| Top 5% | 2.19 | 2.13 | 2.10 | 2.19 | 2.08 | 2.07 | 2.06 | 2.00 |
| Top 1% | 2.01 | 1.97 | 1.95 | 2.03 | 1.96 | 1.96 | 1.94 | 1.92 |
| Top 0.1% | 1.38 | 1.58 | 1.58 | 1.41 | 1.43 | 1.43 | 1.42 | 1.42 |
|  |  |  |  |  |  |  |  |  |
| Top income share (%) | |  |  |  |  |  |  |  |
| Top 25% | 65.57 | 66.24 | 65.03 | 67.96 | 64.59 | 63.31 | 62.9 | 58.64 |
| Top 10% | 43.72 | 43.9 | 42.85 | 44.84 | 42.4 | 41.45 | 40.93 | 37.36 |
| Top 5% | 31.37 | 31.13 | 30.4 | 32.4 | 30 | 29.27 | 29.03 | 26.29 |
| Top 1% | 14.09 | 13.6 | 13.32 | 14.77 | 12.97 | 12.67 | 12.46 | 11.22 |
| Top 0.1% | 3.33 | 3.25 | 3.17 | 3.16 | 2.64 | 2.57 | 2.52 | 2.26 |
|  |  |  |  |  |  |  |  |  |
| Gini among top incomes (×100) | | |  |  |  |  |  |  |
| Top 25% | 44.48 | 45.78 | 45.37 | 45.92 | 43.53 | 43.14 | 42.70 | 39.17 |
| Top 10% | 39.76 | 39.37 | 38.70 | 39.28 | 37.94 | 37.65 | 37.02 | 34.73 |
| Top 5% | 37.22 | 35.85 | 35.35 | 37.40 | 34.95 | 34.74 | 34.86 | 33.44 |
| Top 1% | 33.65 | 33.44 | 32.95 | 33.97 | 32.72 | 32.80 | 32.20 | 31.56 |
| Top 0.1% | 19.21 | 20.80 | 20.84 | 16.53 | 16.60 | 16.63 | 16.73 | 16.47 |
|  |  |  |  |  |  |  |  |  |
| Half coefficient of variation squared^i^ | | |  |  |  |  |  |  |
| Top 10% | -- | -- | -- | -- | -- | -- | -- | -- |
| Top 5% | -- | -- | -- | -- | -- | -- | -- | -- |
| Top 1% | -- | 3.66 | 2.14 | -- | 2.95 | 3.28 | 1.81 | 1.24 |
| Top 0.1% | 0.024 | 0.092 | 0.093 | 0.030 | 0.034 | 0.034 | 0.032 | 0.032 |
|  |  |  |  |  |  |  |  |  |
| Minimum income | |  |  |  |  |  |  |  |
| Top 25% | 46,477 | 48,042 | 48,594 | 32,983 | 44,590 | 45,144 | 43,769 | 48,405 |
| Top 10% | 83,714 | 88,416 | 89,443 | 60,492 | 80,362 | 81,033 | 78,427 | 83,243 |
| Top 5% | 125,755 | 133,943 | 135,000 | 90,492 | 120,148 | 120,867 | 115,837 | 120,053 |
| Top 1% | 304,486 | 311,864 | 311,864 | 221,505 | 273,162 | 273,162 | 262,714 | 265,543 |
| Top 0.1% | 1,052,184 | 1,051,861 | 1,051,861 | 776,087 | 918,303 | 918,303 | 885,516 | 887,620 |
|  |  |  |  |  |  |  |  |  |
| Mean income | |  |  |  |  |  |  |  |
| Top 25% | 120,970 | 129,222 | 129,213 | 89,079 | 113,341 | 113,362 | 108,838 | 110,782 |
| Top 10% | 193,608 | 203,344 | 202,739 | 138,770 | 178,530 | 178,900 | 170,679 | 171,801 |
| Top 5% | 275,295 | 284,737 | 283,212 | 198,524 | 249,688 | 249,705 | 238,568 | 240,687 |
| Top 1% | 611,169 | 613,757 | 607,382 | 450,317 | 535,633 | 536,631 | 509,466 | 508,646 |
| Top 0.1% | 1,455,471 | 1,659,742 | 1,661,022 | 1,096,199 | 1,313,212 | 1,314,199 | 1,259,601 | 1,261,269 |
|  |  |  |  |  |  |  |  |  |

^i^ Unable to calculate for other top income groups.

Source: Own analysis of ENIGH 2014, CEQ database.

Table A1-4. Generalized Pareto (type II) results, various income concepts (individual sampling-weighted sample)

|  | Market income per cap. | Market income + pensions  per cap. | Gross income per cap. | | Taxable income per cap. | Net market income per cap. | Disposable income per cap. | Consumable income per cap. | Final income per cap. |
| --- | --- | --- | --- | --- | --- | --- | --- | --- | --- |
| Pareto (type II) shape coefficient ξ | | | | |  |  |  |  |  |
| Top 25% | 0.50 (.02) | 0.48 (.02) | 0.48 (.02) | | 0.48 (.02) | 0.48 (.02) | 0.48 (.02) | 0.48 (.02) | 0.45 (.02) |
| Top 10% | 0.48 (.03) | 0.45 (.03) | 0.46 (.03) | | 0.51 (.03) | 0.45 (.03) | 0.45 (.03) | 0.45 (.03) | 0.46 (.03) |
| Top 5% | 0.48 (.04) | 0.45 (.04) | 0.46 (.04) | | 0.51 (.04) | 0.44 (.04) | 0.44 (.04) | 0.44 (.04) | 0.44 (.04) |
| Top 1% | 0.52 (.08) | 0.53 (.09) | 0.55 (.09) | | 0.46 (.08) | 0.41 (.08) | 0.41 (.08) | 0.43 (.08) | 0.44 (.08) |
| Top 0.1% | 0.78 (.43) | 0.38 (.13) | 0.38 (.13) | | 1.59 (.34) | 1.11 (.34) | 1.12 (.34) | 1.04 (.26) | 1.04 (.26) |
|  |  |  |  | |  |  |  |  |  |
| Pareto (type II) scale coefficient, log(σ) | | | | |  |  |  |  |  |
| Top 25% | 10.37 (.02) | 10.46 (.02) | 10.46 (.02) | | 10.09 (.02) | 10.33 (.02) | 10.33 (.02) | 10.29 (.02) | 10.32 (.02) |
| Top 10% | 10.85 (.03) | 10.93 (.03) | 10.92 (.03) | | 10.49 (.03) | 10.80 (.03) | 10.80 (.03) | 10.74 (.03) | 10.73 (.03) |
| Top 5% | 11.19 (.04) | 11.25 (.04) | 11.23 (.04) | | 10.84 (.04) | 11.12 (.04) | 11.12 (.04) | 11.07 (.04) | 11.07 (.04) |
| Top 1% | 11.92 (.13) | 11.91 (.14) | 11.87 (.14) | | 11.68 (.12) | 11.88 (.12) | 11.89 (.11) | 11.81 (.12) | 11.78 (.12) |
| Top 0.1% | 12.01 (.54) | 12.85 (.19) | 12.85 (.20) | | 10.39 (.21) | 11.49 (.39) | 11.49 (.39) | 11.53 (.25) | 11.52 (.25) |
|  |  |  |  | |  |  |  |  |  |
| 1/ξ | | |  | |  |  |  |  |  |
| Top 25% | 1.99 | 2.07 | 2.07 | | 2.10 | 2.08 | 2.07 | 2.10 | 2.20 |
| Top 10% | 2.08 | 2.21 | 2.18 | | 1.97 | 2.23 | 2.23 | 2.20 | 2.20 |
| Top 5% | 2.07 | 2.22 | 2.17 | | 1.96 | 2.28 | 2.28 | 2.29 | 2.29 |
| Top 1% | 1.93 | 1.90 | 1.83 | | 2.17 | 2.42 | 2.44 | 2.31 | 2.25 |
| Top 0.1% | 1.29 | 2.61 | 2.62 | | 0.63 | 0.90 | 0.90 | 0.97 | 0.96 |
|  |  |  |  | |  |  |  |  |  |
| Inverted Pareto coefficient | | |  | |  |  |  |  |  |
| Top 25% | 2.01 | 1.94 | 1.93 | | 1.91 | 1.93 | 1.93 | 1.91 | 1.83 |
| Top 10% | 1.92 | 1.83 | 1.85 | | 2.03 | 1.81 | 1.81 | 1.83 | 1.84 |
| Top 5% | 1.94 | 1.82 | 1.86 | | 2.04 | 1.78 | 1.78 | 1.77 | 1.78 |
| Top 1% | 2.08 | 2.11 | 2.21 | | 1.85 | 1.71 | 1.69 | 1.76 | 1.80 |
| Top 0.1% | 4.50 | 1.62 | 1.62 | | -1.70 | -8.72 | -8.56 | -28.18 | -25.81 |
|  |  |  |  | |  |  |  |  |  |
| Gini among top incomes | |  |  | |  |  |  |  |  |
| Top 25% | 38.76 | 38.45 | 38.25 | | 38.19 | 37.50 | 37.34 | 36.91 | 34.56 |
| Top 10% | 35.75 | 34.59 | 34.56 | | 36.67 | 33.79 | 33.66 | 33.54 | 32.43 |
| Top 5% | 34.79 | 32.94 | 33.01 | | 35.88 | 32.03 | 31.89 | 31.74 | 31.14 |
| Top 1% | 34.17 | 33.93 | 34.57 | | 32.27 | 29.87 | 29.81 | 30.19 | 30.26 |
| Top 0.1% | 33.82 | 22.78 | 22.78 | | -2.86 | 41.97 | 41.45 | 73.52 | 71.53 |
|  |  |  |  | |  |  |  |  |  |
| Log pseudo-likelihood (LL/10^6^) | | | |  |  |  |  |  |  |
| Top 25% | -356.00 | -358.00 | -358.00 | | -347.00 | -354.00 | -354.00 | -353.00 | -353.00 |
| Top 10% | -148.00 | -148.00 | -148.00 | | -144.00 | -147.00 | -147.00 | -146.00 | -146.00 |
| Top 5% | -76.00 | -76.00 | -76.10 | | -74.00 | -75.20 | -75.30 | -75.20 | -74.90 |
| Top 1% | -16.10 | -16.20 | -16.40 | | -15.70 | -16.00 | -16.00 | -15.90 | -16.00 |
| Top 0.1% | -1.86 | -1.52 | -1.52 | | -1.36 | -1.35 | -1.35 | -1.33 | -1.33 |
|  |  |  |  | |  |  |  |  |  |

Source: Own analysis of ENIGH 2014, CEQ database.

Figure A1-1. Mean observed market income per capita among respondents, and nonresponse rate, by state


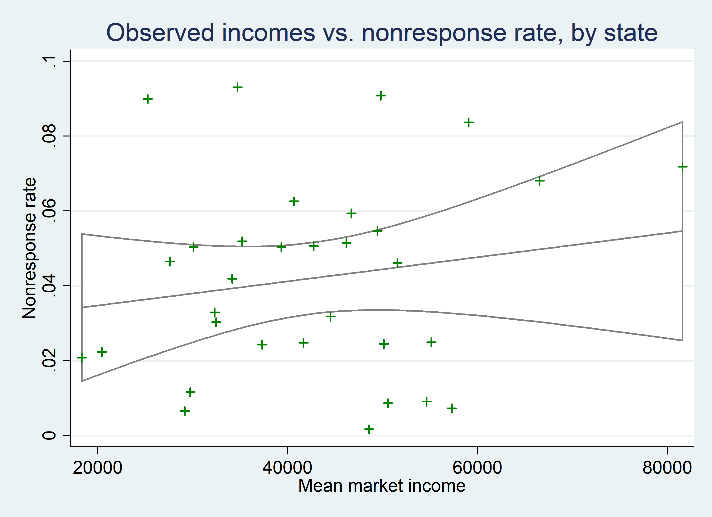


Notes: 95% confidence interval around linear fitted line is shown.

Source: Own analysis of ENIGH 2014, CEQ database.

Figure A1-2. Density function of income per capita, with reference lognormal density


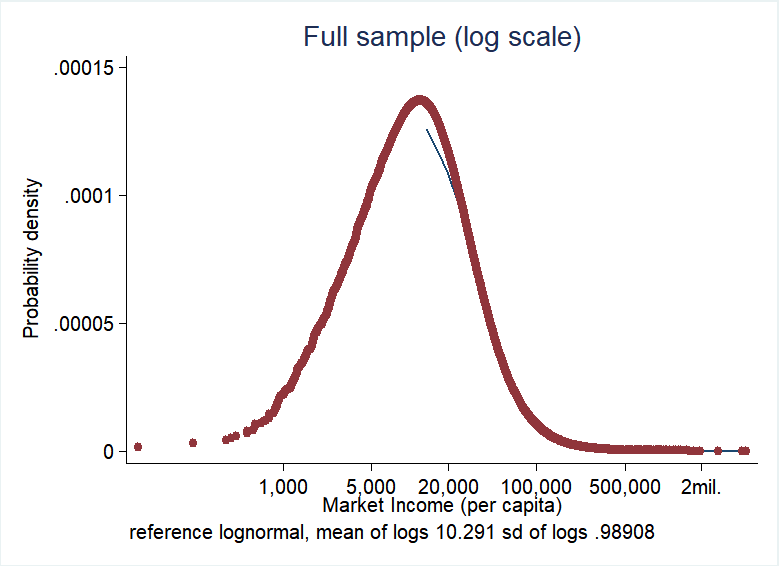

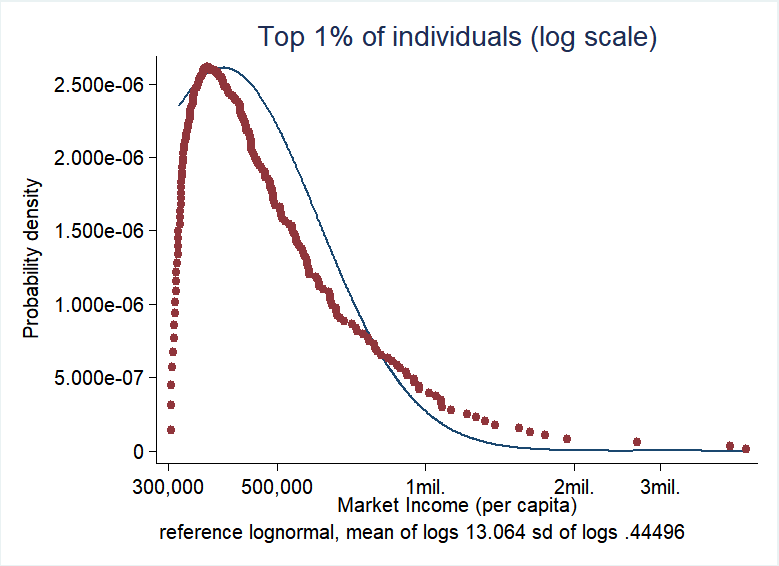


i. Market income, full sample i. Market income, top 1%


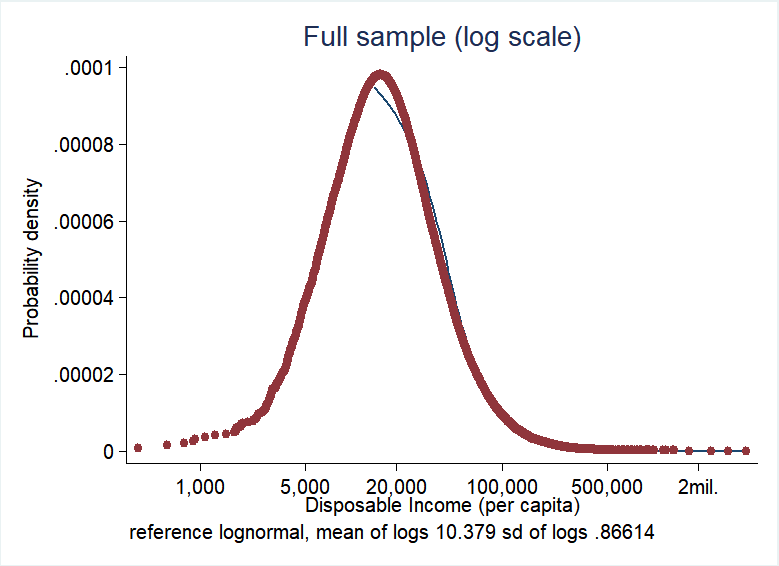

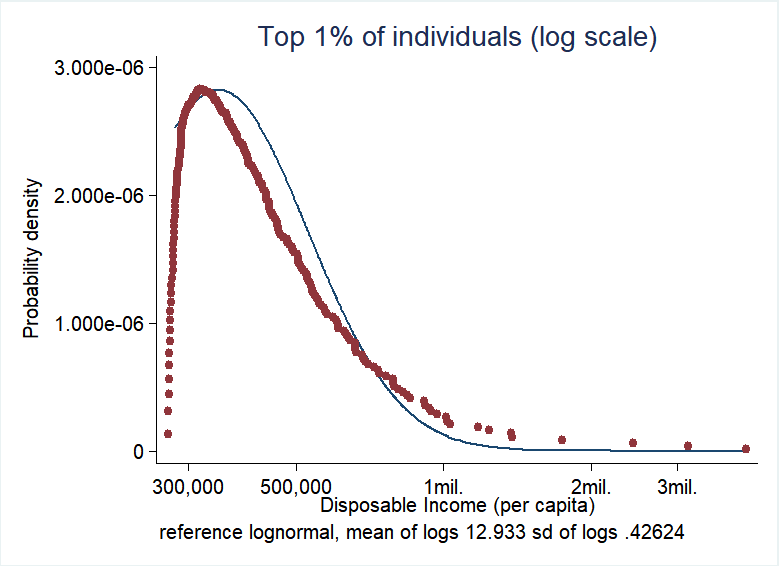


iii. Disposable income, full sample i. Disposable income, top 1%

Notes: Non-positive incomes omitted.

Source: Own analysis of ENIGH 2014, CEQ database.

Figure A1-3. Cumulative density function of disposable income per capita (log scale)


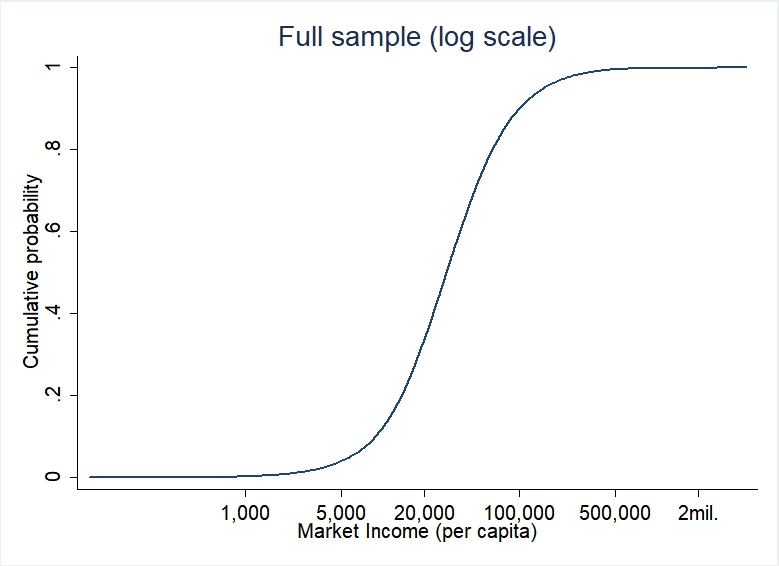

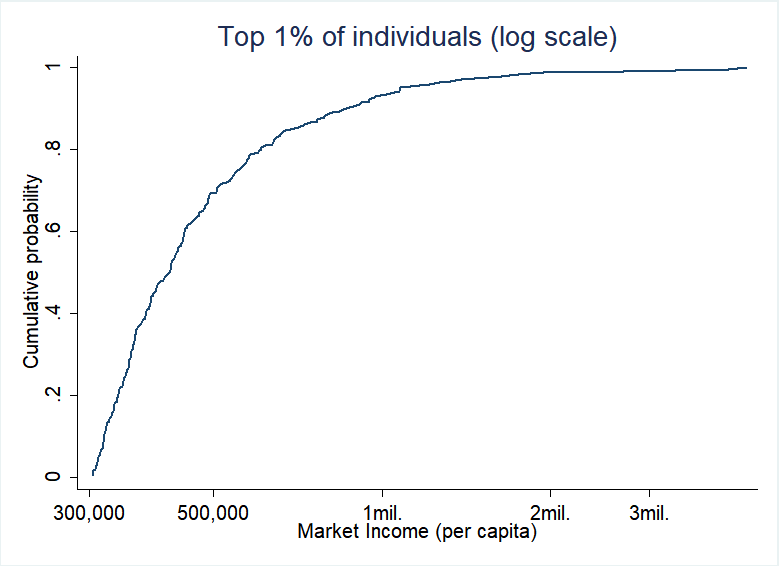


i. Market income, full sample i. Market income, top 1%


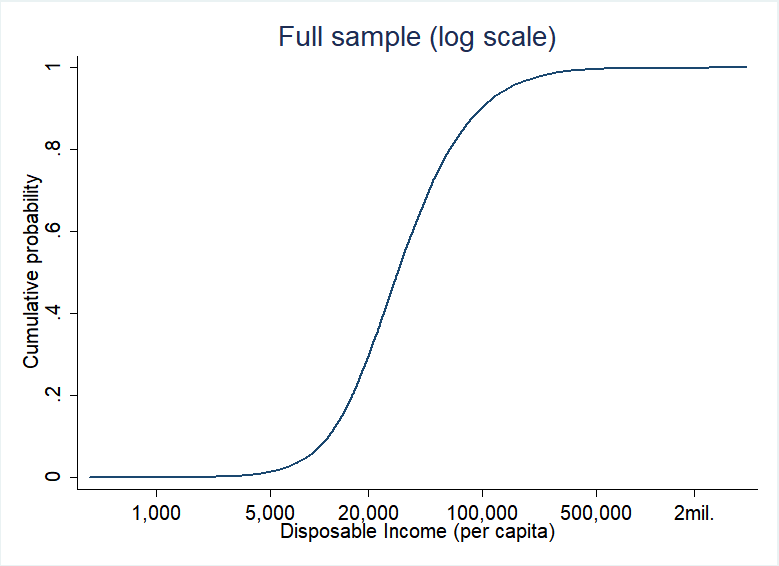

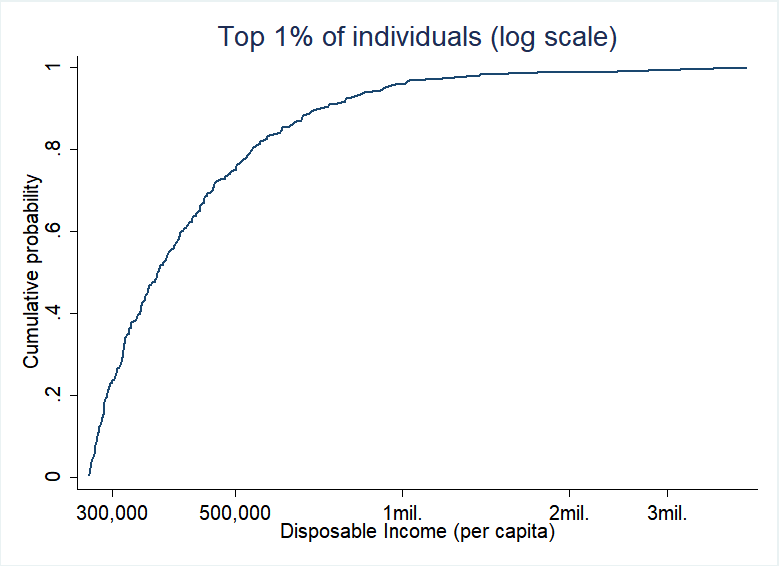


iii. Disposable income, full sample i. Disposable income, top 1%

Source: Own analysis of ENIGH 2014, CEQ database.

Figure A1-4. Lorenz curve: market and disposable income per capita, uncorrected versus unit-nonresponse corrected weights (model 1)


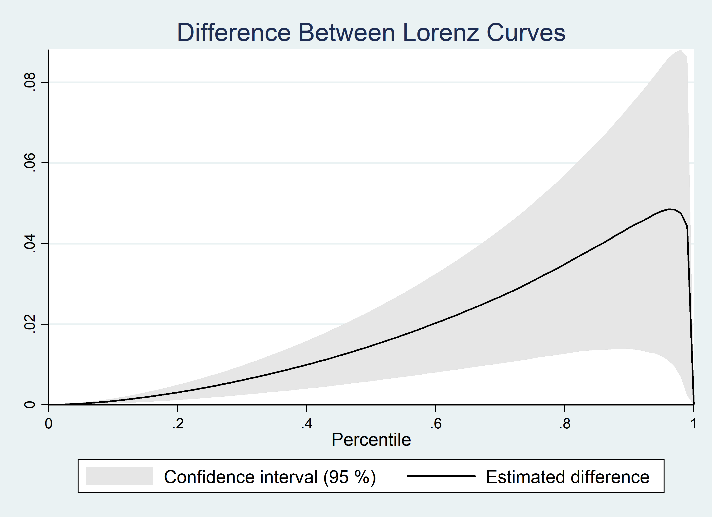

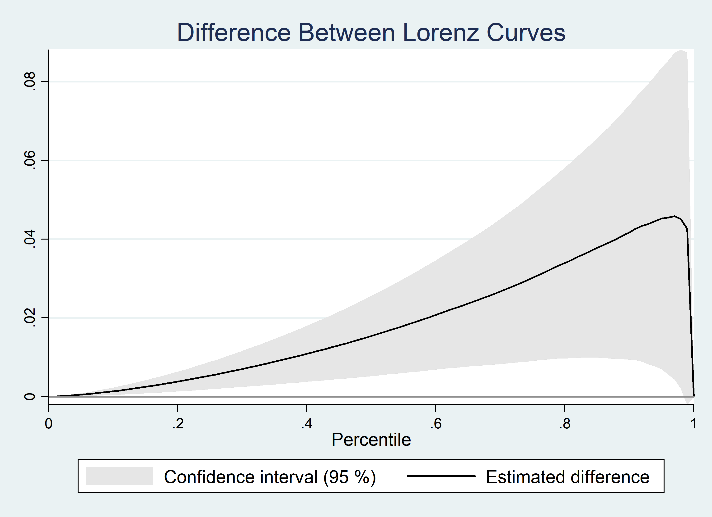


i. Market income per capita i. Disposable income per capita

Notes: Positive values indicate that the Lorenz curve uncorrected for unit-nonresponse dominates, and shows less inequality than the corrected Lorenz curve. Distributions account for sampling weights and household size.

Figure A1-5. Unit response probability by gross income per capita, logarithmic vs. quadratic model


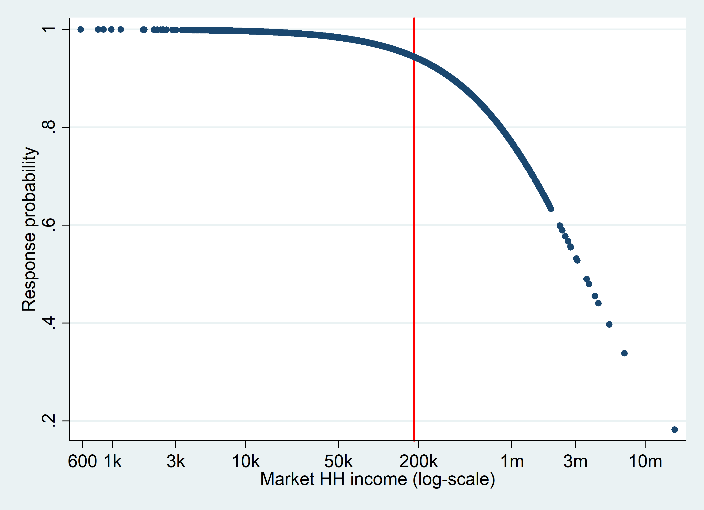

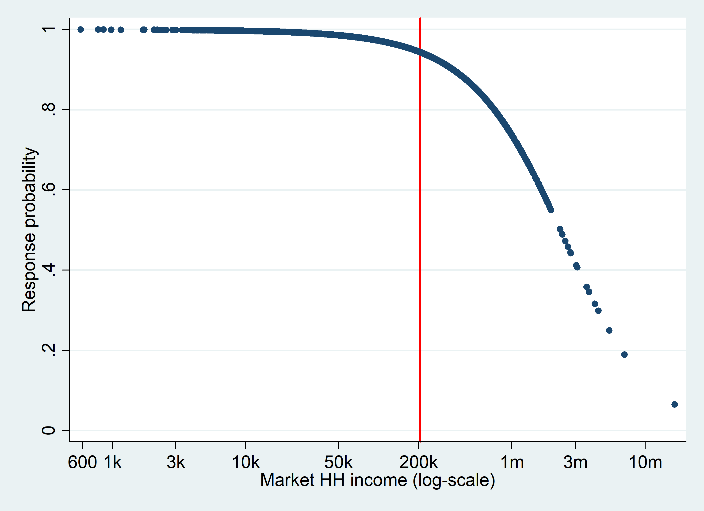


i. Logarithmic model of market income (model 1) ii. Quadratic logarithmic model of market income

θ_0_+θ_1_log(market inc.) (Model 7) θ_0_+θ_1_log(market inc.)^2^

Notes: Red line shows mean market household income in the corrected income distribution: i) 184,505 and ii) 203,382.

Source: Own analysis of ENIGH 2014, CEQ database.

Figure A1-6. Lorenz curve: market and disposable income per capita, top 10% of incomes replaced with Pareto I values


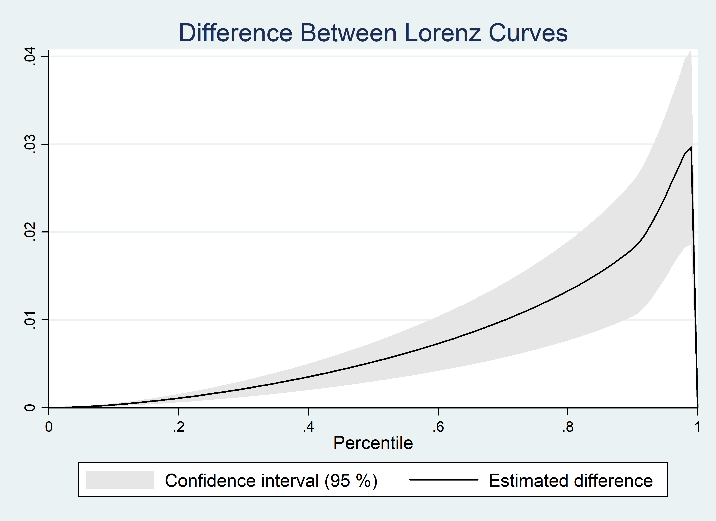

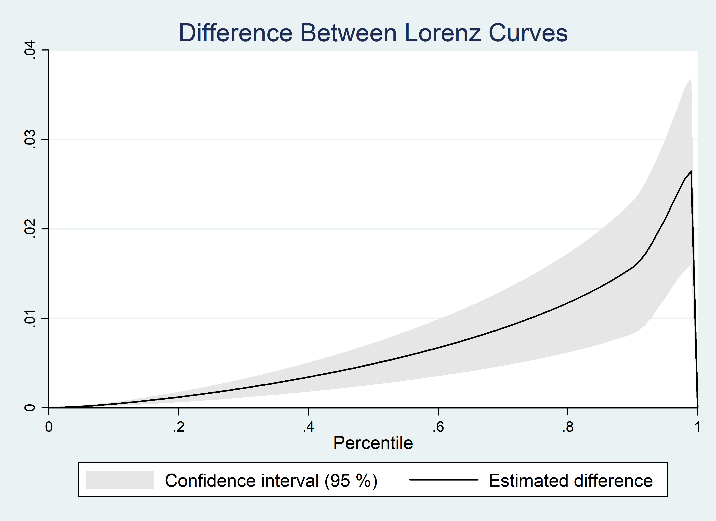


i. Market income per capita i. Disposable income per capita

Notes: Pareto replacing is performed on own income concept. Positive values indicate that the uncorrected Lorenz curve dominates, and shows less inequality than the corrected Lorenz curve. Distributions account for sampling weights and household size.

Figure A1-7. Lorenz curve: market and disposable income per capita, top 10% of net market incomes replaced with Pareto I values, other income concepts imputed using CEQ Method


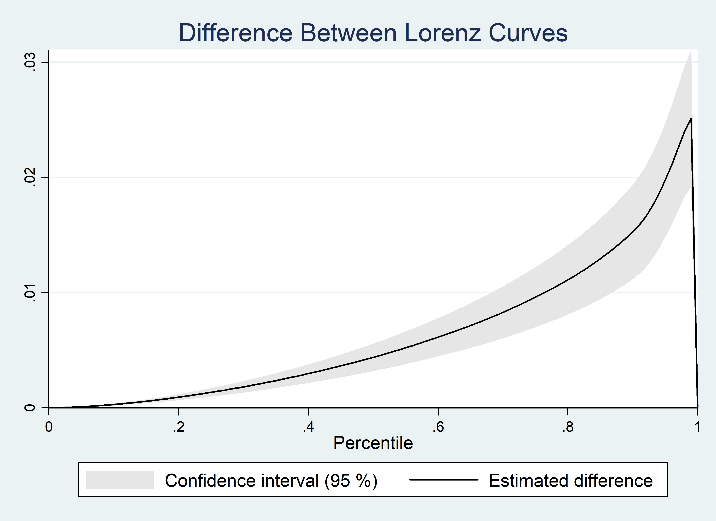

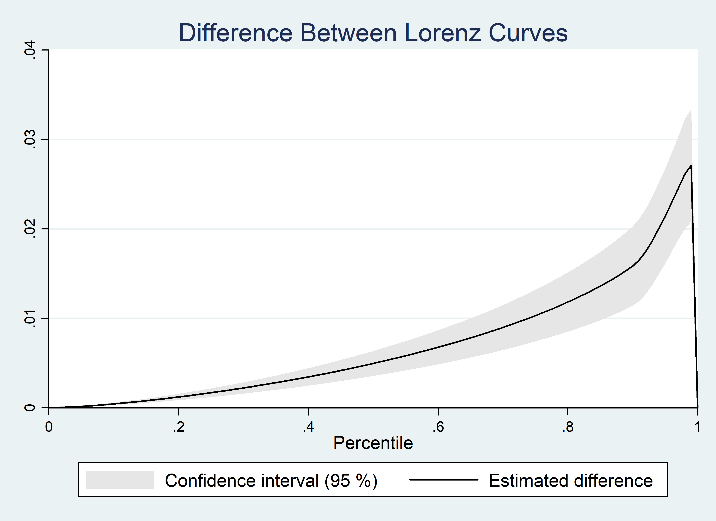


i. Market income per capita i. Disposable income per capita

Notes: Positive values indicate that the uncorrected Lorenz curve dominates, and shows less inequality than the corrected Lorenz curve. Distributions account for sampling weights and household size.

Figure A1-8. Comparison of Pareto I and Pareto II models, various income concepts and top income cutoffs


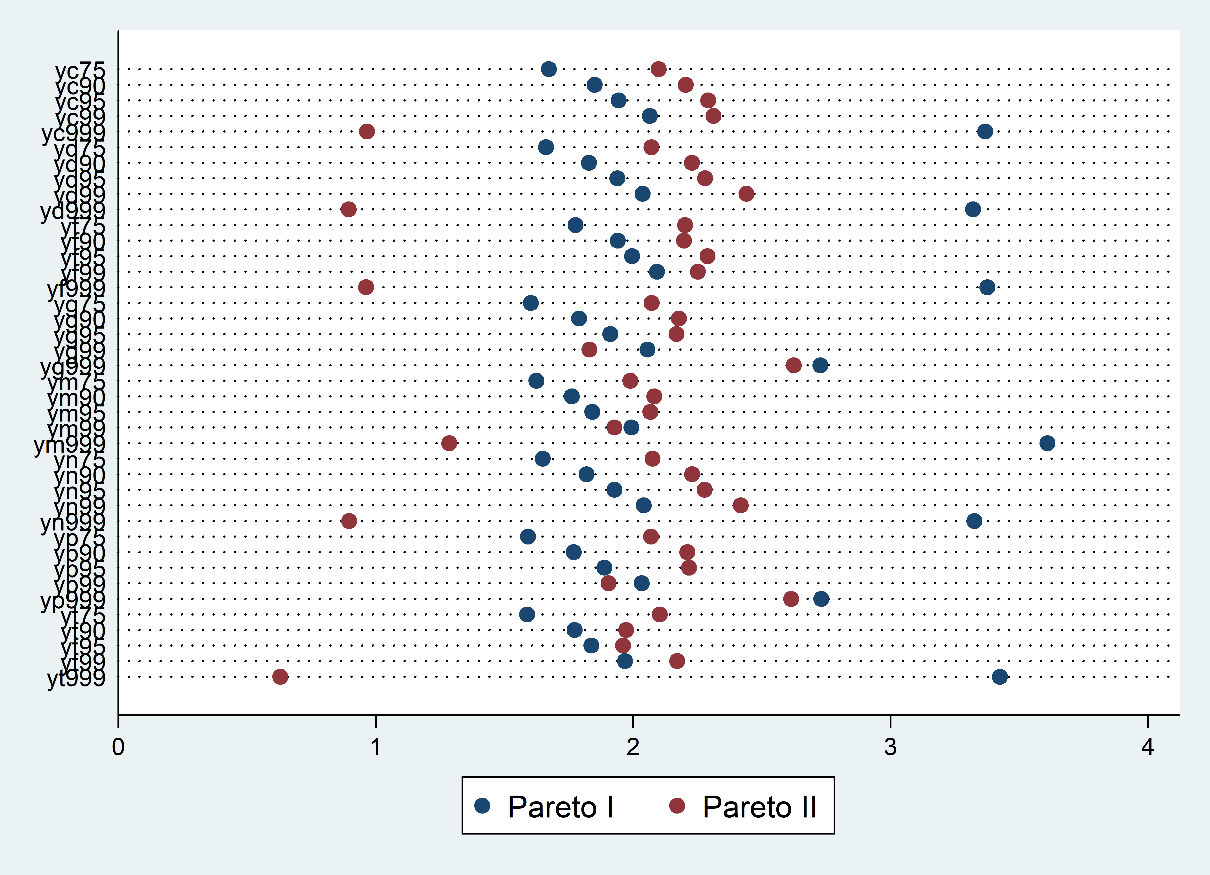


i. Pareto coefficient


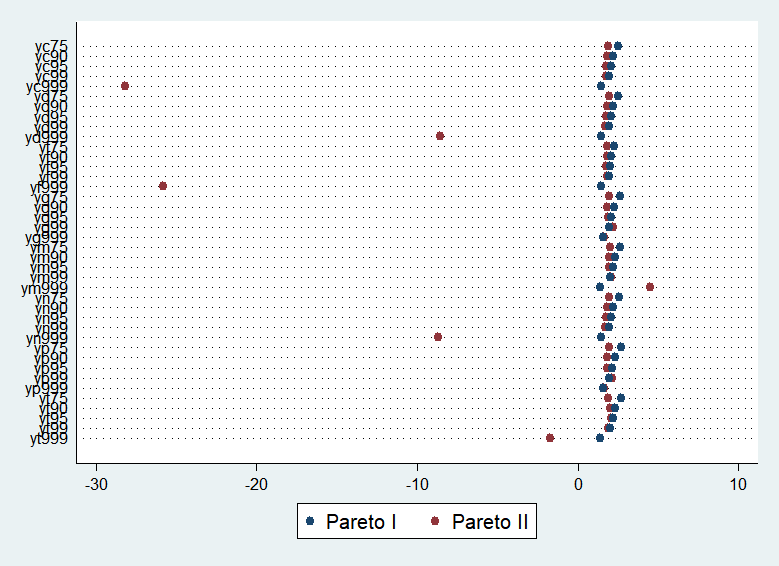


ii. Inverted Pareto coefficient


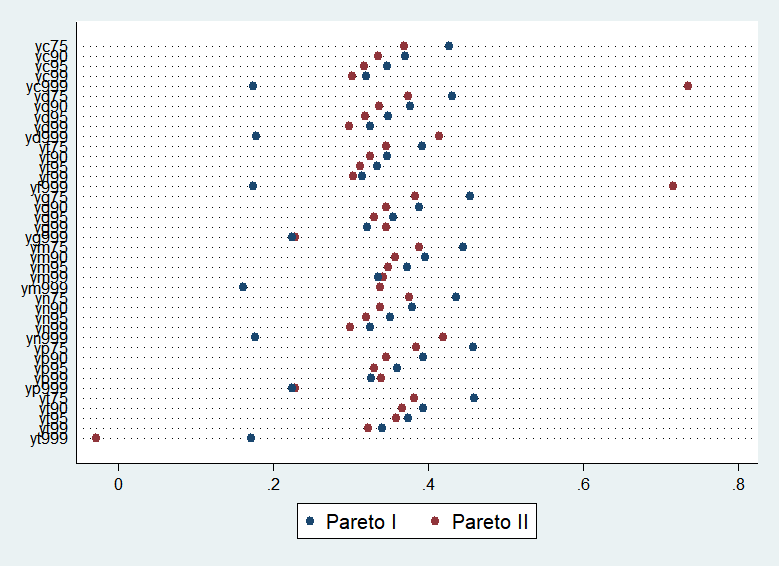


iii. Gini coefficient

Notes: ‘yp’ is market income plus pensions, ‘ym’ market income, ‘yt’ taxable income, ‘yg’ gross income, ‘yn’ net market income, ‘yd’ disposable income, ‘yc’ consumable income, ‘yf’ final income.

Source: Own analysis of ENIGH 2014, CEQ database.

**Appendix 2. Summary Statistics and Estimation Results for ENIGH 2012**

Survey sample in ENIGH 2012 was obtained from a stratified multi-phase sample with 1,111 primary sampling units (PSUs), that is, blocks of dwellings selected from state geostatistical areas stratified according to four geographic and socio-economic criteria (123 strata). Sampling frame was based on demographic and geographic information from the 2010 National Census (Censo de Población y Vivienda).^[[2]](#footnote-2)^ Item-nonresponse was mitigated by telephone re-interviewing households during the editing process. Incomes were checked for potential data entry errors. INEGI does not perform imputation or top-coding of incomes.

ENIGH 2012 covers 33,694 members of 9,002 households (plus 32 domestic servants and guests). The 9,002 fully interviewed households represent 88.2 percent of the 10,210 households (10,062 distinct dwellings) selected for participation under the sampling design. Another 252 households could not be fully interviewed even though they were contacted (type A nonresponse), 839 households could not be contacted because they were listed in unoccupied dwellings (type B), and 117 households were listed in what turned out to be invalid dwellings (type C).^[[3]](#footnote-3)^ INEGI does not use replacement units for those failing to respond to ENIGH. Sampling weights correct for sampling bias and unit non-response bias at the level of PSUs, and inflate to the national population of 117.3 million (source: own analysis of ENIGH 2012). Tables A2-1 through A2-3 provide basic descriptive statistics of the Mexican ENIGH 2012 sample.

Table A2-1a. Income summary statistics, various income concepts (sampling-weighted sample)

|  | Market income per cap. | Market inc +pensions  per cap. | Gross income per cap. | Taxable income per cap. | Net market income per cap. | Disposable income per cap. | Consumable income per cap. | Final income per cap. |
| --- | --- | --- | --- | --- | --- | --- | --- | --- |
| 99.9^th^ %ile | 610,435 | 642,581 | 642,581 | 515,538 | 536,234 | 536,234 | 524,765 | 526,396 |
| 99^th^ %ile | 274,420 | 294,574 | 295,241 | 203,607 | 264,017 | 264,017 | 260,020 | 261,362 |
| 95^th^ %ile | 128,837 | 137,814 | 139,344 | 92,348 | 123,945 | 124,248 | 120,696 | 125,845 |
| 90^th^ %ile | 84,011 | 88,878 | 90,000 | 59,957 | 82,148 | 82,279 | 80,844 | 84,746 |
| 75^th^ %ile | 45,967 | 48,508 | 49,088 | 32,772 | 44,992 | 45,367 | 44,792 | 49,133 |
| Mean | 41,290 | 43,428 | 44,327 | 28,850 | 40,126 | 41,025 | 40,410 | 44,712 |
| Median | 25,249 | 26,253 | 27,012 | 17,648 | 25,196 | 25,768 | 25,706 | 29,934 |
| 25^th^ %ile | 13,882 | 14,432 | 15,741 | 8,317 | 14,148 | 15,367 | 15,512 | 19,866 |
| 10^th^ %ile | 7,384 | 7,775 | 9,419 | 2,316 | 7,723 | 9,295 | 9,358 | 13,902 |
| 5^th^ %ile | 4,567 | 4,765 | 6,804 | 522 | 4,754 | 6,781 | 6,843 | 11,010 |
| 1^st^ %ile | 1,935 | 2,003 | 3,609 | 0 | 1,987 | 3,609 | 3,717 | 7,257 |
| Std. dev. | 66,078 | 68,727 | 68,527 | 52,742 | 61,667 | 61,478 | 58,611 | 58,456 |
| Skewness | 14.15 | 13.16 | 13.25 | 20.73 | 15.26 | 15.38 | 14.23 | 14.25 |
| Kurtosis | 471.08 | 411.09 | 415.24 | 979.16 | 575.96 | 582.24 | 506.46 | 508.50 |
| Sample | 33,694 | 33,694 | 33,694 | 32,296 | 33,694 | 33,694 | 33,694 | 33,694 |
| Gini (HH-size & sampling weighted) | 52.09 (0.76) | 52.39 (0.72) | 50.77 (0.73) | 54.32 (0.81) | 50.98 (0.69) | 49.29 (0.70) | 48.63 (0.68) | 43.99 (0.67) |
| Top 0.1% inc. share | 2.89 | 2.85 | 2.79 | 3.38 | 2.71 | 2.65 | 2.54 | 2.30 |
| 0.1-1% inc. share | 8.44 | 8.36 | 8.20 | 8.96 | 7.83 | 7.67 | 7.48 | 6.82 |
| 1-5% inc. share | 17.05 | 17.27 | 16.96 | 17.66 | 16.94 | 16.62 | 16.44 | 15.18 |
| 5-10% inc. share | 12.51 | 12.64 | 12.45 | 12.70 | 12.40 | 12.20 | 12.13 | 11.45 |
| Mean log dev. (GE0) | 0.502 (0.010) | 0.509 (0.010) | 0.453 (0.009) | 0.606 (0.012) | 0.479 (0.009) | 0.424 (0.009) | 0.412 (0.008) | 0.323 (0.007) |
| Theil index (GE1) | 0.544 (0.022) | 0.547 (0.020) | 0.518 (0.020) | 0.598 (0.029) | 0.515 (0.021) | 0.485 (0.020) | 0.470 (0.019) | 0.390 (0.017) |
| Half coef. of var. squared (GE2) | 1.281 (0.216) | 1.252 (0.195) | 1.195 (0.188) | 1.671 (0.419) | 1.181 (0.224) | 1.123 (0.214) | 1.052 (0.186) | 0.855 (0.153) |

Notes: MXN0 incomes (3 household observations for market income, 714 for taxable income) are omitted in computations of the Gini. Gini standard errors are jack-knife estimates on household-level data (recognizing that household-member incomes are copies of one another), accounting for household size. Ginis and standard errors are multiplied by 100 for clarity of presentation.

Source: Own analysis of ENIGH 2012, CEQ database.

Table A2-1b. Income summary statistics, various income concepts (sampling-weighted sample)

|  | Market income, household | Taxable income, household | Gross income, household | Disposable income, household |
| --- | --- | --- | --- | --- |
| 99.9^th^ %ile | 1,106,022 | 1,066,304 | 1,106,022 | 1,106,022 |
| 99^th^ %ile | 357,572 | 254,035 | 378,584 | 324,101 |
| 95^th^ %ile | 161,906 | 116,378 | 177,564 | 160,459 |
| 90^th^ %ile | 107,522 | 74,361 | 118,779 | 108,861 |
| 75^th^ %ile | 55,715 | 37,738 | 59,881 | 56,430 |
| Mean | 51,771 | 34,830 | 56,358 | 52,298 |
| Median | 29,526 | 18,880 | 32,093 | 30,789 |
| 25^th^ %ile | 16,147 | 7,918 | 18,316 | 17,760 |
| 10^th^ %ile | 8,806 | 1,076 | 10,944 | 10,811 |
| 5^th^ %ile | 5,596 | 0 | 7,979 | 7,956 |
| 1^st^ %ile | 2,335 | 0 | 4,150 | 4,150 |
| Std. dev. | 95,550 | 80,035 | 98,428 | 89,995 |
| Skewness | 14.37 | 19.97 | 13.41 | 15.46 |
| Kurtosis | 371.22 | 668.12 | 331.30 | 443.55 |
| Sample | 8,999 | 8,288 | 9,002 | 9,002 |
| Gini (HH-size & sampling weighted) | 50.07 | 52.36 | 47.97 | 46.29 |
| Top 0.1% inc. share | 2.71 | 2.65 | 2.54 | 2.30 |
| 0.1-1% inc. share | 7.83 | 7.67 | 7.48 | 6.82 |
| 1-5% inc. share | 16.94 | 16.62 | 16.44 | 15.18 |
| 5-10% inc. share | 12.40 | 12.20 | 12.13 | 11.45 |
| Mean log dev. (GE0) | 0.465 (0.014) | 0.594 (0.017) | 0.404 (0.013) | 0.374 (0.011) |
| Theil index (GE1) | 0.475 (0.022) | 0.523 (0.025) | 0.437 (0.020) | 0.404 (0.018) |
| Half coef. of var. squared (GE2) | 0.855 (0.084) | 1.076 (0.122) | 0.784 (0.073) | 0.704 (0.067) |

Notes: MXN0 incomes (3 household observations for market income, 714 for taxable income) are omitted in computations of the Gini. Gini standard errors are jack-knife estimates on household-level data (recognizing that household-member incomes are copies of one another), accounting for household size. Ginis and standard errors are multiplied by 100 for clarity of presentation.

Source: Own analysis of ENIGH 2012, CEQ database.

Table A2-2. National Gini coefficient, various income concepts and weights

| Household income concept | Unweighted sample | Sampling weighted sample | HH size & sampling weighted sample (for HH-level income) |
| --- | --- | --- | --- |
| Market income, per capita | 52.46 (0.49) | 52.09 (0.76) | **--** |
| Taxable income, per capita | 55.17 (0.52) | 54.32 (0.81) | -- |
| Gross income, per capita | 50.41 (0.48) | 50.77 (0.73) | -- |
| Disposable income, per capita | 48.95 (0.47) | 49.29 (0.69) | -- |
| Market income, household | 50.41 (0.47) | 50.07 (0.76) | 48.39 (0.89) |
| Taxable income, household | 53.27 (0.51) | 52.36 (0.80) | 50.50 (0.88) |
| Gross income, household | 47.74 (0.47) | 47.97 (0.74) | 46.44 (0.87) |
| Disposable income, household | 46.08 (0.47) | 46.29 (0.70) | 44.79 (0.77) |

Notes: Ginis are estimated on household-level data, accounting for household size in calculations for income per capita, to recognize uncertainty due to the limited number of household-level datapoints on incomes. Different weighting schemes are used to reflect inequality at the household, household-size adjusted household, or individual level. 0-incomes (3 for market income, and 714 for taxable income) are omitted in the respective computations. Jackknife estimates of standard errors are provided. Ginis and standard errors are multiplied by 100 for clarity of presentation.

Source: Own analysis of ENIGH 2012, CEQ database.

Table A2-3. ENIGH 2012: Sample sizes, non-response rates, and mean incomes by state

| State code | State | Represented population | Strata | Primary sampling units | Fully interviewed households (individuals) | Type A non-responding HHs (%) | Mean market income  per cap. | Mean gross income  per cap. | Mean disposable income  per cap. | Mean final income  per cap. |
| --- | --- | --- | --- | --- | --- | --- | --- | --- | --- | --- |
| 01 | Aguascalientes | 1,239,249 | 4 | 36 | 266 (1,055) | 8 (2.9%) | 43,121 | 45,665 | 41,994 | 45,634 |
| 02 | Baja California | 3,341,179 | 4 | 45 | 267 (945) | 1 (0.4%) | 61,188 | 66,897 | 60,904 | 66,070 |
| 03 | Baja California Sur | 702,275 | 4 | 30 | 264 (903) | 7 (2.6%) | 61,467 | 65,027 | 58,178 | 63,088 |
| 04 | Campeche | 869,325 | 4 | 33 | 287 (1,047) | 2 (0.7%) | 45,662 | 48,497 | 44,286 | 50,268 |
| 05 | Coahuila de Zarag. | 2,860,330 | 3 | 42 | 266 (918) | 10 (3.6%) | 40,954 | 43,494 | 39,925 | 43,856 |
| 06 | Colima | 689,225 | 4 | 39 | 275 (986) | 5 (1.8%) | 43,082 | 46,149 | 42,623 | 46,962 |
| 07 | Chiapas | 5,057,829 | 4 | 21 | 296 (1,287) | 2 (0.7%) | 18,202 | 20,951 | 20,012 | 24,079 |
| 08 | Chihuahua | 3,610,120 | 3 | 42 | 266 (884) | 3 (1.1%) | 36,084 | 40,658 | 37,697 | 41,842 |
| 09 | Distrito Federal | 8,838,219 | 2 | 80 | 365 (1,222) | 3 (0.8%) | 73,955 | 79,655 | 72,716 | 74,362 |
| 10 | Durango | 1,715,230 | 4 | 30 | 272 (1,058) | 8 (2.9%) | 30,629 | 33,712 | 31,577 | 36,041 |
| 11 | Guanajuato | 5,683,512 | 4 | 27 | 266 (1,109) | 8 (2.9%) | 36,004 | 38,308 | 35,831 | 39,380 |
| 12 | Guerrero | 3,506,806 | 4 | 24 | 254 (1,016) | 3 (1.2%) | 21,879 | 24,675 | 23,619 | 28,016 |
| 13 | Hidalgo | 2,769,342 | 4 | 21 | 280 (1,077) | 5 (1.8%) | 28,920 | 31,392 | 29,781 | 33,921 |
| 14 | Jalisco | 7,662,548 | 4 | 47 | 334 (1,343) | 27 (7.5%) | 46,244 | 48,307 | 44,788 | 47,940 |
| 15 | México | 16,182,659 | 4 | 53 | 372 (1,379) | 7 (1.8%) | 43,320 | 45,164 | 41,396 | 44,246 |
| 16 | Mich. de Ocampo | 4,482,196 | 4 | 24 | 288 (1,199) | 2 (0.7%) | 27,813 | 29,964 | 28,572 | 32,425 |
| 17 | Morelos | 1,857,630 | 4 | 30 | 265 (909) | 5 (1.9%) | 37,404 | 40,748 | 38,301 | 41,497 |
| 18 | Nayarit | 1,162,206 | 4 | 27 | 281 (1,086) | 1 (0.4%) | 37,065 | 40,318 | 37,330 | 41,742 |
| 19 | Nuevo León | 4,889,962 | 4 | 47 | 243 (849) | 24 (9.0%) | 65,982 | 70,759 | 64,115 | 67,019 |
| 20 | Oaxaca | 3,930,798 | 4 | 18 | 280 (1,079) | 5 (1.8%) | 22,618 | 24,758 | 23,116 | 27,369 |
| 21 | Puebla | 6,015,009 | 4 | 27 | 275 (1,035) | 7 (2.5%) | 30,263 | 31,825 | 29,829 | 33,645 |
| 22 | Querétaro | 1,913,297 | 4 | 30 | 280 (936) | 5 (1.8%) | 59,096 | 66,816 | 62,658 | 65,282 |
| 23 | Quintana Roo | 1,451,607 | 4 | 45 | 277 (1,012) | 3 (1.1%) | 52,303 | 53,583 | 49,146 | 53,515 |
| 24 | San Luis Potosí | 2,683,229 | 4 | 30 | 263 (970) | 12 (4.4%) | 34,245 | 37,024 | 34,634 | 38,327 |
| 25 | Sinaloa | 2,913,641 | 4 | 33 | 275 (1,018) | 4 (1.4%) | 40,037 | 43,588 | 40,350 | 45,532 |
| 26 | Sonora | 2,822,119 | 4 | 42 | 261 (900) | 17 (6.1%) | 53,640 | 56,726 | 51,956 | 56,896 |
| 27 | Tabasco | 2,313,323 | 4 | 21 | 263 (984) | 7 (2.6%) | 36,428 | 38,443 | 36,156 | 40,525 |
| 28 | Tamaulipas | 3,431,876 | 4 | 45 | 243 (869) | 25 (9.3%) | 45,601 | 49,697 | 45,499 | 50,477 |
| 29 | Tlaxcala | 1,229,949 | 3 | 36 | 273 (1,115) | 2 (0.7%) | 26,362 | 27,857 | 26,681 | 30,776 |
| 30 | Veracruz de Ign. | 7,877,528 | 4 | 32 | 360 (1,370) | 9 (2.4%) | 30,720 | 33,657 | 32,115 | 36,300 |
| 31 | Yucatán | 2,041,572 | 4 | 30 | 267 (1,118) | 11 (4.0%) | 33,963 | 36,972 | 34,587 | 38,400 |
| 32 | Zacatecas | 1,540,639 | 4 | 24 | 278 (1,016) | 11 (3.8%) | 29,534 | 32,413 | 29,862 | 34,547 |
|  | Nationwide | 117,284,429 | 123 | 1,111 | 9,002 (33,694) | 249 (2.7%) | 41,290 | 44,327 | 41,025 | 44,712 |

Note: Mean incomes account for sampling weights but are computed only among responding households, and may not be representative of underlying population.

Source: Instituto Nacional de Estadística y Geografía (INEGI, 2013), Encuesta Nacional de Ingresos y Gastos de los Hogares 2012: Diseño muestral; Resultados Definitivos por Código de Resultados de Entrevista de Campo ENIGH – 2012; own analysis of ENIGH 2012, CEQ database.

Table A2-4. Estimation results for various univariate logistic models of response probability

| Specification of $g\left( x_{i},\theta\right)$ | $\hat{\theta_{0}}$ (s.e.) | $\hat{\theta_{1}}$ (s.e.) | Sum of Squared Weighted Errors | Factor of Proportio-nality (σ^2^) | AIC  SIC | Per-capita  market income, unweighted data, Gini (s.e.) | Per-capita  market income, weighted data, Gini (s.e.) | HH market income, weighted data, Gini (s.e.) |
| --- | --- | --- | --- | --- | --- | --- | --- | --- |
| Uncorrected |  |  |  |  |  | 52.46 (0.49) | 52.09 (0.76) | 48.39 (0.89) |
| 1: θ_0_+θ_1_log(market inc.) | 10.593 (.020) | -0.611 (.002) | 295,667 | 0.161 | 296.20293.59 | 52.78 (0.52) | 52.50 (0.81) | 55.02 (1.24) |
| 2: θ_0_+θ_1_log(taxable inc.) | 5.668 (0.008) | -0.316 (0.001) | 636,310 | 0.424 | 320.73318.11 | 55.27^a^ (0.56) | 54.57^a^ (0.88) | 58.34^a^ (1.54) |
| 3: θ_0_+θ_1_log(gross inc.) | 10.592 (.020) | -0.605 (.002) | 292,641 | 0.160 | 295.87293.26 | 52.76 (0.51) | 52.47 (0.80) | 54.98 (1.23) |
| 4: θ_0_+θ_1_log(mkt. inc.pc) | 8.175 (0.015) | -0.450 (0.001) | 303,484 | 0.163 | 297.04294.42 | 52.72 (0.51) | 52.41 (0.79) | 54.99 (1.25) |
| 5: θ_0_+θ_1_log(taxbl. inc.pc) | 4.578 (0.006) | -0.246 (0.001) | 650,032 | 0.428 | 321.41318.80 | 55.33^a^ (0.55) | 54.61^a^ (0.87) | 58.44^a^ (1.56) |
| 6: θ_0_+θ_1_log(gross inc.pc) | 8.150 (0.016) | -0.441 (0.002) | 299,257 | 0.161 | 296.59293.97 | 52.70 (0.51) | 52.38 (0.79) | 54.95 (1.24) |
| 7: θ_0_+θ_1_log(gross inc.)^2^ | 6.915 (0.010) | -0.025 (0.000) | 293,452 | 0.160 | 295.96293.35 | 52.80 (0.52) | 52.53 (0.81) | 55.04 (1.25) |
| 8: θ_0_+θ_1_10^-3^ gross inc. | 93.088 (0.468) | -0.030 (0.000) | 532,100 | 0.284 | 315.00312.39 | 55.84 (3.14) | 58.75 (6.33) | 68.28 (13.61) |
| 9: θ_0_+θ_1_10^-12^gross inc.^2^ | 3.714 (0.001) | -0.584 (0.000) | 285,710 | 0.153 | 295.10292.49 | 54.74 (1.41) | 55.95 (2.59) | 61.38 (5.73) |
| 10: θ_0_+θ_1_(10^-3^gross inc.)^½^ | 4.501 (0.002) | -0.079 (0.000) | 296,436 | 0.160 | 296.28293.67 | 53.30 (0.60) | 53.25 (0.96) | 55.97 (1.62) |

Note: Standard errors on Gini coefficients are jackknife estimates. ^a^ Because this sample omits observations with missing taxable income, Gini of market income would not be comparable to table 2. Gini of taxable income is reported instead.

Source: Own analysis of ENIGH 2012, CEQ database.

Table A2-5. Estimation results for selected multivariate models of response probability

| Specification of *g(x)* | $\hat{\theta_{0}}$ (s.e.) | $\hat{\theta_{1}}$ (s.e.) | $\hat{\theta_{2}}$ (s.e.) | $\hat{\theta_{3}}$ (s.e.) | $\hat{\theta_{4}}$ (s.e.) | Sum of Squared Weighted Errors | Factor of Proportio-nality (σ^2^) | AIC | Mkt. inc. per cap. weighted data, Gini (s.e.) |
| --- | --- | --- | --- | --- | --- | --- | --- | --- | --- |
| Uncorrected |  |  |  |  |  |  |  |  | 52.09 (0.76) |
| θ_0_+θ_1_10^-6^gross inc.  +θ_2_10^-12^gross inc.^2^ | 3.685 (0.002) | 0.231 (0.012) | -0.657 (0.004) |  |  | 285,673 | 0.153 | 297.10 | 55.98 (2.66) |
| θ_0_+θ_1_10^-6^gross inc.pc  +θ_2_10^-12^gross inc.pc^2^ | 3.699 (0.001) | -0.688 (0.024) | -0.440 (0.007) |  |  | 228,694 | 0.126 | 289.98 | 56.48 (4.57) |
| θ_0_+θ_1_log(gross inc.pc) +θ_2_urban+θ_3_age+θ_4_age^2^ | 51.704 (0.179) | -1.647 (0.002) | 2.162 (0.003) | -87.016 (0.534) | 55.082 (0.388) | 231,404 | 0.158 | 294.36 | 52.66 (0.88) |
| θ_0_+θ_1_log(gross inc.pc) +θ_2_sch.attend+θ_3_age+θ_4_age^2^ | 255.093 (0.868) | -0.838 (0.001) | -13.148 (0.036) | -620 (2.269) | 395.035 (1.482) | 250,534 | 0.198 | 296.90 | 51.90 (0.75) |
| θ_0_+θ_1_log(gross inc.pc) +θ_2_urban+θ_3_HH size | 7.398 (0.028) | -0.158 (0.003) | -1.887 (0.015) | -2.180 (0.007) |  | 289,632 | 0.153 | 299.54 | 51.98 (0.77) |
| θ_0_+θ_1_log(gross inc.pc) +θ_2_urb+θ_3_hsize+θ_4_hsize^2^ | 24.272 (11∙10^3^) | -0.273 (0.003) | -15.935 (11∙10^3^) | -76.587 (0.364) | 252.898 (2.049) | 277,454 | 0.152 | 300.17 | 51.85 (0.78) |
| θ_0_+θ_1_log(gross inc.pc) +θ_2_male+θ_3_hsize | 26.633 (23∙10^3^) | -0.683 (0.002) | -15.496 (23∙10^3^) | -23.586 (0.064) |  | 278,514 | 0.151 | 298.29 | 52.65 (0.85) |
| θ_0_+θ_1_log(gross inc.pc) +θ_2_male+θ_3_urban | 32.360 (27∙10^3^) | -1.194 (0.001) | -17.268 (27∙10^3^) | 1.728 (0.003) |  | 275,568 | 0.167 | 297.95 | 54.41 (1.26) |
| θ_0_+θ_1_log(gross inc.pc) +θ_2_postsecondary.attend | 8.123 (0.016) | -0.438 (0.002) | -0.265 (0.040) |  |  | 299,251 | 0.161 | 298.59 | 52.38 (0.79) |
| θ_0_+θ_1_log(gross inc.pc) +θ_2_postsecond.attend+θ_3_age | 20.778 (0.027) | -0.738 (0.002) | -4.585 (0.018) | -16.566 (0.027) |  | 265,835 | 0.167 | 296.80 | 52.12 (0.77) |

Note: Standard errors on Gini coefficients are jackknife estimates. Variables are normalized: age=(years-12)/100 ; HH size=(#-1)/100.

Source: Own analysis of ENIGH 2012, CEQ database.

Table A2-6. ENIGH 2012: Income summary statistics for various income concepts, nonresponse corrected weights

|  | Market income per cap. | Market income + pensions  per cap. | Gross income per cap. | Taxable income per cap. | Net market income per cap. | Disposable income per cap. | Consumable income per cap. | Final income per cap. |
| --- | --- | --- | --- | --- | --- | --- | --- | --- |
| 99.9^th^ %ile | 656,327 | 642,581 | 642,581 | 515,538 | 536,234 | 536,234 | 524,765 | 526,396 |
| 99^th^ %ile | 281,576 | 315,599 | 315,599 | 209,259 | 272,727 | 272,727 | 260,226 | 262,465 |
| 95^th^ %ile | 133,423 | 141,873 | 142,222 | 95,663 | 127,003 | 127,170 | 124,157 | 127,759 |
| 90^th^ %ile | 86,077 | 90,777 | 91,450 | 61,967 | 83,688 | 84,098 | 82,499 | 86,346 |
| 75^th^ %ile | 46,670 | 49,318 | 49,738 | 33,197 | 45,473 | 45,843 | 45,485 | 50,035 |
| Mean | 42,309 | 44,432 | 45,322 | 29,618 | 40,992 | 41,882 | 41,234 | 45,536 |
| Median | 25,552 | 26,631 | 27,349 | 17,935 | 25,466 | 25,967 | 25,944 | 30,219 |
| 25^th^ %ile | 14,094 | 14,614 | 15,927 | 8,476 | 14,313 | 15,536 | 15,636 | 20,035 |
| 10^th^ %ile | 7,506 | 7,881 | 9,467 | 2,400 | 7,820 | 9,358 | 9,402 | 13,957 |
| 5^th^ %ile | 4,652 | 4,812 | 6,898 | 538 | 4,806 | 6,876 | 6,952 | 11,061 |
| 1^st^ %ile | 1,984 | 2,003 | 3,703 | 0 | 2,003 | 3,703 | 3,785 | 7,340 |
| Std. dev. | 69,207 | 71,747 | 71,550 | 55,500 | 64,366 | 64,180 | 61,114 | 60,950 |
| Skewness | 14.27 | 13.32 | 13.40 | 20.83 | 15.52 | 15.63 | 14.49 | 14.51 |
| Kurtosis | 462.14 | 406.97 | 410.82 | 947.45 | 572.59 | 578.43 | 504.98 | 507.11 |
| Sample | 33,686 | 33,686 | 33,686 | 33,686 | 33,686 | 33,686 | 33,686 | 33,686 |
| Top 0.1% inc. share | 3.02 | 2.97 | 2.91 | 3.53 | 2.84 | 2.78 | 2.66 | 2.41 |
| 0.1-1% inc. share | 8.60 | 8.47 | 8.30 | 9.13 | 7.90 | 7.74 | 7.56 | 6.90 |
| 1-5% inc. share | 17.17 | 17.41 | 17.11 | 17.77 | 17.08 | 16.77 | 16.58 | 15.33 |
| 5-10% inc. share | 12.56 | 12.70 | 12.52 | 12.73 | 12.44 | 12.25 | 12.19 | 11.51 |
| Gini (HH-size & sampling weighted data) | 52.50 (0.81) | 52.76 (0.77) | 51.19 (0.78) | 54.70 (0.87) | 51.33 (0.74) | 49.68 (0.74) | 49.02 (0.73) | 44.42 (0.72) |
| Gini (HH-size wted, no sampling wght) | 52.78 (0.52) | 53.07 (0.51) | 50.74 (0.51) | 55.44 (0.56) | 51.70 (0.50) | 49.27 (0.50) | 48.64 (0.49) | 43.50 (0.48) |
| Gini (equal HH weights = unweighted) | 54.89 (0.74) | 55.02 (0.71) | 53.02 (0.72) | 58.59 (0.84) | 53.72 (0.70) | 51.64 (0.71) | 50.93 (0.69) | 46.88 (0.69) |
| Mean log dev. (GE0) | 0.510 (0.010) | 0.516 (0.010) | 0.461 (0.010) | 0.613 (0.013) | 0.485 (0.010) | 0.431 (0.010) | 0.419 (0.009) | 0.329 (0.008) |
| Theil index (GE1) | 0.556 (0.024) | 0.558 (0.023) | 0.529 (0.022) | 0.611 (0.033) | 0.525 (0.023) | 0.496 (0.023) | 0.480 (0.021) | 0.399 (0.019) |
| Half coef. of var. squared (GE2) | 1.338 (0.242) | 1.304 (0.219) | 1.246 (0.211) | 1.756 (0.470) | 1.233 (0.253) | 1.174 (0.243) | 1.098 (0.211) | 0.896 (0.174) |

Notes: Statistics are based on non-response correction weights estimated in the logarithmic model of market income (model 1). These statistics exclude 3 household observations with market income of 0. The statistics are still comparable to those in table 1, which are extremely robust to this exclusion (changing by 0.01 at most.) (Another 714 household observations are omitted in computations of the Gini for taxable income.) Gini standard errors are jack-knife estimates on household-level data (recognizing that household-member incomes are copies of one another), accounting for household size except in last row. Ginis and standard errors are multiplied by 100 for clarity of presentation.

Source: Own analysis of ENIGH 2012, CEQ database.

Table A2-7. Distribution of top incomes, various income concepts: Pareto (type I) estimates

| Cutoff percentile | Market income per cap. | Taxable income per cap. | Gross income per cap. | Disposable income per cap. | Market income, household | Taxable income, household | Gross income, household | Disposable income, household |
| --- | --- | --- | --- | --- | --- | --- | --- | --- |
| Pareto (type I) coefficient $\alpha$ | | |  |  |  |  |  |  |
| Top 25% | 1.63 (.04) | 1.61 (.04) | 1.64 (.04) | 1.67 (.04) | 1.87 (.06) | 1.87 (.06) | 1.84 (.06) | 1.92 (.06) |
| Top 10% | 1.83 (.07) | 1.81 (.07) | 1.84 (.07) | 1.89 (.07) | 2.10 (.11) | 2.11 (.11) | 2.20 (.12) | 2.21 (.11) |
| Top 5% | 2.14 (.13) | 2.11 (.13) | 2.22 (.13) | 2.18 (.12) | 2.31 (.20) | 2.37 (.21) | 2.39 (.22) | 2.54 (.22) |
| Top 1% | 2.31 (.23) | 2.35 (.27) | 2.53 (.27) | 2.74 (.40) | 2.44 (.40) | 1.96 (.25) | 2.28 (.38) | 2.46 (.36) |
| Top 0.1% | 1.74 (.43) | 1.76 (.44) | 1.52 (.26) | 1.67 (.35) | 11.58 (6.0) | 5.57 (3.41) | 12.42 (4.9) | 4.48 (1.41) |
|  |  |  |  |  |  |  |  |  |
| Inverted Pareto coefficient $\beta=\alpha/\left[ \alpha-1 \right]$ | | | | |  |  |  |  |
| Top 25% | 2.59 | 2.64 | 2.56 | 2.49 | 2.15 | 2.15 | 2.19 | 2.09 |
| Top 10% | 2.20 | 2.23 | 2.19 | 2.12 | 1.91 | 1.90 | 1.83 | 1.83 |
| Top 5% | 1.88 | 1.90 | 1.82 | 1.85 | 1.76 | 1.73 | 1.72 | 1.65 |
| Top 1% | 1.76 | 1.74 | 1.65 | 1.57 | 1.69 | 2.04 | 1.78 | 1.68 |
| Top 0.1% | 2.35 | 2.32 | 2.92 | 2.49 | 1.09 | 1.22 | 1.09 | 1.29 |
|  |  |  |  |  |  |  |  |  |
| Top income share (%) | |  |  |  |  |  |  |  |
| Top 25% | 65.96 | 68.35 | 65.04 | 63.81 | 61.12 | 63.53 | 60.49 | 58.76 |
| Top 10% | 43.15 | 44.75 | 42.66 | 41.16 | 37.57 | 38.92 | 36.49 | 35.32 |
| Top 5% | 28.99 | 30.43 | 28.45 | 27.61 | 24.95 | 25.79 | 24.10 | 22.78 |
| Top 1% | 11.46 | 12.25 | 11.05 | 10.03 | 9.89 | 11.34 | 9.57 | 8.85 |
| Top 0.1% | 3.39 | 3.62 | 3.51 | 3.03 | 0.52 | 0.63 | 0.59 | 0.87 |
|  |  |  |  |  |  |  |  |  |
| Gini among top incomes (×100) | | |  |  |  |  |  |  |
| Top 25% | 44.30 | 45.18 | 43.71 | 42.91 | 36.54 | 36.55 | 37.39 | 35.28 |
| Top 10% | 37.62 | 38.21 | 37.19 | 36.00 | 31.32 | 30.99 | 29.33 | 29.18 |
| Top 5% | 30.49 | 31.14 | 29.07 | 29.79 | 27.56 | 26.78 | 26.44 | 24.55 |
| Top 1% | 27.59 | 26.99 | 24.65 | 22.27 | 25.82 | 34.19 | 28.01 | 25.57 |
| Top 0.1% | 40.30 | 39.54 | 49.08 | 42.88 | 4.51 | 9.86 | 4.19 | 12.57 |
|  |  |  |  |  |  |  |  |  |
| Half coefficient of variation squared (GE2)^i^ | | | |  |  |  |  |  |
| Top 10% | -- | -- | -- | -- | 1.44 | 0.94 | 0.51 | 0.46 |
| Top 5% | 0.84 | 1.41 | 0.48 | 0.56 | 0.33 | 0.24 | 0.21 | 0.13 |
| Top 1% | 0.22 | 0.26 | 0.16 | 0.09 | 0.20 | -- | 0.24 | 0.17 |
| Top 0.1% | -- | -- | -- | -- | 0.0000 | 0.0002 | 0.0014 | 0.0004 |
|  |  |  |  |  |  |  |  |  |
| Minimum income | |  |  |  |  |  |  |  |
| Top 25% | 45,967 | 32,772 | 49,088 | 45,367 | 194,623 | 144,590 | 201,836 | 188,979 |
| Top 10% | 84,011 | 59,957 | 90,000 | 82,279 | 324,495 | 243,256 | 349,393 | 315,991 |
| Top 5% | 128,837 | 92,348 | 139,344 | 124,248 | 462,288 | 349,826 | 491,296 | 447,836 |
| Top 1% | 274,420 | 203,607 | 295,241 | 264,017 | 955,536 | 661,967 | 951,337 | 850,260 |
| Top 0.1% | 610,435 | 515,538 | 642,581 | 536,234 | 2,682,735 | 1,933,700 | 2,650,090 | 1,992,925 |
|  |  |  |  |  |  |  |  |  |
| Mean income | |  |  |  |  |  |  |  |
| Top 25% | 118,759 | 86,809 | 125,432 | 113,404 | 417,055 | 310,181 | 443,812 | 395,176 |
| Top 10% | 185,464 | 133,920 | 196,722 | 174,445 | 624,850 | 460,674 | 639,892 | 574,836 |
| Top 5% | 242,700 | 177,718 | 254,243 | 229,045 | 821,342 | 606,089 | 838,281 | 731,199 |
| Top 1% | 470,864 | 354,848 | 491,538 | 417,642 | 1,626,169 | 1375,461 | 1,645,154 | 1,424,930 |
| Top 0.1% | 1,410,079 | 1,194,779 | 1,453,842 | 1,194,596 | 2,766,433 | 2,077,948 | 3,036,647 | 2,186,562 |
|  |  |  |  |  |  |  |  |  |

^i^ Unable to calculate for other top income groups.

Notes: Pareto robust standard errors reported.

Source: Own analysis of ENIGH 2012, CEQ database.

Table A2-8. Replacement of top incomes with Pareto I distribution: uncorrected vs. corrected Ginis

| Cutoff percentile | Market income per cap. | Market inc + pensions  per cap. | Gross income per cap. | Taxable income per cap. | Net market income per cap. | Disposable income per cap. | Consumable income per cap. | Final income per cap. |
| --- | --- | --- | --- | --- | --- | --- | --- | --- |
| Nonparametric Gini among bottom incomes | | | |  |  |  |  |  |
| Top 25% | 31.55 (0.34) | 31.85 (0.35) | 29.25 (0.31) | 34.95 (0.41) | 31.06 (0.34) | 28.43 (0.31) | 28.06 (0.30) | 23.56 (0.25) |
| Top 10% | 37.53 (0.33) | 37.83 (0.33) | 35.73 (0.31) | 40.16 (0.38) | 36.86 (0.33) | 34.73 (0.30) | 34.27 (0.30) | 29.86 (0.27) |
| Top 5% | 41.63 (0.37) | 41.98 (0.38) | 40.09 (0.37) | 43.80 (0.39) | 40.85 (0.37) | 38.92 (0.35) | 38.41 (0.35) | 33.92 (0.33) |
| Top 1% | 47.58 (0.46) | 47.97 (0.45) | 46.25 (0.45) | 49.56 (0.47) | 46.81 (0.45) | 45.03 (0.44) | 44.63 (0.48) | 39.90 (0.42) |
| Top 0.1% | 50.83 (0.66) | 51.26 (0.64) | 49.62 (0.64) | 52.97 (0.68) | 49.84 (0.59) | 48.13 (0.60) | 47.52 (0.59) | 42.89 (0.58) |
|  |  |  |  |  |  |  |  |  |
| Nonparametric Gini among top incomes | | | |  |  |  |  |  |
| Top 25% | 35.02 (1.22) | 35.10 (1.15) | 34.96 (1.15) | 35.80 (1.37) | 34.07 (1.10) | 33.94 (1.10) | 33.45 (1.06) | 31.80 (1.05) |
| Top 10% | 30.17 (1.64) | 29.80 (1.53) | 29.69 (1.52) | 31.62 (1.87) | 29.05 (1.51) | 28.92 (1.51) | 28.46 (1.46) | 27.60 (1.44) |
| Top 5% | 27.72 (2.04) | 27.09 (1.92) | 27.03 (1.92) | 29.33 (2.40) | 26.33 (2.00) | 26.23 (2.00) | 25.77 (1.93) | 25.27 (1.91) |
| Top 1% | 24.48 (4.31) | 23.23 (4.12) | 23.21 (4.11) | 27.37 (4.98) | 24.38 (4.31) | 24.33 (4.30) | 24.26 (4.22) | 23.95 (4.09) |
| Top 0.1% | 24.51 (9.14) | 20.32 (9.00) | 20.32 (9.00) | 27.24 (12.77) | 25.41 (10.50) | 25.40 (10.50) | 24.78 (10.11) | 24.71 (10.09) |
|  |  |  |  |  |  |  |  |  |
| Pareto (type I) coefficient | | |  |  |  |  |  |  |
| Top 25% | 1.63 (.04) | 1.63 (0.04) | 1.64 (0.04) | 1.61 (0.04) | 1.66 (0.04) | 1.67 (0.04) | 1.68 (0.04) | 1.78 (0.05) |
| Top 10% | 1.83 (.07) | 1.82 (0.07) | 1.84 (0.07) | 1.81 (0.07) | 1.90 (0.07) | 1.89 (0.07) | 1.91 (0.07) | 1.97 (0.07) |
| Top 5% | 2.14 (.13) | 2.17 (0.13) | 2.22 (0.13) | 2.11 (0.13) | 2.19 (0.12) | 2.18 (0.12) | 2.21 (0.12) | 2.30 (0.12) |
| Top 1% | 2.31 (.23) | 2.49 (0.27) | 2.53 (0.27) | 2.35 (0.27) | 2.74 (0.40) | 2.74 (0.40) | 2.73 (0.38) | 2.91 (0.45) |
| Top 0.1% | 1.74 (.43) | 1.52 (0.26) | 1.52 (0.26) | 1.76 (0.44) | 1.67 (0.35) | 1.67 (0.35) | 1.61 (0.32) | 1.62 (0.32) |
|  |  |  |  |  |  |  |  |  |
| Parametric Gini among top incomes | | |  |  |  |  |  |  |
| Top 25% | 44.30 | 44.26 | 43.71 | 45.18 | 43.15 | 42.91 | 42.27 | 39.05 |
| Top 10% | 37.62 | 37.99 | 37.19 | 38.21 | 35.72 | 36.00 | 35.37 | 33.92 |
| Top 5% | 30.49 | 29.97 | 29.07 | 31.14 | 29.64 | 29.79 | 29.28 | 27.79 |
| Top 1% | 27.59 | 25.12 | 24.65 | 26.99 | 22.32 | 22.27 | 22.39 | 20.73 |
| Top 0.1% | 40.30 | 49.08 | 49.08 | 39.54 | 42.87 | 42.88 | 45.03 | 44.79 |
|  |  |  |  |  |  |  |  |  |
| Semiparametric Gini | | |  |  |  |  |  |  |
| Top 25% | 56.32 | 56.58 | 54.83 | 59.39 | 55.07 | 53.38 | 52.63 | 47.22 |
| Top 10% | 53.99 | 54.47 | 52.69 | 56.45 | 52.62 | 51.04 | 50.32 | 45.50 |
| Top 5% | 52.53 | 52.87 | 51.11 | 54.86 | 51.47 | 49.81 | 49.14 | 44.35 |
| Top 1% | 52.23 | 52.45 | 50.81 | 54.34 | 50.87 | 49.18 | 48.55 | 43.85 |
| Top 0.1% | 52.35 | 52.88 | 51.27 | 54.54 | 51.27 | 49.57 | 48.97 | 44.32 |
|  |  |  |  |  |  |  |  |  |
| Uncorrected | 52.09 | 52.39 | 50.77 | 54.32 | 50.98 | 49.29 | 48.63 | 43.99 |
| Gini correction | | |  |  |  |  |  |  |
| Top 25% | +4.23 | +2.26 | +4.06 | +10.10 | +5.00 | +1.02 | +4.66 | +0.93 |
| Top 10% | +1.90 | +0.15 | +1.92 | +7.16 | +2.55 | –1.32 | +2.35 | –0.79 |
| Top 5% | +0.44 | –1.45 | +0.34 | +5.57 | +1.40 | –2.55 | +1.17 | –1.94 |
| Top 1% | +0.14 | –1.87 | +0.04 | +5.05 | +0.80 | –3.18 | +0.58 | –2.44 |
| Top 0.1% | +0.26 | –1.44 | +0.50 | +5.25 | +1.20 | –2.79 | +1.00 | –1.97 |
|  |  |  |  |  |  |  |  |  |

Source: Own analysis of ENIGH 2012, CEQ database.

Table A2-9. Income summary statistics: Replacing top net market incomes with Pareto I estimates, and imputing other income concepts by CEQ Method

|  | Market income per cap. | Market inc +pensions  per cap. | Gross income per cap. | Taxable income per cap. | Net market income per cap. | Disposable income per cap. | Consumable income per cap. | Final income per cap. |
| --- | --- | --- | --- | --- | --- | --- | --- | --- |
| 99.9^th^ %ile | 1,018,660 | 1,018,660 | 1,018,660 | 805,485 | 917,787 | 917,787 | 865,873 | 868,318 |
| 99^th^ %ile | 297,023 | 329,833 | 329,833 | 225,028 | 292,123 | 292,123 | 288,573 | 288,573 |
| 95^th^ %ile | 125,060 | 129,735 | 130,308 | 89,033 | 115,243 | 115,936 | 113,154 | 118,098 |
| 90^th^ %ile | 83,247 | 88,376 | 88,825 | 59,825 | 82148 | 82,293 | 80,812 | 84,701 |
| 75^th^ %ile | 45,803 | 48,508 | 49,088 | 32,772 | 44992 | 45,367 | 44,792 | 49,133 |
| Mean | 42,591 | 44,730 | 45,628 | 30,187 | 41,427 | 42,326 | 41,619 | 45,922 |
| Median | 25,232 | 26,253 | 27,012 | 17,667 | 25,196 | 25,768 | 25,706 | 29,934 |
| 25^th^ %ile | 13,865 | 14,432 | 15,741 | 8,354 | 14,148 | 15,367 | 15,512 | 19,866 |
| 10^th^ %ile | 7,364 | 7,775 | 9,419 | 2,313 | 7,723 | 9,295 | 9,358 | 13,902 |
| 5^th^ %ile | 4,552 | 4,765 | 6,804 | 492 | 4,754 | 6,781 | 6,843 | 11,010 |
| 1^st^ %ile | 1,935 | 2,003 | 3,609 | 0 | 1,987 | 3,609 | 3,713 | 7,257 |
| Std. dev. | 102,090 | 104,859 | 104,718 | 91,988 | 99,416 | 99,288 | 92,713 | 92,575 |
| Skewness | 43.11 | 40.50 | 40.64 | 55.99 | 46.28 | 46.44 | 43.84 | 43.94 |
| Kurtosis | 3,191.63 | 2,881.43 | 2,895.64 | 4,732.05 | 3,548.63 | 3,565.36 | 3,270.63 | 3,282.45 |
| Sample | 33,694 | 33,694 | 33,694 | 32,296 | 33,694 | 33,694 | 33,694 | 33,694 |
| Gini (HH-size & sampling weighted) | 53.68 (1.07) | 53.85 (1.03) | 52.25 (1.04) | 56.37 (1.27) | 52.60 (1.06) | 50.93 (1.07) | 50.20 (1.03) | 45.54 (1.02) |
| Top 0.1% inc. share | 4.61 | 4.53 | 4.45 | 5.72 | 4.53 | 4.43 | 4.22 | 3.83 |
| 0.1-1% inc. share | 10.22 | 10.20 | 10.005 | 11.32 | 9.86 | 9.65 | 9.39 | 8.56 |
| 1-5% inc. share | 16.14 | 16.23 | 15.96 | 16.35 | 15.80 | 15.53 | 15.40 | 14.28 |
| 5-10% inc. share | 11.84 | 11.87 | 11.70 | 11.89 | 11.58 | 11.40 | 11.37 | 10.77 |
| Mean log dev. (GE0) | 0.535 (0.018) | 0.539 (0.017) | 0.483 (0.017) | 0.651 (0.024) | 0.512 (0.018) | 0.457 (0.017) | 0.442 (0.016) | 0.350 (0.014) |
| Theil index (GE1) | 0.645 (0.057) | 0.644 (0.053) | 0.613 (0.053) | 0.741 (0.082) | 0.621 (0.058) | 0.589 (0.057) | 0.567 (0.053) | 0.477 (0.048) |
| Half coef. of var. squared (GE2) | 2.873 (1.320) | 2.748 (0.196) | 2.633 (1.151) | 4.643 (2.578) | 2.879 (1.396) | 2.751 (1.339) | 2.481 (1.158) | 2.032 (0.956) |

Notes: MXN0 incomes (3 household observations for market income, 714 for taxable income) are omitted in computations of the Gini. Gini standard errors are jack-knife estimates on household-level data (recognizing that household-member incomes are copies of one another), accounting for household size. Ginis and standard errors are multiplied by 100 for clarity of presentation.

Source: Own analysis of ENIGH 2012, CEQ database.

Table A2-10. Summary results of correction methods: corrected Ginis for all income concepts

|  | Market income per cap. | Market inc.+ pensions  per cap. | Gross income per cap. | Taxable income per cap. | Net market income per cap. | Disposable income per cap. | Consumable income per cap. | Final income per cap. |
| --- | --- | --- | --- | --- | --- | --- | --- | --- |
| Uncorrected | 52.1 | 52.4 | 50.8 | 54.3 | 51.0 | 49.3 | 48.6 | 44.0 |
|  |  |  |  |  |  |  |  |  |
| Correction by reweighting (models in table A2-4) | | | | | | | | |
| Minimum | 52.4 **+0.3** | 52.7 **+0.3** | 51.1 **+0.3** | 54.6 **+0.3** | 51.1 **+0.1** | 49.6 **+0.3** | 48.9 **+0.3** | 44.3 **+0.3** |
| Mean | 53.2 **+1.1** | 53.5 **+1.1** | 51.9 **+1.1** | 55.7 **+1.4** | 52.1 **+1.1** | 50.5 **+1.2** | 49.8 **+1.2** | 45.2 **+1.2** |
| Median | 52.4 **+0.3** | 52.7 **+0.3** | 51.2 **+0.4** | 54.7 **+0.4** | 51.3 **+0.3** | 49.6 **+0.3** | 49.0 **+0.4** | 44.4 **+0.4** |
| Max | 58.7 **+6.6** | 58.7 **+6.3** | 57.2 **+6.4** | 62.7 **+8.4** | 57.9 **+6.9** | 56.3 **+7.0** | 55.3 **+6.7** | 50.7 **+6.7** |
|  |  |  |  |  |  |  |  |  |
| Correction by Pareto (type I) replacing of own income concept | | | | | | | | |
| Minimum | 52.2 **+0.1** | 52.5 **+0.1** | 50.8 **+0.0** | 54.3 **+0.0** | 50.9 **–0.1** | 49.2 **–0.1** | 48.6 **+0.0** | 43.9 **–0.1** |
| Mean | 53.5 **+1.4** | 53.9 **+1.5** | 52.1 **+1.4** | 55.9 **+1.6** | 52.3 **+1.3** | 50.6 **+1.3** | 49.9 **+1.3** | 45.0 **+1.0** |
| Median | 52.5 **+0.4** | 52.9 **+0.5** | 51.3 **+0.5** | 54.9 **+0.6** | 51.5 **+0.5** | 49.8 **+0.5** | 49.1 **+0.5** | 44.4 **+0.4** |
| Max | 56.3 **+4.2** | 56.6 **+4.2** | 54.8 **+4.1** | 59.4 **+5.1** | 55.1 **+4.1** | 53.4 **+4.1** | 52.6 **+4.0** | 47.2 **+3.2** |
|  |  |  |  |  |  |  |  |  |
| Correction by Pareto (type I) replacing of market income + CEQ Method | | | | | | | | |
| Minimum | 52.0 **–0.1** | 52.3 **–0.1** | 50.6 **–0.1** | 54.2 **–0.1** | 50.8 **–0.1** | 49.1 **–0.2** | 48.5 **–0.2** | 43.8 **–0.1** |
| Mean | 53.3 **+1.2** | 53.5 **+1.1** | 51.9 **+1.1** | 55.9 **+1.6** | 52.2 **+1.2** | 50.5 **+1.2** | 49.8 **+1.2** | 45.1 **+1.1** |
| Median | 52.5 **+0.4** | 52.8 **+0.4** | 51.2 **+0.4** | 54.9 **+0.6** | 51.4 **+0.5** | 49.7 **+0.5** | 49.1 **+0.4** | 44.4 **+0.4** |
| Max | 55.9 **+3.8** | 55.9 **+3.5** | 54.3 **+3.5** | 59.4 **+5.0** | 54.8 **+3.8** | 53.1 **+3.9** | 52.2 **+3.6** | 47.5 **+3.5** |
|  |  |  |  |  |  |  |  |  |

Notes: These Ginis are comparable to ‘Gini (HH-size & sampling weighted data)’ in tables A2-1, A2-6 and A2-9, and ‘Semiparametric Gini’ in table A2-8. Ginis, and differences in them, are multiplied by 100 for clarity of presentation.

Source: Own analysis of ENIGH 2012, CEQ database.

Table A2-11. Redistributive impacts of fiscal tools: high/center/low estimates

|  |  | +  Net contribut. pensions | +  Cash-like transfers | +  Nontaxable income | –  Direct taxes | –  Indirect taxes & subsidies | +  Net in-kind programs | |  | |
| --- | --- | --- | --- | --- | --- | --- | --- | --- | --- | --- |
|  | Market income  inequality | Market → Market+Pensions | Market+Pensions → Gross | Taxable →  Gross | Gross → Disposable^i^ | Disposable → Consumable | Consumable → Final | | Final income  inequality | |
| ***Gini coefficient: pc.pt. change*** | | | | | | | |  | |  |
| Uncorrected 52.09 | | +0.18 | –1.60 | –4.12 | –1.32 | –0.72 | –4.66 | | 43.09 | |
|  |  |  |  |  |  |  |  | |  | |
| Income distrib. corrected for nonresponse by reweighting | | | | | | | |  | |  |
| High | 52.57 | +0.26 | –1.58 | –3.53 | –1.51 | –0.66 | –4.60 | | 44.48 | |
| Center | 52.50 | +0.26 | –1.57 | –3.51 | –1.51 | –0.66 | –4.60 | | 44.42 | |
| Low | 52.41 | +0.28 | –1.59 | –3.53 | –1.49 | –0.66 | –4.60 | | 44.34 | |
|  |  |  |  |  |  |  |  | |  | |
| Each income concept corrected for top income mismeasurement by replacing | | | | | | | |  | |  |
| High | 56.32 | +0.26 | –1.75 | –4.56 | –1.45 | –0.75 | –5.41 | | 47.22 | |
| Center | 53.99 | +0.48 | –1.78 | –3.76 | –1.65 | –0.72 | –4.82 | | 45.54 | |
| Low | 52.23 | +0.22 | –1.64 | –3.53 | –1.63 | –0.63 | –4.70 | | 43.85 | |
|  |  |  |  |  |  |  |  | |  | |
| Market income corrected for top income biases by replacing + CEQ Method | | | | | | | |  | |  |
| High | 55.89 | –0.03 | –1.59 | –5.08 | –1.13 | –0.90 | –4.71 | | 47.54 | |
| Center | 53.68 | +0.18 | –1.60 | –4.12 | –1.32 | –0.72 | –4.66 | | 45.54 | |
| Low | 51.96 | +0.30 | –1.62 | –3.53 | –1.50 | –0.66 | –4.64 | | 43.84 | |
|  |  |  |  |  |  |  |  | |  | |
| ***Top 10 percent income share: pc.pt. change*** | | | | | | | |  | |  |
| Uncorrected 40.89 | | +0.23 | –0.72 | –2.30 | –1.26 | –0.55 | –2.84 | | 35.75 | |
|  |  |  |  |  |  |  |  | |  | |
| Income distrib. corrected for nonresponse by reweighting | | | | | | | |  | |  |
| High | 41.43 | +0.19 | –0.70 | –2.32 | –1.31 | –0.55 | –2.85 | | 36.21 | |
| Center | 41.35 | +0.20 | –0.71 | –2.32 | –1.30 | –0.55 | –2.84 | | 36.15 | |
| Low | 41.25 | +0.20 | –0.70 | –2.33 | –1.29 | –0.54 | –2.85 | | 36.07 | |
|  |  |  |  |  |  |  |  | |  | |
| Each income concept corrected for top income mismeasurement by replacing | | | | | | | |  | |  |
| High | 47.13 | +0.23 | –0.84 | –2.43 | –1.34 | –0.65 | –4.31 | | 40.22 | |
| Center | 43.15 | +0.46 | –0.95 | –2.08 | –1.51 | –0.61 | –3.15 | | 37.41 | |
| Low | 37.75 | –1.01 | –0.69 | –2.60 | –1.93 | –0.19 | –2.94 | | 30.99 | |
|  |  |  |  |  |  |  |  | |  | |
| Market income corrected for top income biases by replacing + CEQ Method | | | | | | | |  | |  |
| High | 45.93 | –0.06 | –0.75 | –4.29 | –0.85 | –0.86 | –3.12 | | 40.29 | |
| Center | 42.81 | +0.02 | –0.71 | –3.17 | –1.11 | –0.63 | –2.94 | | 37.44 | |
| Low | 40.74 | +0.21 | –0.70 | –2.25 | –1.29 | –0.55 | –2.85 | | 35.56 | |
|  |  |  |  |  |  |  |  | |  | |
| ***Top 1 percent income share: pc.pt. change*** | | | | | | | |  | |  |
| Uncorrected 11.33 | | –0.12 | –0.22 | –1.35 | –0.67 | –0.30 | –0.90 | | 9.12 | |
|  |  |  |  |  |  |  |  | |  | |
| Income distrib. corrected for nonresponse by reweighting | | | | | | | |  | |  |
| High | 11.68 | –0.19 | –0.22 | –1.45 | –0.70 | –0.31 | –0.91 | | 9.35 | |
| Center | 11.62 | –0.18 | –0.23 | –1.45 | –0.69 | –0.30 | –0.91 | | 9.31 | |
| Low | 11.59 | –0.17 | –0.22 | –1.43 | –0.69 | –0.30 | –0.91 | | 9.30 | |
|  |  |  |  |  |  |  |  | |  | |
| Each income concept corrected for top income mismeasurement by replacing | | | | | | | |  | |  |
| High | 15.99 | +0.45 | –0.35 | –1.44 | –1.03 | –1.14 | –1.34 | | 12.58 | |
| Center | 13.95 | +0.52 | –0.37 | –1.13 | –1.01 | –0.99 | –0.75 | | 11.33 | |
| Low | 11.46 | –0.19 | –0.21 | –1.20 | –1.03 | –0.65 | –0.62 | | 8.77 | |
|  |  |  |  |  |  |  |  | |  | |
| Market income corrected for top income biases by replacing + CEQ Method | | | | | | | |  | |  |
| High | 19.89 | –0.43 | –0.36 | –4.88 | –0.02 | –0.84 | –1.60 | | 16.64 | |
| Center | 14.83 | –0.10 | –0.27 | –2.59 | –0.38 | –0.47 | –1.22 | | 12.39 | |
| Low | 11.16 | –0.20 | –0.21 | –1.40 | –0.70 | –0.30 | –0.89 | | 8.86 | |
|  |  |  |  |  |  |  |  | |  | |

^i^ Alternatively, this can be obtained as ‘market+pensions → net market’ for estimates within 0.1 pc.pt. of those above.

Notes: These Ginis are comparable to ‘Gini (HH-size & sampling weighted data)’ in tables 1 & 3, and ‘Semiparametric Gini’ in table 4.

Source: Own analysis of ENIGH 2012, CEQ database.

Table A2-12. Generalized Pareto (type II) results, various income concepts (individual sampling-weighted sample)

| Cutoff percentile | Market income per cap. | Taxable income per cap. | Gross income per cap. | | Disposable income per cap. | Market income, household | Taxable income, household | Gross income, household | Disposable income, household |
| --- | --- | --- | --- | --- | --- | --- | --- | --- | --- |
| Pareto (type II) shape coefficient ξ | | | | |  |  |  |  |  |
| Top 25% | 0.44 (.02) | 0.43 (.02) | 0.45 (.02) | | 0.42 (.02) | 0.36 (.05) | 0.37 (.05) | 0.33 (.05) | 0.32 (.05) |
| Top 10% | 0.33 (.04) | 0.37 (.04) | 0.32 (.04) | | 0.31 (.04) | 0.34 (.07) | 0.38 (.07) | 0.36 (.07) | 0.31 (.06) |
| Top 5% | 0.37 (.05) | 0.41 (.05) | 0.39 (.06) | | 0.32 (.05) | 0.41 (.11) | 0.47 (.09) | 0.45 (.12) | 0.48 (.11) |
| Top 1% | 0.34 (.11) | 0.44 (.12) | 0.32 (.09) | | 0.45 (.12) | 0.01 (.33) | -0.15 (.15) | -0.01 (.45) | -0.05 (.28) |
| Top 0.1% | 0.10 (.22) | 0.25 (.25) | 0.07 (.21) | | 0.27 (.34) | --^i^ | -- | -- | -- |
|  |  |  |  | |  |  |  |  |  |
| Pareto (type II) scale coefficient, log(σ) | | | | |  |  |  |  |  |
| Top 25% | 10.42 (.03) | 10.10 (.03) | 10.45 (.03) | | 10.39 (.03) | 11.71 (.05) | 11.42 (.06) | 11.81 (.05) | 11.69 (.05) |
| Top 10% | 10.94 (.04) | 10.58 (.04) | 11.01 (.04) | | 10.89 (.04) | 12.09 (.08) | 11.75 (.08) | 12.06 (.08) | 12.00 (.08) |
| Top 5% | 11.10 (.06) | 10.77 (.07) | 11.11 (.07) | | 11.08 (.06) | 12.24 (.16) | 11.86 (.13) | 12.19 (.18) | 11.97 (.16) |
| Top 1% | 11.72 (.11) | 11.36 (.15) | 11.76 (.09) | | 11.40 (.15) | 13.26 (.50) | 13.33 (.28) | 13.32 (.61) | 13.17 (.42) |
| Top 0.1% | 13.17 (.33) | 12.88 (.32) | 13.23 (.33) | | 12.86 (.43) | -- | -- | -- | -- |
|  |  |  |  | |  |  |  |  |  |
| 1/ξ | | |  | |  |  |  |  |  |
| Top 25% | 2.29 | 2.30 | 2.20 | | 2.39 | 2.74 | 2.73 | 3.03 | 3.13 |
| Top 10% | 3.05 | 2.71 | 3.14 | | 3.26 | 2.96 | 2.66 | 2.76 | 3.28 |
| Top 5% | 2.67 | 2.44 | 2.56 | | 3.13 | 2.43 | 2.13 | 2.22 | 2.10 |
| Top 1% | 2.92 | 2.28 | 3.16 | | 2.24 | 67.59 | -6.71 | -99.93 | -19.90 |
| Top 0.1% | 9.77 | 4.06 | 15.38 | | 3.72 | -- | -- | -- | -- |
|  |  |  |  | |  |  |  |  |  |
| Inverted Pareto coefficient | | |  | |  |  |  |  |  |
| Top 25% | 1.77 | 1.77 | 1.83 | | 1.72 | 1.57 | 1.58 | 1.49 | 1.47 |
| Top 10% | 1.49 | 1.58 | 1.47 | | 1.44 | 1.51 | 1.60 | 1.57 | 1.44 |
| Top 5% | 1.60 | 1.70 | 1.64 | | 1.47 | 1.70 | 1.88 | 1.82 | 1.91 |
| Top 1% | 1.52 | 1.78 | 1.46 | | 1.80 | 1.02 | 0.87 | 0.99 | 0.95 |
| Top 0.1% | 1.11 | 1.33 | 1.07 | | 1.37 | -- | -- | -- | -- |
|  |  |  |  | |  |  |  |  |  |
| Gini among top incomes | |  |  | |  |  |  |  |  |
| Top 25% | 36.02 | 36.31 | 36.50 | | 34.83 | 30.37 | 30.51 | 29.89 | 28.66 |
| Top 10% | 29.92 | 31.30 | 29.49 | | 28.62 | 27.34 | 27.92 | 26.74 | 25.19 |
| Top 5% | 27.77 | 29.31 | 27.40 | | 25.86 | 27.21 | 28.22 | 27.13 | 26.46 |
| Top 1% | 24.47 | 27.47 | 23.02 | | 24.45 | 19.09 | 20.78 | 21.02 | 18.04 |
| Top 0.1% | 25.77 | 28.69 | 24.84 | | 28.67 | -- | -- | -- | -- |
|  |  |  |  | |  |  |  |  |  |
| Log pseudo-likelihood (LL/10^6^) | | | |  |  |  |  |  |  |
| Top 25% | -348.00 | -338.00 | -349.00 | | -346.00 | -384.00 | -375.00 | -385.00 | -382.00 |
| Top 10% | -144.00 | -140.00 | -145.00 | | -143.00 | -157.00 | -154.00 | -157.00 | -156.00 |
| Top 5% | -72.90 | -71.40 | -73.30 | | -72.70 | -79.80 | -77.80 | -80.20 | -79.40 |
| Top 1% | -15.60 | -15.00 | -15.30 | | -14.80 | -16.60 | -16.40 | -17.20 | -16.70 |
| Top 0.1% | -1.675 | -1.461 | -1.677 | | -1.751 | -- | -- | -- | -- |
|  |  |  |  | |  |  |  |  |  |

^i^ Too few observations to fit.

Source: Own analysis of ENIGH 2012, CEQ database.

Figure A2-1. Mean observed market income among respondents and nonresponse rate, by state


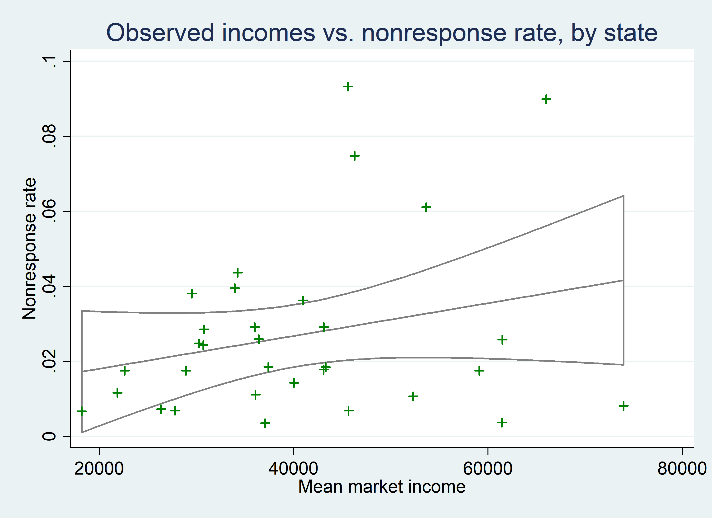


Notes: 95% confidence interval around linear fitted line is shown.

Source: Own analysis of ENIGH 2012, CEQ database.

Figure A2-2. Density function of disposable income per capita, with reference lognormal density


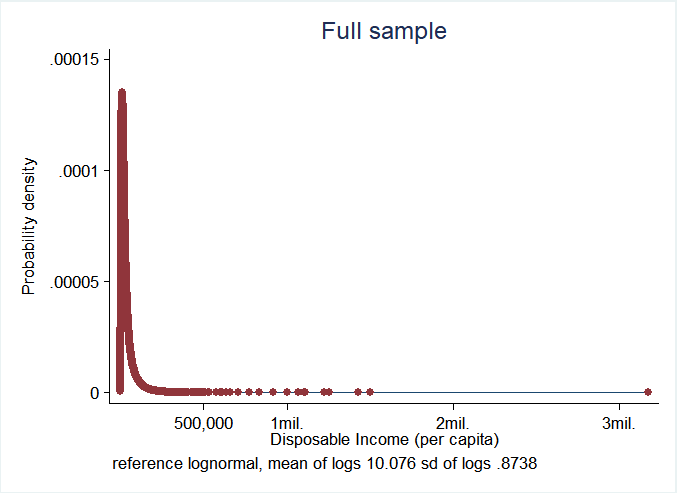

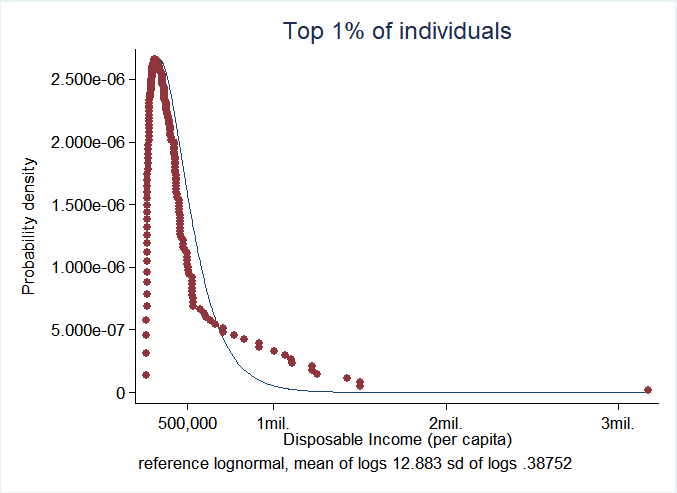


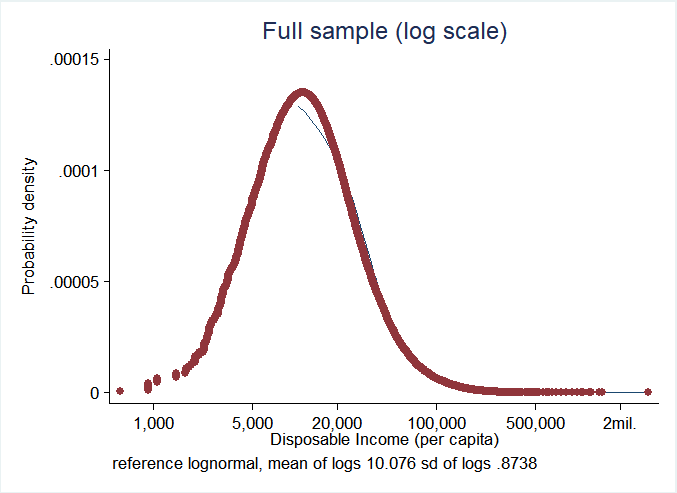

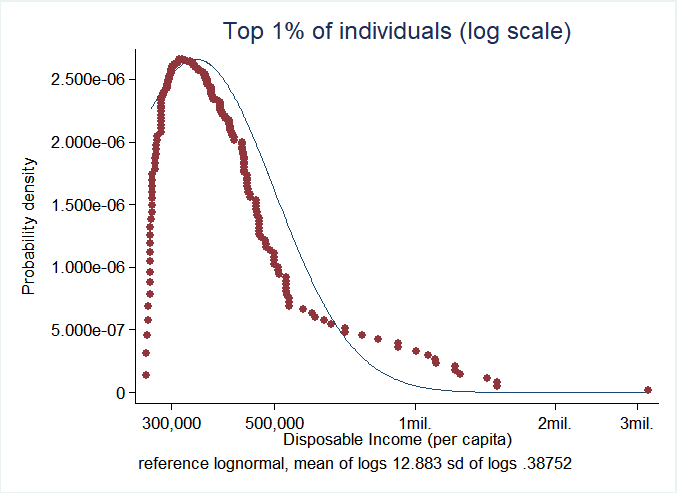


Source: Own analysis of ENIGH 2012, CEQ database.

Figure A2-3. Cumulative density function of disposable income per capita (log scale)


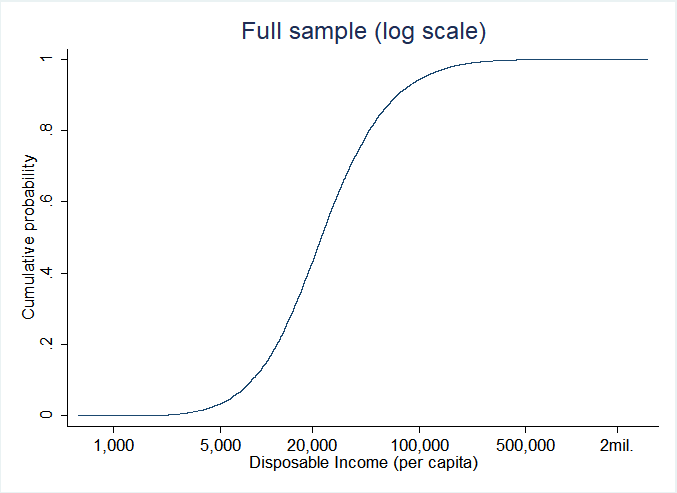

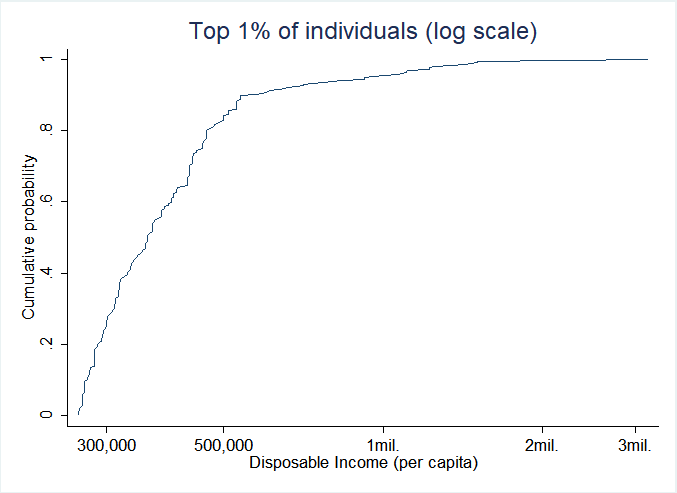


Source: Own analysis of ENIGH 2012, CEQ database.

Figure A2-4. Unit response probability by market income, logarithmic model of market income


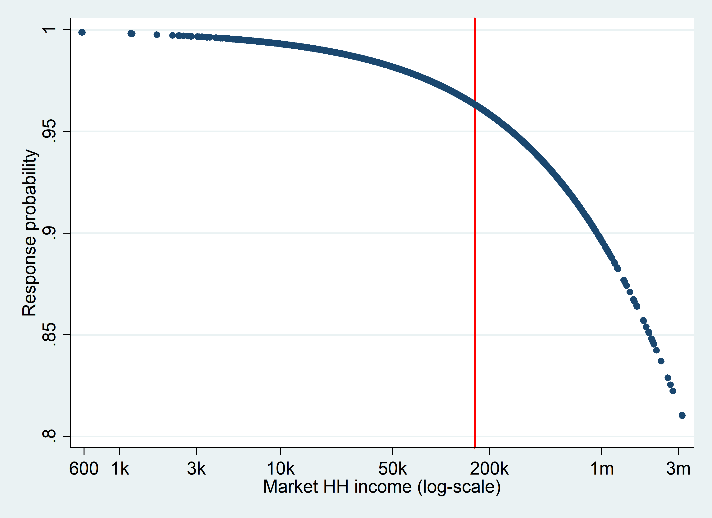

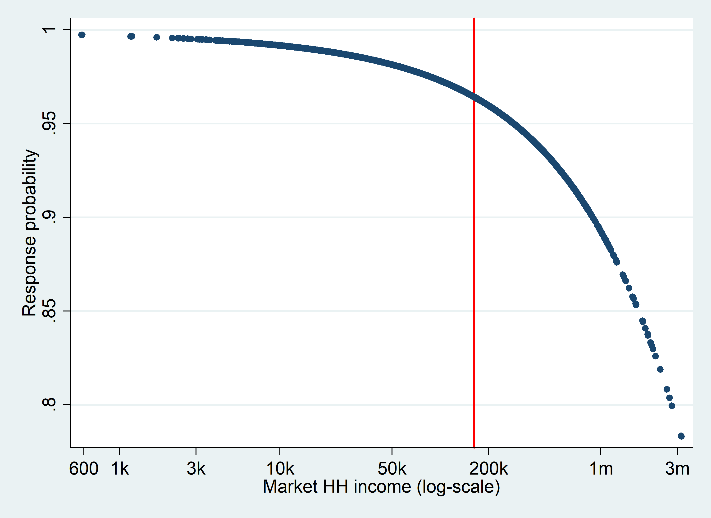


i. Logarithmic model of market income (model 1) ii. Quadratic logarithmic model of market income

θ_0_+θ_1_log(market inc.) θ_0_+θ_1_log(market inc.)^2^

Notes: Red line shows mean market household income in the corrected income distribution: i) 157,468 and ii) 157,665.

Source: Own analysis of ENIGH 2012, CEQ database.

Figure A2-5. Lorenz curve: income per capita, uncorrected versus unit-nonresponse corrected weights (model 1)


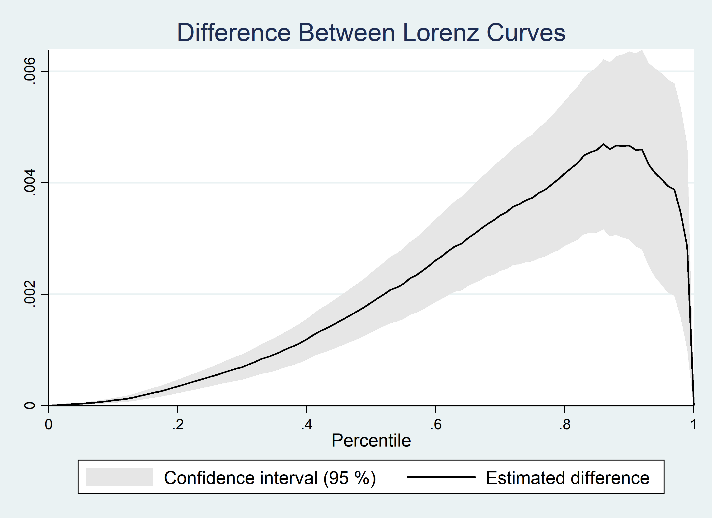

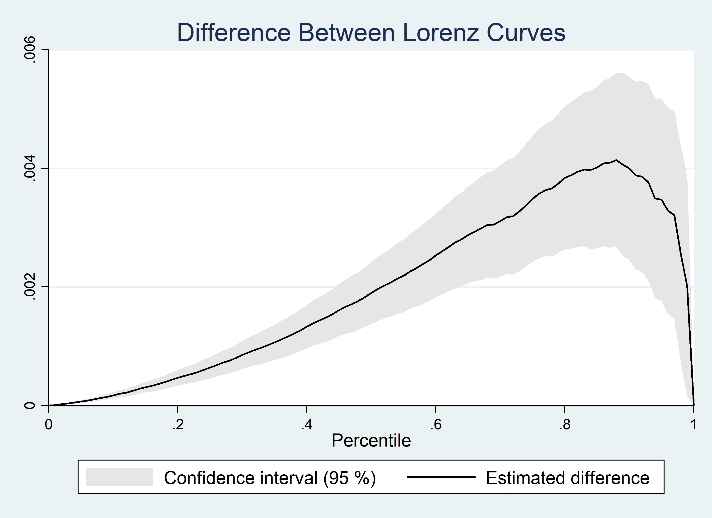


i. Market income per capita ii. Disposable income per capita

Notes: Positive values indicate that the Lorenz curve uncorrected for unit-nonresponse dominates, and shows less inequality than the corrected Lorenz curve. Distributions account for sampling weights and household size.

Source: Own analysis of ENIGH 2012, CEQ database.

Figure A2-6. Lorenz curve: market and disposable income per capita, top 10% of incomes replaced with Pareto I values


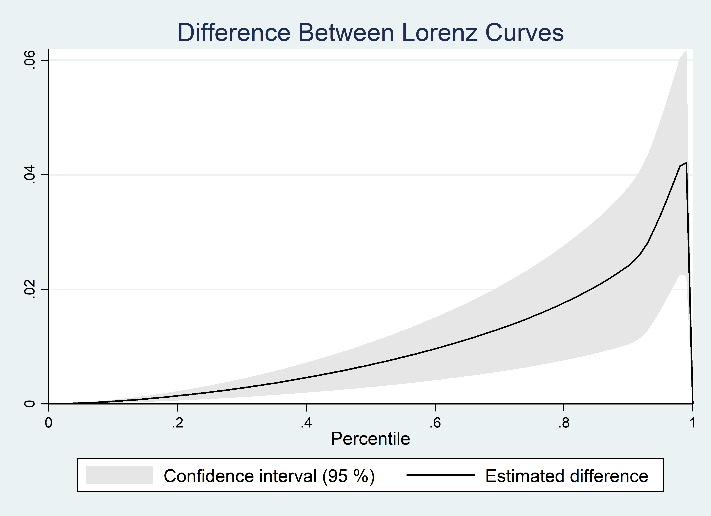

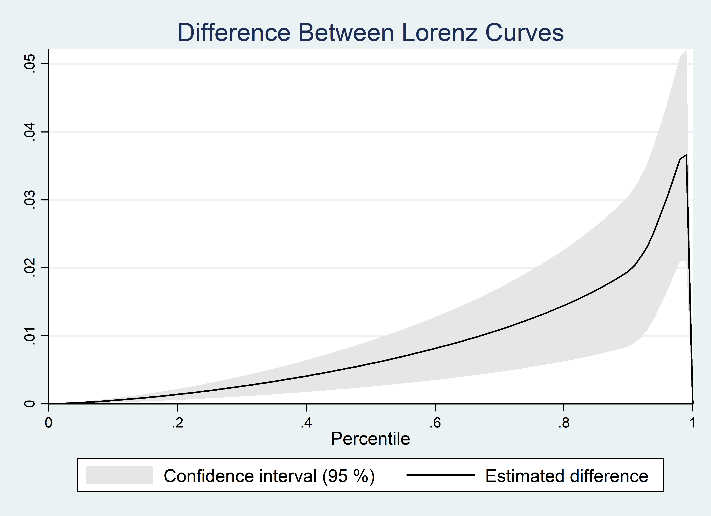


i. Market income per capita i. Disposable income per capita

Notes: Pareto replacing is performed on own income concept. Positive values indicate that the uncorrected Lorenz curve dominates, and shows less inequality than the corrected Lorenz curve. Distributions account for sampling weights and household size.

Source: Own analysis of ENIGH 2012, CEQ database.

Figure A2-7. Lorenz curve: market and disposable income per capita, top 10% of net market incomes replaced with Pareto I values, other income concepts imputed using CEQ Method


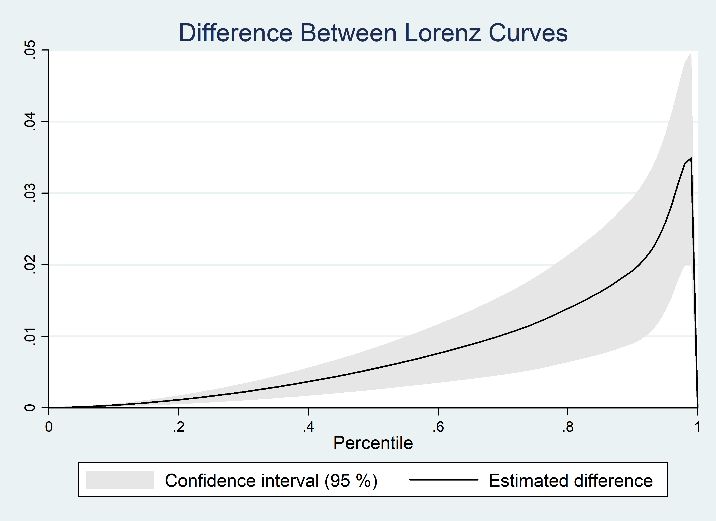

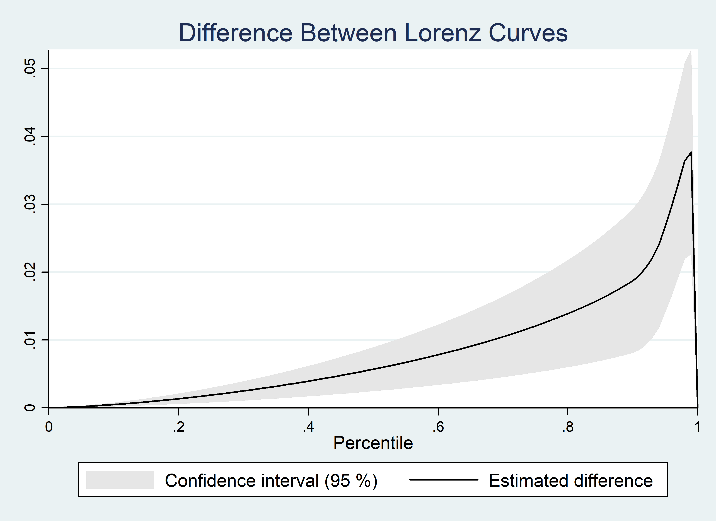


i. Market income per capita i. Disposable income per capita

Notes: Positive values indicate that the uncorrected Lorenz curve dominates, and shows less inequality than the corrected Lorenz curve. Distributions account for sampling weights and household size.

Source: Own analysis of ENIGH 2012, CEQ database.

Figure A2-8. Lorenz curve: market vs. disposable income per capita, uncorrected versus corrected income distributions


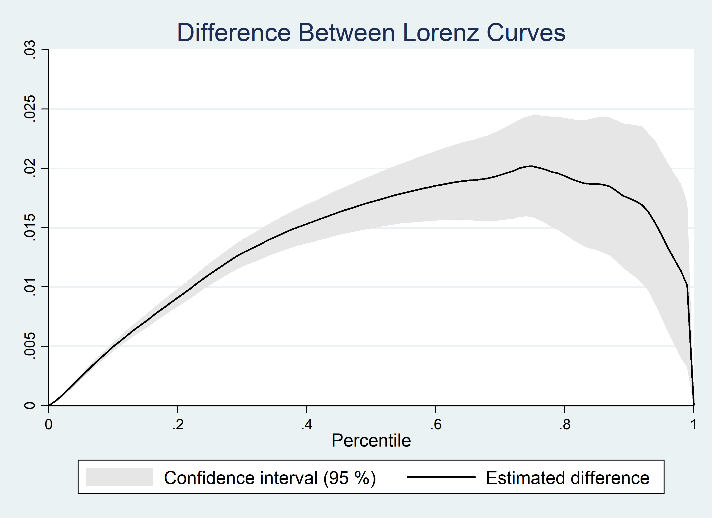

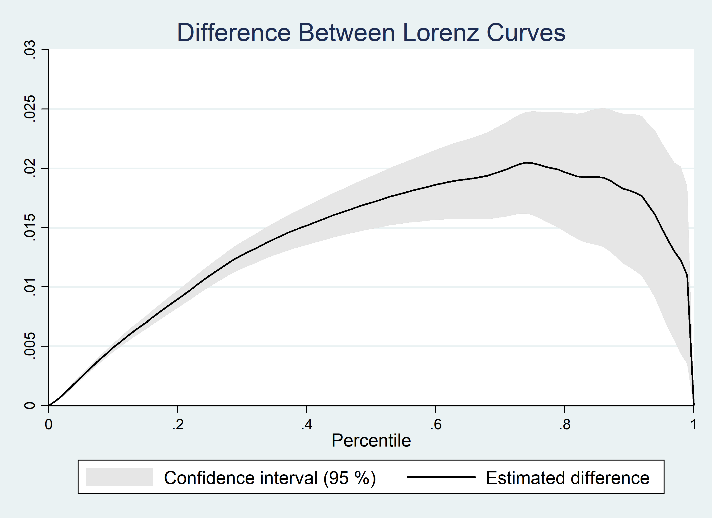


i. ENIGH sampling weights uncorrected for nonresponse ii. Weights corrected for nonresponse (mdel 1)


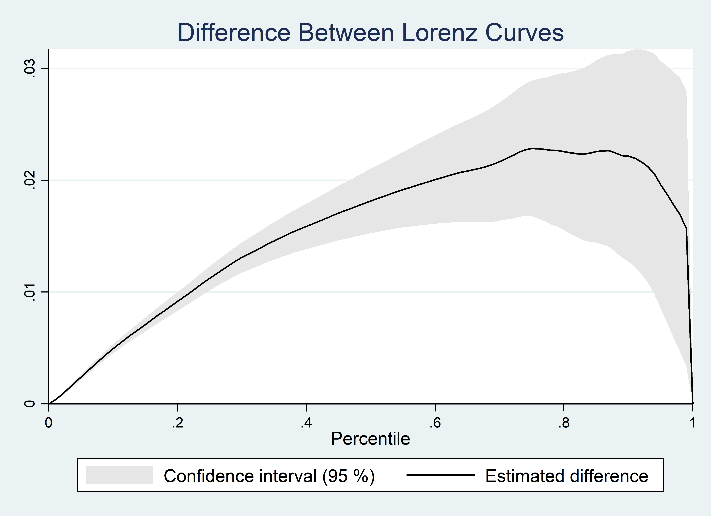

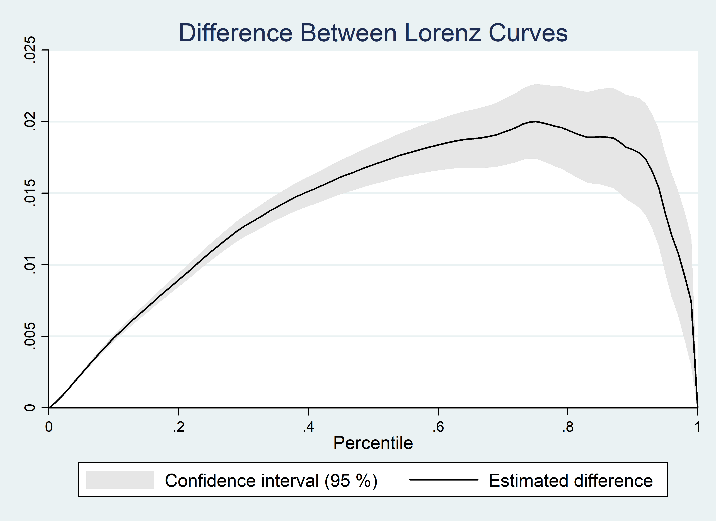


iii. Top 10% of incomes of each income concept iv. Top 10% of net market incomes replaced with

replaced with Pareto (I) values Pareto (I) values, other income concepts imputed by CEQ Method

Notes: Positive values indicate that disposable income Lorenz curve dominates, and shows less inequality than market income Lorenz curve. Distributions account for sampling weights and household size.

Source: Own analysis of ENIGH 2012, CEQ database.

Figure A2-9. Comparison of Pareto I and Pareto II models, various income concepts and top income cutoffs


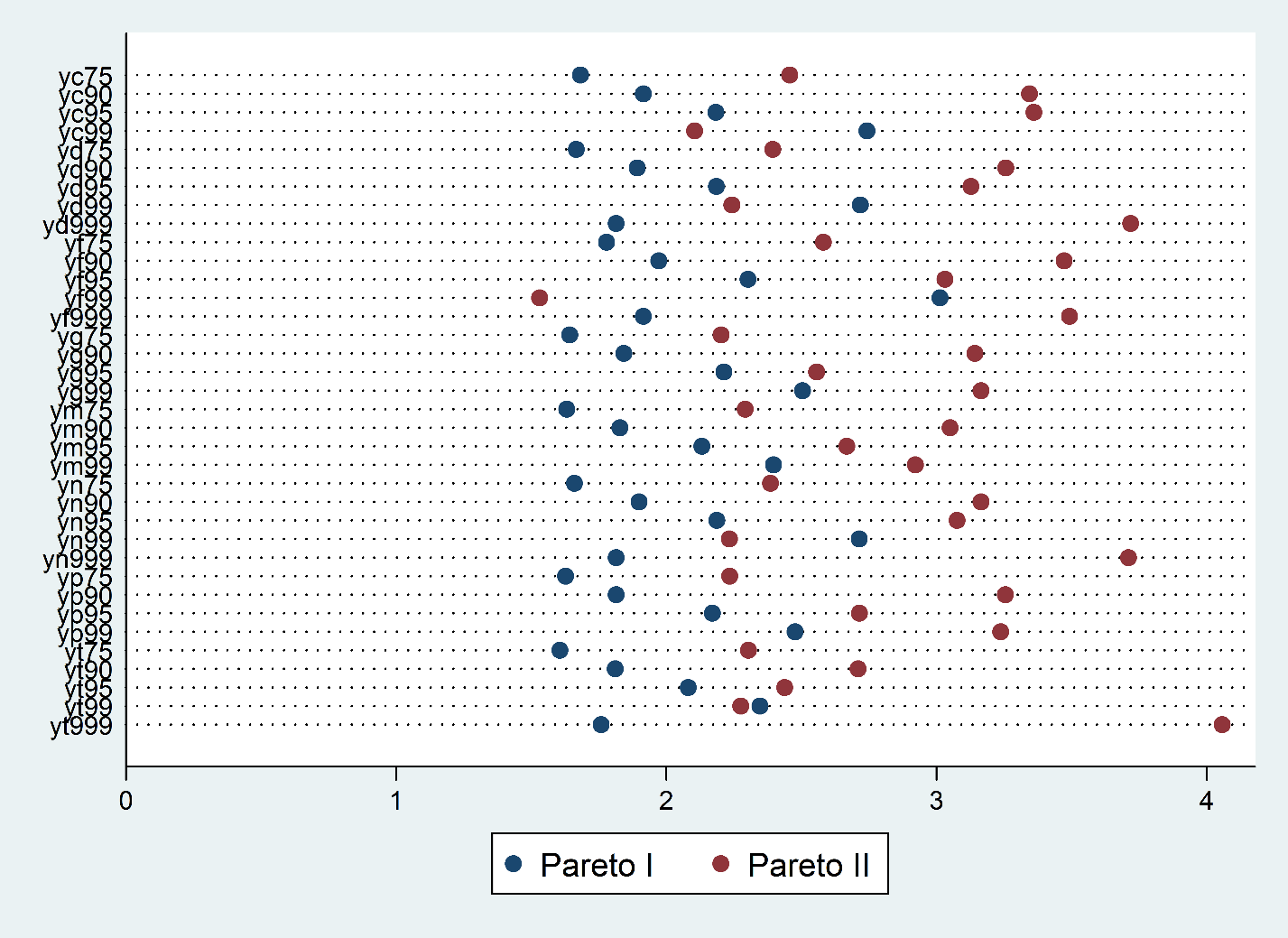


i. Pareto coefficient


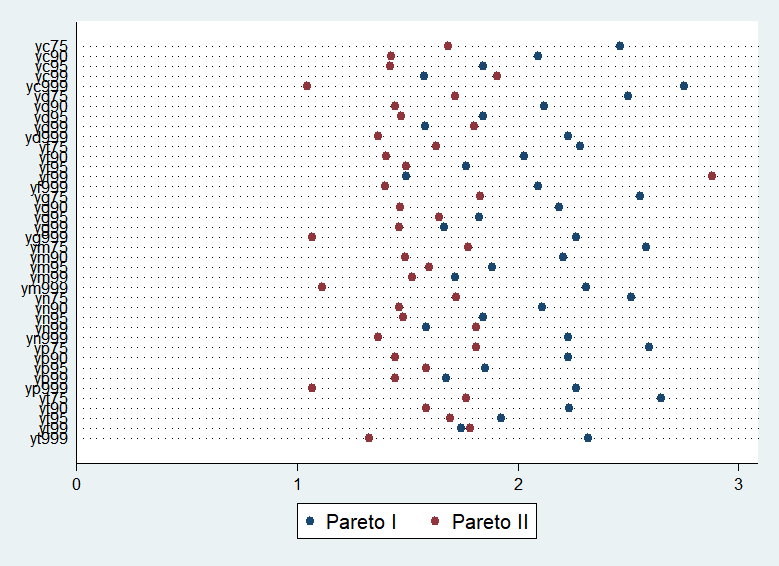


ii. Inverted Pareto coefficient


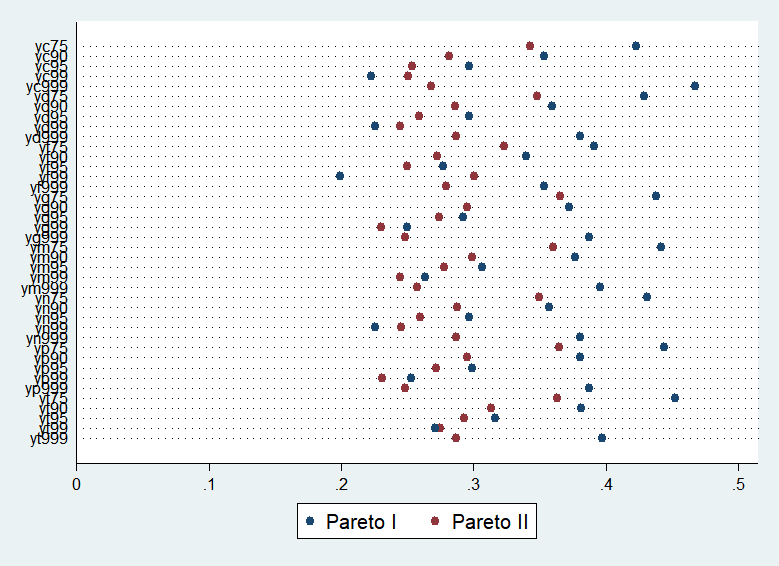


iii. Gini coefficient

Notes: ‘yp’ is market income plus pensions, ‘ym’ market income, ‘yt’ taxable income, ‘yg’ gross income, ‘yn’ net market income, ‘yd’ disposable income, ‘yc’ consumable income, ‘yf’ final income.

Source: Own analysis of ENIGH 2012, CEQ database.

**Appendix 3: Summary Statistics and Estimation Results for ENIGH 2010**

Table A3-1. Income summary statistics

|  |  |  | Corrected for nonresponse by reweighting | |
| --- | --- | --- | --- | --- |
|  | Disposable income  net of private & social security transfers per cap. | Disposable income  per cap. | Disposable income net of private & social security transfers per cap. | Disposable income per cap. |
| 99.9^th^ %ile | 545,865 | 589,642 | 453,354 | 483,936 |
| 99^th^ %ile | 189,013 | 213,799 | 173,058 | 193,413 |
| 95^th^ %ile | 88,022 | 101,324 | 81,463 | 93,939 |
| 90^th^ %ile | 61,335 | 70,977 | 57,014 | 65,694 |
| 75^th^ %ile | 36,295 | 41,974 | 33,983 | 39,407 |
| Mean | 30,060 | 36,050 | 27,873 | 33,633 |
| Median | 20,248 | 24,867 | 18,942 | 23,447 |
| 25^th^ %ile | 10,109 | 14,439 | 9,055 | 13,320 |
| 10^th^ %ile | 2,080 | 7,545 | 1,613 | 6,852 |
| 5^th^ %ile | 0 | 4,266 | 0 | 3,915 |
| 1^st^ %ile | -187 | 907 | -1 | 1,464 |
| Std. dev. | 45,109 | 48,409 | 40,476 | 43,516 |
| Skewness | 10.26 | 9.40 | 10.73 | 9.79 |
| Kurtosis | 268.15 | 223.42 | 296.90 | 245.85 |
| Sample | 27,593 | 27,593 | 27,385 | 27,385 |
| Top 0.1% inc. share | 2.70 | 2.35 | 2.60 | 2.27 |
| 0.1-1% inc. share | 8.09 | 7.45 | 7.82 | 7.17 |
| 1-5% inc. share | 15.93 | 15.13 | 15.72 | 14.87 |
| 5-10% inc. share | 12.11 | 11.60 | 12.08 | 11.50 |
| Gini (weighted data) | 49.73 (0.39) | 47.11 (0.36) | 49.88 (0.36) | 47.14 (0.33) |
| Gini (HH-size wted, no sampling wghts) | 51.21 (0.31) | 48.32 (0.29) | 51.49 (0.28) | 48.48 (0.26) |
| Gini (equal HH weights = unweighted) | 54.10 (0.41) | 50.45 (0.37) | 54.16 (0.35) | 50.36 (0.31) |
| Mean log dev. (GE0) | 0.555 (0.010) | 0.414 (0.007) | 0.578 (0.010) | 0.424 (0.006) |
| Theil index (GE1) | 0.487 (0.012) | 0.434 (0.010) | 0.484 (0.010) | 0.430 (0.009) |
| Half coef. of var.^2^ (GE2) | 1.126 (0.070) | 0.902 (0.052) | 1.054 (0.060) | 0.837 (0.044) |

Notes: MXN0 incomes, 62 household observations, are omitted in computations of the Gini.

Columns 1–2 relies on a sample weighted using ENIGH sampling weights (uncorrected). Columns 3–4 statistics are based on non-response correction weights estimated in the logarithmic model of disposable household income (model 1 in table A3-3). Column 2 statistics exclude 62 household observations with disposable income of 0. The statistics remain comparable to those in column 1, which are extremely robust to this exclusion (changing by 0.01 at the most.) Ginis and standard errors are multiplied by 100 for clarity of presentation. Standard errors on Gini coefficients are jackknife estimates.

Source: Own analysis of ENIGH 2010, LIS database.

Table A3-2. ENIGH 2010: Sample sizes, non-response rates, and mean incomes by state

| State code | State | Represented population | Fully interviewed households (individuals) | Type A non-responding HHs (%) | Mean dispos. HH income  per cap. |
| --- | --- | --- | --- | --- | --- |
| 01 | Aguascalientes | 1,192,473 | 329 (1,375) | 10 (2.9%) | 37,332 |
| 02 | Baja California | 3,174,663 | 504 (1,877) | 3 (0.6%) | 52,487 |
| 03 | Baja California Sur | 644,489 | 299 (1,093) | 4 (1.3%) | 46,743 |
| 04 | Campeche | 824,907 | 427 (1,620) | 12 (2.7%) | 37,142 |
| 05 | Coahuila de Zarag. | 2,759,794 | 564 (2,137) | 22 (3.8%) | 37,167 |
| 06 | Colima | 653,275 | 361 (1,267) | 23 (6.0%) | 45,933 |
| 07 | Chiapas | 4,820,282 | 2,805 (11,814) | 71 (2.5%) | 17,185 |
| 08 | Chihuahua | 3,414,766 | 836 (2,906) | 15 (1.8%) | 36,741 |
| 09 | Distrito Federal | 8,806,329 | 2,799 (9,681) | 55 (1.9%) | 61,666 |
| 10 | Durango | 1,637,472 | 497 (1,950) | 47 (8.6%) | 28,842 |
| 11 | Guanajuato | 5,507,127 | 1,901 (7,810) | 38 (2.0%) | 30,833 |
| 12 | Guerrero | 3,394,244 | 868 (3,677) | 18 (2.0%) | 23,401 |
| 13 | Hidalgo | 2,676,807 | 513 (2,191) | 12 (2.3%) | 23,833 |
| 14 | Jalisco | 7,378,707 | 595 (2,309) | 31 (5.0%) | 42,208 |
| 15 | México | 15,227,945 | 2,748 (11,244) | 110 (3.8%) | 36,887 |
| 16 | Mich. de Ocampo | 4,355,975 | 700 (2,643) | 20 (2.8%) | 27,148 |
| 17 | Morelos | 1,781,315 | 462 (1,744) | 17 (3.5%) | 35,519 |
| 18 | Nayarit | 1,089,518 | 408 (1,592) | 2 (0.5%) | 35,718 |
| 19 | Nuevo León | 4,659,638 | 411 (1,512) | 21 (4.9%) | 49,976 |
| 20 | Oaxaca | 3,808,423 | 1,051 (4,315) | 19 (1.8%) | 25,468 |
| 21 | Puebla | 5,790,569 | 662 (2,793) | 41 (5.8%) | 28,319 |
| 22 | Querétaro | 1,841,607 | 452 (1,771) | 17 (3.6%) | 41,294 |
| 23 | Quintana Roo | 1,341,524 | 350 (1,242) | 4 (1.1%) | 48,446 |
| 24 | San Luis Potosí | 2,588,544 | 539 (2,240) | 16 (2.9%) | 31,307 |
| 25 | Sinaloa | 2,772,382 | 458 (1,695) | 5 (1.1%) | 34,806 |
| 26 | Sonora | 2,669,362 | 649 (2,450) | 7 (1.1%) | 38,146 |
| 27 | Tabasco | 2,243,345 | 487 (1,876) | 6 (1.2%) | 28,160 |
| 28 | Tamaulipas | 3,283,331 | 558 (1,946) | 40 (6.7%) | 36,636 |
| 29 | Tlaxcala | 1,175,903 | 380 (1,551) | 3 (0.8%) | 28,086 |
| 30 | Veracruz de Ign. | 7,635,224 | 898 (3,285) | 35 (3.8%) | 28,373 |
| 31 | Yucatán | 1,956,933 | 2,719 (10,449) | 166 (5.8%) | 32,652 |
| 32 | Zacatecas | 1,493,710 | 425 (1,582) | 2 (0.5%) | 25,617 |
|  | Nationwide | 112,572,638 | 27,655 (107,637) | 892 (3.1%) | 36,050 |

Note: Mean incomes account for sampling weights but are computed only among responding households, and may not be representative of underlying population.

Source: Instituto Nacional de Estadística y Geografía (INEGI, 2011), Encuesta Nacional de Ingresos y Gastos de los Hogares 2010: Diseño muestral; Own analysis of ENIGH 2010, LIS database.

Table A3-3. Estimation results for various univariate logistic models of response probability

|  |  |  |  |  |  | Gini (s.e.): Dispos. income per capita | | |
| --- | --- | --- | --- | --- | --- | --- | --- | --- |
| Specification of $g\left( x_{i},\theta\right)$ | $\hat{\theta_{0}}$ (s.e.) | $\hat{\theta_{1}}$ (s.e.) | Sum of Squared Weighted Errors | Factor of Proportio-nality (σ^2^) | AIC  SIC | Weighted data | HH-size wted, no sampling wghts | Equal HH weights = unweighted |
| Uncorrected |  |  |  |  |  | 47.11  (0.36) | 48.32 (0.29) | 50.45  (0.37) |
| 1: θ_0_+θ_1_log(dispos. inc.) | -2.926 (0.544) | 0.162 (0.049) | 493.38 | 16.763 | 91.54  88.92 | 47.14 (0.33) | 48.48 (0.26) | 50.36 (0.31) |
| 2: θ_0_+θ_1_log(dispos. inc.  net of transfers) | -2.352 (0.414) | 0.105 (0.039) | 372.22 | 16.033 | 82.52  79.91 | 46.65 (0.35) | 47.92 (0.28) | 49.96 (0.35) |
| 3: θ_0_+θ_1_log(disp. inc.pc) | -2.928 (0.342) | 0.182 (0.034) | 374.71 | 11.160 | 82.73  80.12 | 46.98 (0.33) | 48.39 (0.25) | 50.18 (0.30) |
| 4: θ_0_+θ_1_log(dispos. inc.)^2^ | -2.053 (0.284) | 0.008 (0.002) | 491.37 | 16.524 | 91.41  88.79 | 46.96 (0.32) | 48.29 (0.25) | 50.15 (0.31) |
| 5: θ_0_+θ_1_log(disp. inc.pc)^2^ | -2.069 (0.178) | 0.010 (0.002) | 368.43 | 10.678 | 82.19  79.58 | 46.63 (0.32) | 48.02 (0.25) | 49.78 (0.28) |
| 6: θ_0_+θ_1_10^-6^ dispos. inc. | -1.229 (0.052) | 1.223 (0.475) | 503.94 | 16.835 | 92.22  89.60 | 45.08 (0.28) | 46.39 (0.21) | 48.29 (0.24) |
| 7: θ_0_+θ_1_10^-6^ disp. inc.pc | -1.301 (0.040) | 6.348 (1.315) | 354.40 | 9.161 | 80.95  78.34 | 43.56 (0.26) | 44.93 (0.20) | 46.15 (0.21) |
| 8: θ_0_+θ_1_10^-12^dispos. inc.^2^ | -1.229 (0.048) | 6.730 (2.902) | 458.64 | 12.692 | 89.20  86.59 | 43.00 (0.25) | 44.42 (0.19) | 46.33 (0.22) |
| 9: θ_0_+θ_1_(10^-3^dispos. inc.)^½^ | -1.479 (0.115) | 0.039 (0.012) | 491.04 | 15.258 | 91.39  88.77 | 45.92 (0.29) | 47.24 (0.22) | 49.06 (0.26) |
| 10:θ_0_+θ_1_(10^-3^disp. inc.pc)^½^ | -1.543 (0.080) | 0.084 (0.016) | 357.27 | 9.093 | 81.21  78.59 | 45.03 (0.29) | 46.40 (0.22) | 47.88 (0.23) |

Note: Ginis and standard errors are multiplied by 100 for clarity of presentation. Standard errors on Gini coefficients are jackknife estimates.

Source: Own analysis of ENIGH 2010, LIS database.

Table A3-4. Estimation results for selected multivariate models of response probability

|  |  |  |  |  |  |  |  |  | Gini (s.e.): Dispos. inc. pc. | | |
| --- | --- | --- | --- | --- | --- | --- | --- | --- | --- | --- | --- |
| Specification of *g(x)* | $\hat{\theta_{0}}$ (s.e.) | $\hat{\theta_{1}}$ (s.e.) | $\hat{\theta_{2}}$ (s.e.) | $\hat{\theta_{3}}$ (s.e.) | $\hat{\theta_{4}}$ (s.e.) | Sum of squared wghted. errors | Factor of propor-tionality (σ^2^) | AIC  SIC | Weighted data | HH-size wted, no sampling wghts | Equal HH weights=  unwghtd |
| Uncorrected |  |  |  |  |  |  |  |  | 47.11  (0.36) | 48.32 (0.29) | 50.45  (0.37) |
| θ_0_+θ_1_10^-6^dispos. inc.  +θ_2_10^-12^dispos. inc.^2^ | -1.241 (0.149) | 0.304 (3.802) | 5.602 (14.77) |  |  | 458.51 | 12.768 | 91.19  88.49 | 43.17 (0.24) | 44.58 (0.20) | 46.48 (0.22) |
| θ_0_+θ_1_log(dispos. inc.pc) +θ_2_urban/10+θ_3_age+θ_4_age^2^ | -3.343 (1.010) | 0.206 (0.059) | -0.062 (0.151) | -1.596 (6.598) | 5.735 (10.102) | 306.20 | 7.117 | 82.27  80.32 | 46.90 (0.33) | 48.36 (0.25) | 50.20 (0.29) |
| θ_0_+θ_1_log(dispos. inc.) +θ_2_urban/10+θ_3_age+θ_4_age^2^ | -2.959 (1.063) | 0.103 (0.068) | 0.181 (0.146) | -0.261 (6.455) | 4.997 (10.239) | 386.77 | 8.079 | 89.75  87.79 | 47.27 (0.35) | 48.64 (0.27) | 50.66 (0.32) |
| θ_0_+θ_1_log(dispos. inc.pc) +θ_2_sch.yrs+θ_3_hsize+θ_4_hsize^2^ | 0.474 (0.148) | -0.002 (0.013) | -0.001 (0.004) | -57.058 (4.605) | 290.871 (42.930) | 8.53 | 0.334 | -32.32  -34.28 | 45.56 (0.39) | 47.09 (0.31) | 49.01 (0.30) |
| θ_0_+θ_1_log(dispos. inc.pc) + θ_2_sch.yrs+θ_3_hsize+θ_4_male | 0.106 (0.295) | -0.039 (0.028) | 0.012 (0.008) | -21.470 (1.157) | -0.203 (0.137) | 49.61 | 1.440 | 24.03  22.08 | 46.15 (0.47) | 48.00 (0.37) | 50.04 (0.33) |
| θ_0_+θ_1_log(dispos. inc.pc) +θ_2_postsec.attend+θ_3_male | -2.006 (0.461) | 0.111 (0.038) | 0.996 (0.702) | -0.389 (0.320) |  | 318.19 | 7.664 | 81.50  79.05 | 45.16 (0.32) | 46.71 (0.25) | 48.58 (0.29) |
| θ_0_+θ_1_log(dispos. inc.pc) +θ_2_postsec.attend+θ_3_empl. | -0.912 (0.957) | 0.068 (0.043) | 1.403 (0.925) | -1.159 (0.783) |  | 288.16 | 8.507 | 78.33  75.87 | 44.29 (0.31) | 45.91 (0.25) | 47.92 (0.33) |

Note: Standard errors on Gini coefficients are jackknife estimates. Variables are normalized: age=(years-12)/100; HH size=(#-1)/100. Measures of fit are not entirely comparable across models with different controls, because of different sample sizes.

Source: Own analysis of ENIGH 2010, LIS database.

Table A3-5. Replacement of top incomes with Pareto I distribution: uncorrected vs. corrected Ginis

| Cutoff percentile | Disposable income net of transfers per capita | Disposable income per capita |
| --- | --- | --- |
| Nonparametric Gini among bottom incomes | | |
| Top 25% | 33.17 (0.27) | 29.70 (0.22) |
| Top 10% | 37.32(0.24) | 34.56 (0.21) |
| Top 5% | 40.34 (0.24) | 37.80 (0.22) |
| Top 1% | 45.32 (0.28) | 42.92 (0.26) |
| Top 0.1% | 48.52 (0.34) | 46.01 (0.31) |
|  |  |  |
| Nonparametric Gini among top incomes | | |
| Top 25% | 32.04 (0.63) | 31.20 (0.58) |
| Top 10% | 29.53 (0.85) | 28.42 (0.78) |
| Top 5% | 28.36 (1.06) | 27.00 (0.97) |
| Top 1% | 25.85 (1.53) | 24.26 (1.44) |
| Top 0.1% | 16.34 (2.51) | 15.34 (2.27) |
|  |  |  |
| Pareto (type I) coefficient | | |
| Top 25% | 1.824 (0.027) | 1.851 (0.027) |
| Top 10% | 2.017 (0.044) | 2.072 (0.044) |
| Top 5% | 2.146 (0.067) | 2.221 (0.066) |
| Top 1% | 2.330 (0.150) | 2.476 (0.156) |
| Top 0.1% | 2.870 (0.347) | 3.249 (0.427) |
|  |  |  |
| Parametric Gini among top incomes | | |
| Top 25% | 37.77 | 37.02 |
| Top 10% | 32.97 | 21.80 |
| Top 5% | 30.38 | 29.06 |
| Top 1% | 27.32 | 25.31 |
| Top 0.1% | 21.10 | 18.19 |
|  |  |  |
| Semiparametric Gini | | |
| Top 25% | **53.75** | **49.97** |
| Top 10% | **51.37** | **48.13** |
| Top 5% | **50.53** | **47.57** |
| Top 1% | **49.96** | **47.23** |
| Top 0.1% | **49.80** | **47.14** |
|  |  |  |
| Uncorrected | 49.73 | 47.11 |
| Gini correction | | |
| Top 25% | **+4.02** | **+2.86** |
| Top 10% | **+1.64** | **+1.02** |
| Top 5% | **+0.80** | **+0.46** |
| Top 1% | **+0.23** | **+0.12** |
| Top 0.1% | **+0.07** | **+0.03** |
|  |  |  |

Source: Own analysis of ENIGH 2010, LIS database.

Table A3-6. Summary results of correction methods: corrected Ginis for all income concepts

|  | Disposable income net of transfers per capita | +  Cash-like private & social security transfers (after-tax) | Disposable income per cap. |
| --- | --- | --- | --- |
|  | Value **+%pt. correct.** | %pt. Δ | Value **+%pt. correct.** |
| ***Gini coefficient*** | |  |  |
| Uncorrected | 49.7 | –2.6 | 47.1 |
|  |  |  |  |
| Correction by reweighting (models in table A3-3) | | | |
| Minimum | 45.9 **–3.8** | –2.9 | 43.0 **–4.1** |
| Mean | 48.6 **–1.1** | –2.9 | 45.7 **–1.4** |
| Median | 49.1 **–0.6** | –2.8 | 46.3 **–0.8** |
| Maximum | 50.6 **+0.9** | –3.5 | 47.1 **+0.0** |
|  |  |  |  |
| Correction by Pareto (type I) replacing of own income concept | | | |
| Minimum | 49.8 **+0.1** | –2.7 | 47.1 **+0.0** |
| Mean | 51.1 **+1.4** | –3.1 | 48.0 **+0.9** |
| Median | 50.5 **+0.8** | –2.9 | 47.6 **+0.5** |
| Maximum | 53.8 **+4.1** | –3.8 | 50.0 **+2.9** |
|  |  |  |  |
| ***Top 10 percent income share*** | |  |  |
| Uncorrected | 38.8 | –2.3 | 36.5 |
|  |  |  |  |
| Correction by reweighting (models in table A3-3) | | | |
| Minimum | 34.5 **–4.3** | –2.3 | 32.2 **–4.3** |
| Mean | 37.0 **–1.8** | –2.3 | 34.7 **–1.8** |
| Median | 37.5 **–1.3** | –2.4 | 35.1 **–1.4** |
| Maximum | 38.2 **–0.6** | –2.4 | 35.8 **–0.7** |
|  |  |  |  |
| Correction by Pareto (type I) replacing of own income concept | | | |
| Minimum | 38.9 **+0.1** | –2.4 | 36.5 **+0.0** |
| Mean | 39.9 **+1.1** | –2.3 | 37.6 **+1.1** |
| Median | 39.3 **+0.5** | –2.3 | 37.0 **+0.5** |
| Maximum | 42.5 **+3.7** | –2.2 | 40.3 **+3.8** |
|  |  |  |  |
| ***Top 1 percent income share*** | |  |  |
| Uncorrected | 10.8 | –1.0 | 9.8 |
|  |  |  |  |
| Correction by reweighting (models in table A3-3) | | | |
| Minimum | 8.4 **–2.4** | –0.7 | 7.7 **–2.1** |
| Mean | 9.7 **–1.1** | –0.9 | 8.8 **–1.0** |
| Median | 9.9 **–0.9** | –0.9 | 9.0 **–0.8** |
| Maximum | 10.4 **–0.4** | –1.0 | 9.4 **–0.4** |
|  |  |  |  |
| Correction by Pareto (type I) replacing of own income concept | | | |
| Minimum | 10.9 **+0.1** | –1.1 | 9.8 **+0.0** |
| Mean | 11.9 **+1.1** | –1.1 | 10.8 **+1.0** |
| Median | 11.7 **+0.9** | –1.0 | 10.7 **+0.9** |
| Maximum | 13.5 **+2.7** | –1.0 | 12.5 **+2.7** |
|  |  |  |  |

Notes: Pc.pt. differences from uncorrected Ginis in **bold**. These Ginis and differences in them arise from ‘Gini (HH-size & sampling weighted data)’ in tables A3-1, and ‘Semiparametric Gini’ in table A3-5. Ginis and percentage point changes are multiplied by 100 for clarity of presentation.

Source: Own analysis of ENIGH 2010, LIS database.

Table A3-7. Generalized Pareto (type II) results, income per capita

| Cutoff percentile | Disposable income net of transfers per capita | Disposable income per capita |
| --- | --- | --- |
| Pareto (type II) shape coefficient ξ | |  |
| Top 25% | 0.403 (.020) | 0.390 (.019) |
| Top 10% | 0.401 (.030) | 0.388 (.029) |
| Top 5% | 0.430 (.043) | 0.403 (.040) |
| Top 1% | 0.439 (.082) | 0.414 (.074) |
| Top 0.1% | 0.048 (.101) | 0.060 (.096) |
|  |  |  |
| Pareto (type II) scale coefficient, log(σ) | |  |
| Top 25% | 10.039 (.029) | 10.175 (.029) |
| Top 10% | 10.417 (.043) | 10.534 (.042) |
| Top 5% | 10.658 (.063) | 10.777 (.058) |
| Top 1% | 11.294 (.134) | 11.359 (.123) |
| Top 0.1% | 12.449 (.193) | 12.415 (.196) |
|  |  |  |
| 1/ξ | |  |
| Top 25% | 2.481 | 2.566 |
| Top 10% | 2.495 | 2.580 |
| Top 5% | 2.327 | 2.481 |
| Top 1% | 2.278 | 2.415 |
| Top 0.1% | 20.867 | 16.637 |
|  |  |  |
| Inverted Pareto coefficient | |  |
| Top 25% | 1.675 | 1.638 |
| Top 10% | 1.669 | 1.633 |
| Top 5% | 1.753 | 1.675 |
| Top 1% | 1.782 | 1.707 |
| Top 0.1% | 1.050 | 1.064 |
|  |  |  |
| Gini among top incomes | |  |
| Top 25% | 32.17 | 31.42 |
| Top 10% | 29.78 | 28.76 |
| Top 5% | 29.21 | 27.67 |
| Top 1% | 27.61 | 25.63 |
| Top 0.1% | 16.86 | 15.87 |
|  |  |  |
| Log pseudo-likelihood (LL/10^6^) | |  |
| Top 25% | -321.00 | -325.00 |
| Top 10% | -133.00 | -134.00 |
| Top 5% | -67.90 | -68.40 |
| Top 1% | -14.30 | -14.30 |
| Top 0.1% | -1.497 | -1.488 |
|  |  |  |

Notes: individual sampling-weighted sample.

Source: Own analysis of ENIGH 2010, LIS database.

Figure A3-1. Mean observed market income among respondents, and nonresponse rate, by state


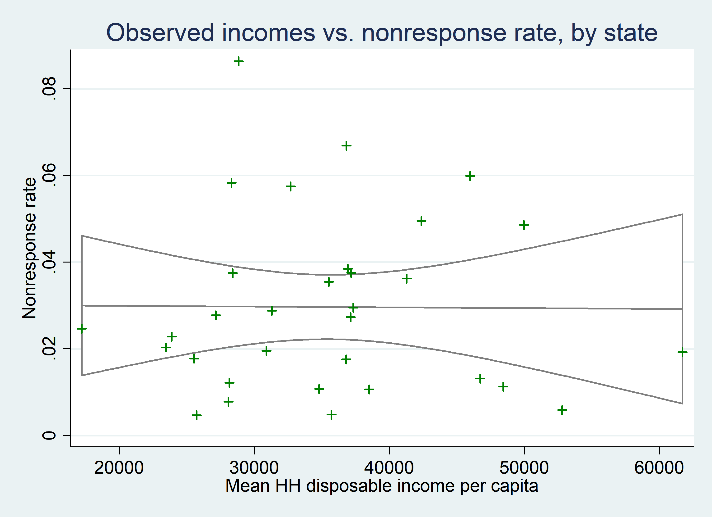


Notes: 95% confidence interval around linear fitted line is shown.

Source: Own analysis of ENIGH 2010, LIS database.

Figure A3-2. Density function of income per capita, with reference lognormal density


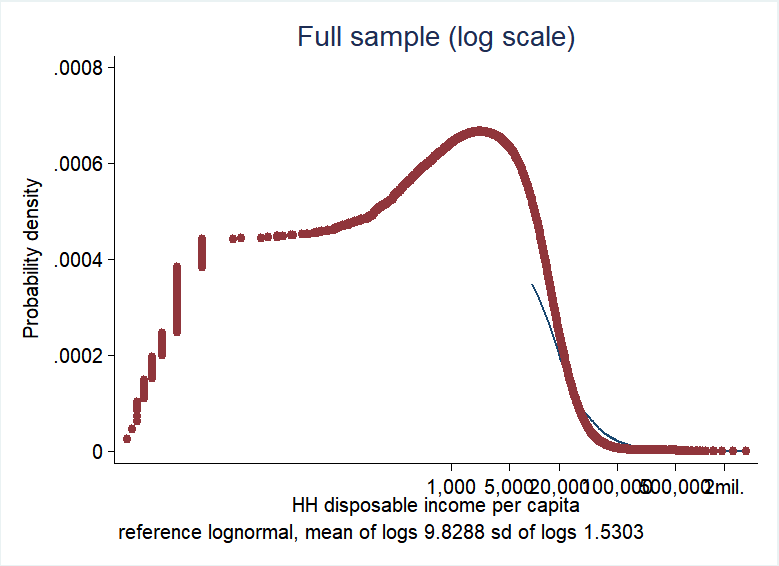

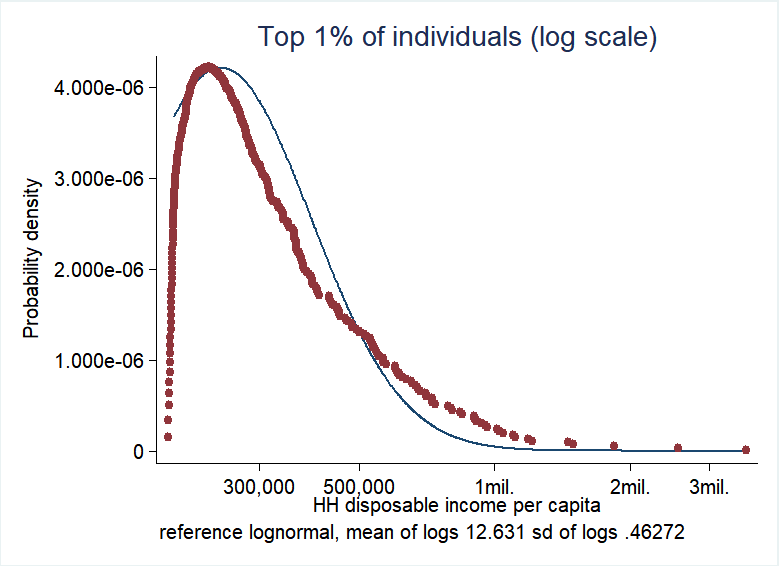


i. Disposable income net of transfers, full sample ii. Disposable income net of transfers, top 1%


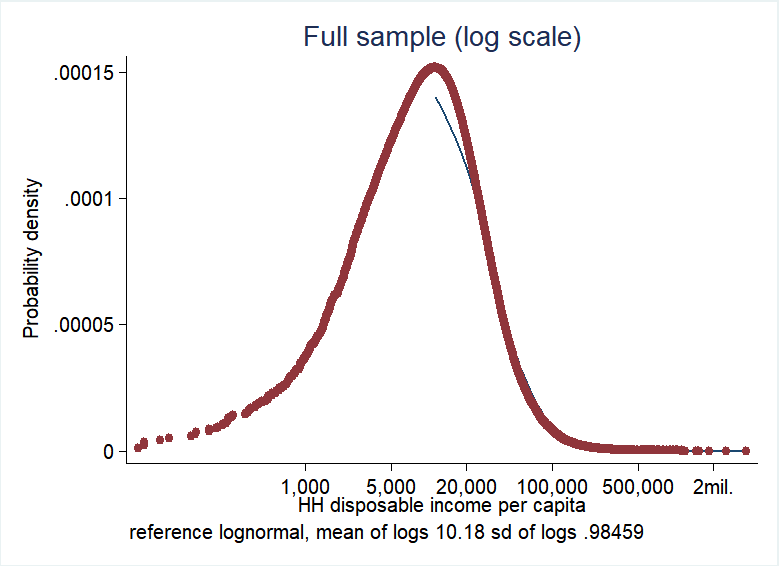

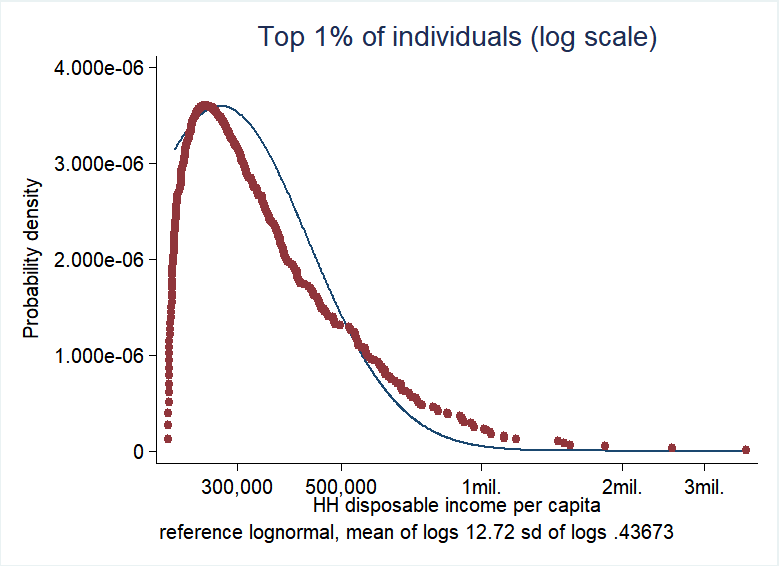


iii. Disposable income, full sample iv. Disposable income, top 1%

Notes: Non-positive incomes omitted.

Source: Own analysis of ENIGH 2010, LIS database.

Figure A3-3. Cumulative density function of disposable income per capita (log scale)


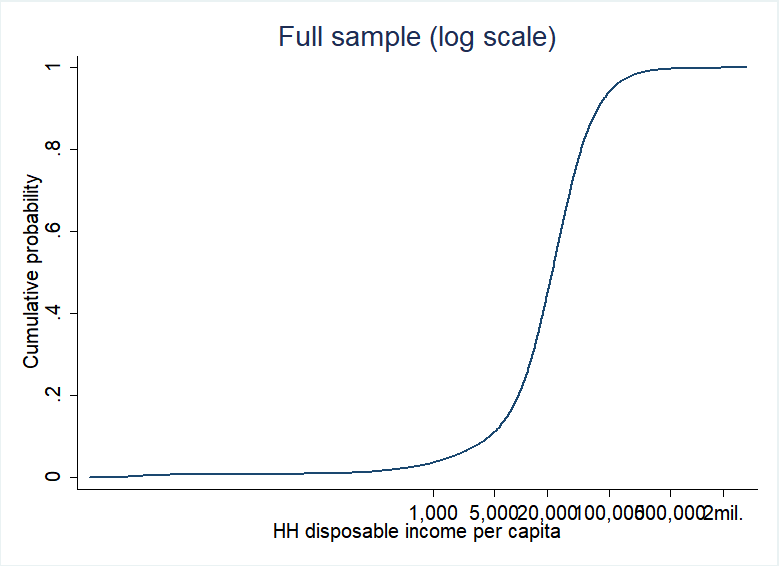

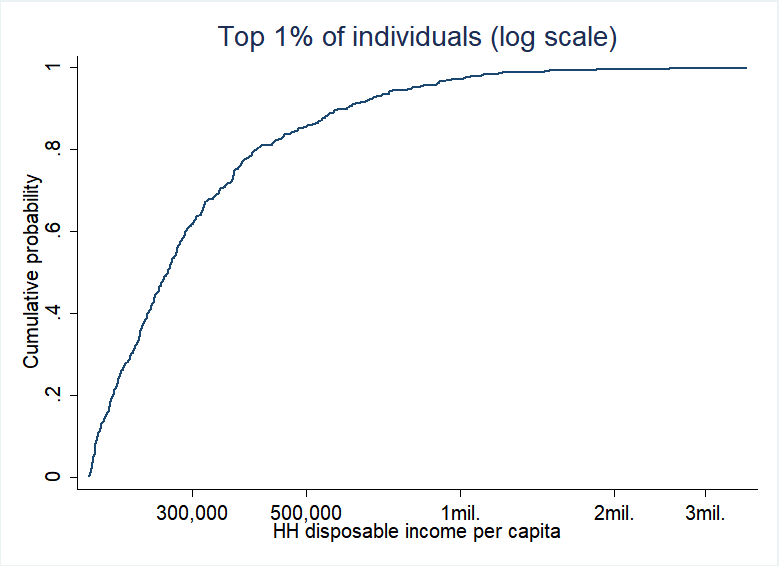


i. Disposable income net of transfers, full sample ii. Disposable income net of transfers, top 1%


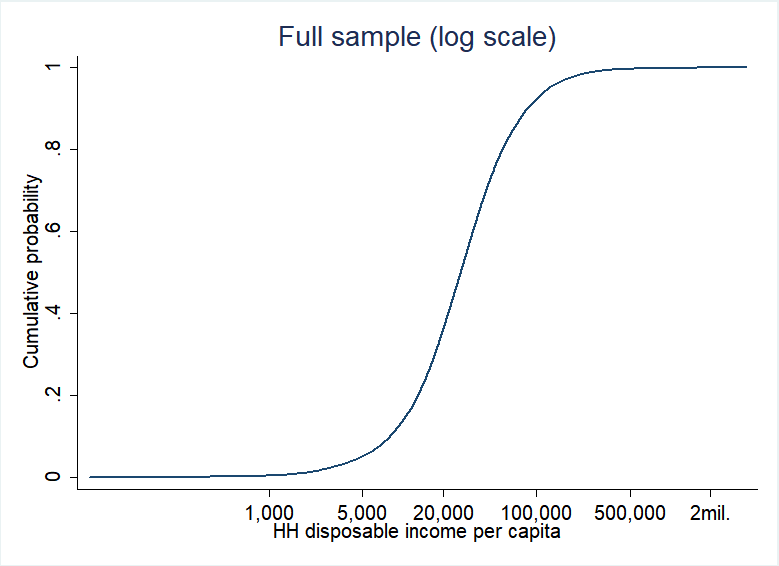

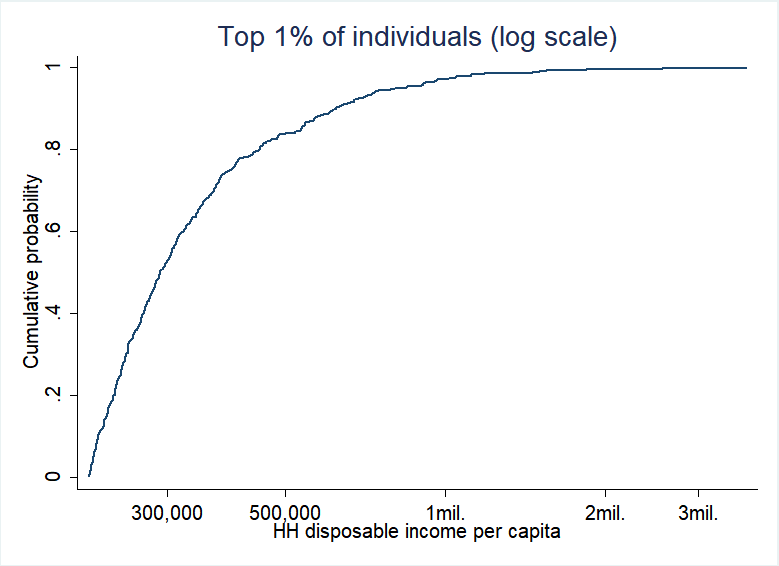


iii. Disposable income, full sample iv. Disposable income, top 1%

Source: Own analysis of ENIGH 2010, LIS database.

Figure A3-4. Unit response probability by disposable income, logarithmic model of disposable income (models 1 and 3)


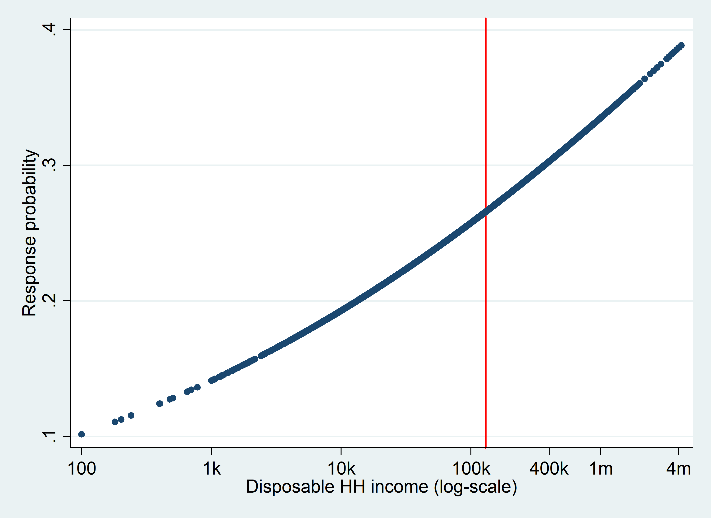

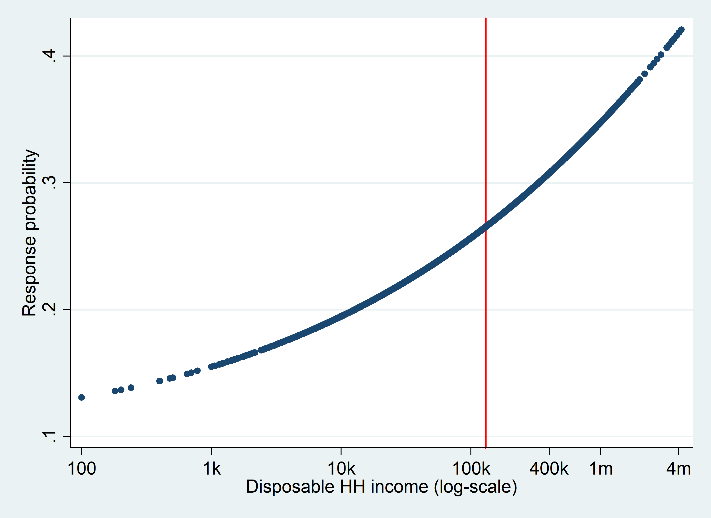


i. Logarithmic model of disposable income ii. Quadratic logarithmic model of disposable income

(model 1) θ_0_+θ_1_log(disposable inc.) (Model 3) θ_0_+θ_1_log(disposable inc.)^2^

Notes: Red line shows mean disposable HH income in the corrected income distribution: i) 128,482 and ii) 127,674.

Source: Own analysis of ENIGH 2010, LIS database.

Figure A3-5. Lorenz curve: income per capita, uncorrected versus unit-nonresponse corrected weights (model 1)


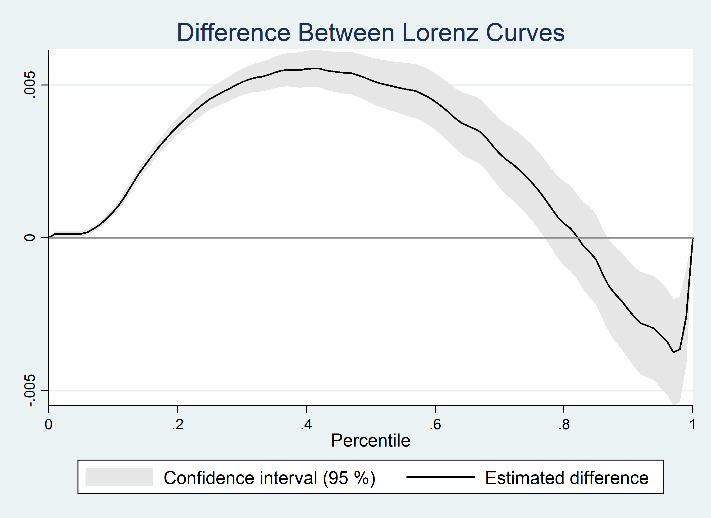

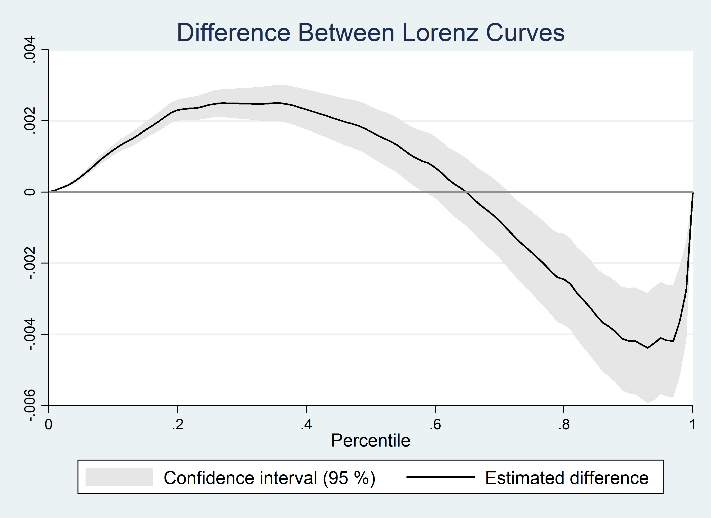


i. Disposable income net of transfers, per capita ii. Disposable income per capita

Notes: Positive values indicate that the Lorenz curve uncorrected for unit-nonresponse dominates and shows less inequality than the corrected Lorenz curve, and vice versa. Distributions account for sampling weights and household size.

Source: Own analysis of ENIGH 2010, LIS database.

Figure A3-6. Lorenz curve: disposable income vs. disposable income net of transfers, uncorrected versus corrected income distributions


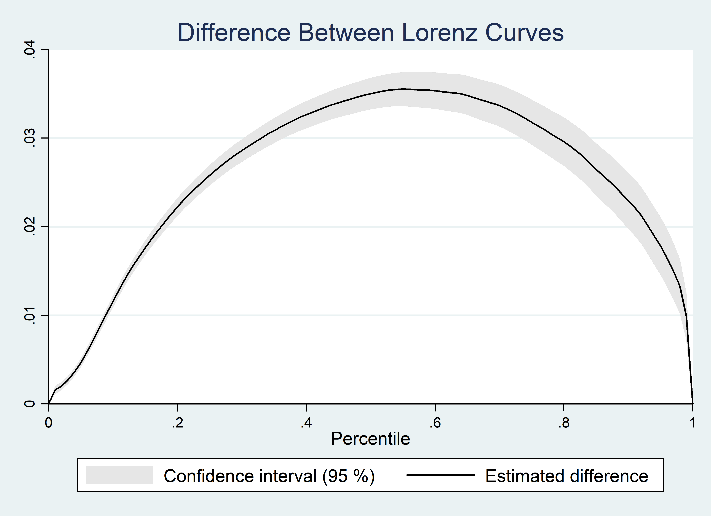

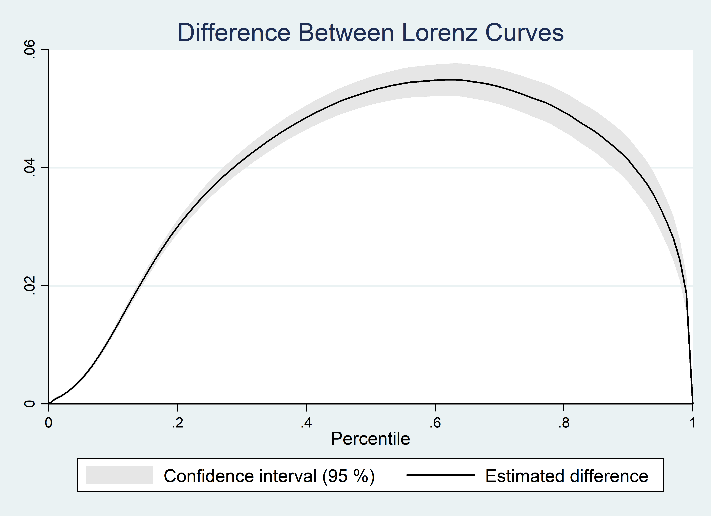


i. ENIGH sampling weights uncorrected for nonresponse ii. Weights corrected for unit nonresponse

Notes: Positive values indicate that disposable income Lorenz curve dominates, and shows less inequality than Lorenz curve of disposable income net of transfers. Distributions account for sampling weights and household size.

Source: Own analysis of ENIGH 2010, LIS database.

Figure A3-7. Comparison of Pareto I and Pareto II models, various income concepts and top income cutoffs


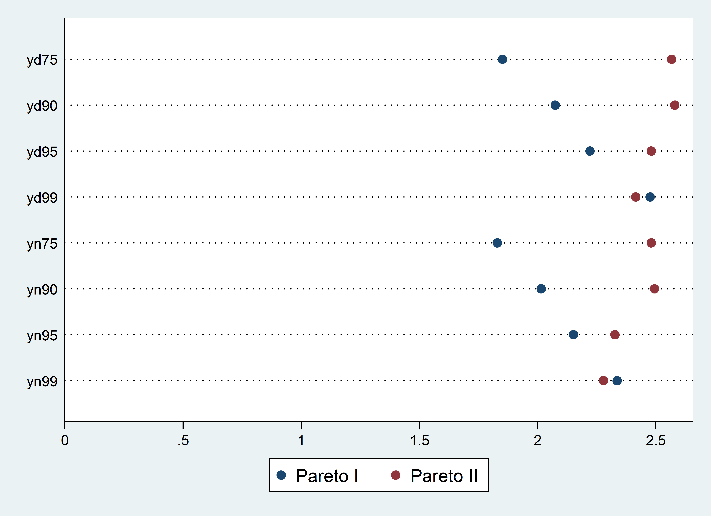

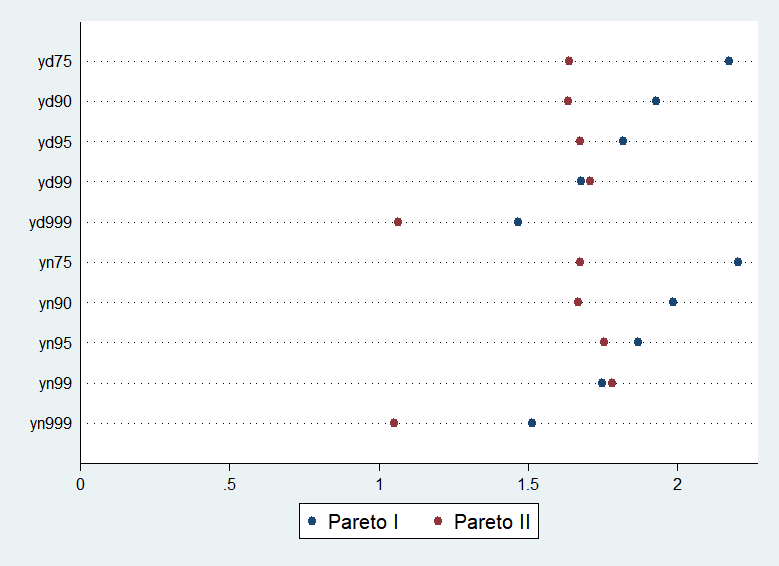


i. Pareto coefficient ii. Inverted Pareto coefficient


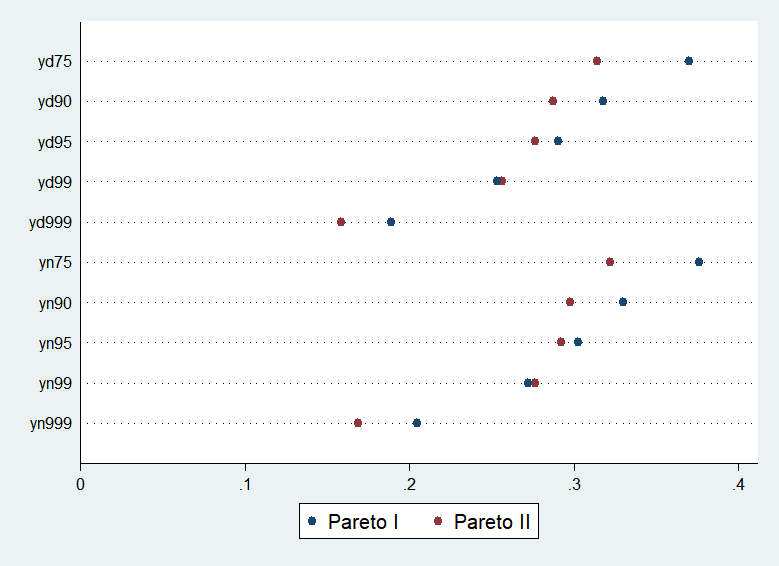


iii. Gini coefficient

Notes: ‘yn’ is disposable household income net of transfers, ‘yd’ is disposable household income, both per capita.

Source: Own analysis of ENIGH 2010, LIS database.

1. [↑](#footnote-ref-1)
2. [↑](#footnote-ref-2)
3. [↑](#footnote-ref-3)
